# Effectiveness of animal-assisted activities and therapies for autism spectrum disorder: a systematic review and meta-analysis

**DOI:** 10.3389/fvets.2024.1403527

**Published:** 2024-06-03

**Authors:** Ningkun Xiao, Vaishnavi Bagayi, Dandan Yang, Xinlin Huang, Lei Zhong, Sergey Kiselev, Mikhail A. Bolkov, Irina A. Tuzankina, Valery A. Chereshnev

**Affiliations:** ^1^Department of Immunochemistry, Institution of Chemical Engineering, Ural Federal University, Yekaterinburg, Russia; ^2^Laboratory for Brain and Neurocognitive Development, Department of Psychology, Institution of Humanities, Ural Federal University, Yekaterinburg, Russia; ^3^Guang’an District Women and Children’s Hospital, Guangan, China; ^4^Doctoral Department, Russian Sports University, Moscow, Russia; ^5^Institute of Immunology and Physiology of the Ural Branch of the Russian Academy of Sciences, Yekaterinburg, Russia

**Keywords:** autism spectrum disorder, autistic disorder, animal-assisted interventions, animal-assisted therapy, animal-assisted activities and therapies, meta-analysis

## Abstract

**Background:**

Given the rising interest in complementary therapeutic strategies for autism spectrum disorder (ASD), this research aims to provide a comprehensive analysis of the impact of animal-assisted activities and therapies (AAAT) on various ASD symptoms.

**Methods:**

A meticulous search of databases, including Scopus and PubMed, was conducted to gather relevant research on AAAT for ASD. This process led to the selection of 45 studies encompassing 1,212 participants. The chosen studies were then subjected to a meta-analysis to evaluate the efficacy of AAAT in alleviating core ASD symptoms.

**Results:**

The meta-analysis revealed significant improvements in several core ASD symptoms due to AAAT. Notably, there were improvements in social communication (MD = −4.96, 95% CI [−7.49, −2.44]), irritability (MD = −2.38, 95% CI [−4.06, −0.71]), hyperactivity (MD = −4.03, 95% CI [−6.17, −1.89]), and different word usage skills (MD = 20.48, 95% CI [7.41, 33.55]). However, social awareness (MD = −1.63, 95% CI [−4.07, 0.81]), social cognition (MD = −3.60, 95% CI [−9.36, 2.17]), social mannerisms (MD = −0.73, 95% CI [−2.55, 1.09]), social motivation (MD = −1.21, 95% CI [−2.56, 0.13]), lethargy (MD = −1.12, 95% CI [−3.92, 1.68]), and stereotypical behaviors (MD = −0.23, 95% CI [−1.27, 0.80]) did not significantly improve.

**Conclusion:**

The study demonstrates the potential of AAAT in improving certain core symptoms of ASD, such as social communication, irritability, hyperactivity, and word usage skills. However, the effectiveness of AAAT in other ASD symptom domains remains uncertain. The research is limited by the absence of long-term follow-up data and a high risk of bias in existing studies. Therefore, while the findings indicate the promise of AAAT in specific areas, caution is advised in generalizing its efficacy across all ASD symptoms.

## 1 Introduction

Autism Spectrum Disorder (ASD) encompasses a suite of neurodevelopmental conditions characterized by aberrancies in social behavior, cognition, communication, language, interests, sensory integration, and regulation, as well as repetitive and restrictive behaviors (1, 2). These features engender significant barriers for individuals with ASD, impeding their active participation in conventional social activities and physical exercise. Such limitations not only constrain their learning capabilities (3, 4), but also exacerbate other health risks, including skeletal deficiencies (5), heightened obesity rates (6), and compromised sleep disorders (7). Consequently, these impediments culminate in a holistic imbalance in the overall development of the individual (8). Thus, the primary focus of therapeutic interventions for ASD is to ameliorate the core symptoms and to augment the patients’ engagement in social activities. Existing non-pharmacological approaches, such as probiotic treatments, cognitive behavioral therapy (CBT) (9), mindfulness therapy, music therapy (10), and aerobic and motor skills exercises (11, 12), have yielded modest improvements in these core symptoms (13).

Recent research has unearthed that individuals with ASD do not exhibit a marked deficiency in their affinity for animals (14). Animal-assisted activities and therapies (AAAT) offer a unique modality of non-verbal communication, making it particularly suited for this demographic (15). The AAAT framework comprises animal-assisted therapy (AAT) and animal-assisted activities (AAA) (16). While AAT involves planned, structured, and goal-oriented interventions executed by professionals within their area of expertise in conjunction with animals, AAA is characterized by more casual interactions with animals. AAAT has been efficacious in enhancing behavioral, emotional, social, cognitive, and perceptual functioning among various populations including those with behavioral and emotional disorders (17), cerebral palsy (18), Parkinson’s Disease (19), and veterans (20, 21). Increasingly, evidence suggests that children with ASD can form intimate bonds with animals (22), and such interactions provide crucial sensory and social stimulation (23), thereby enhancing both physiological and psychological experiences for individuals with ASD (24) and significantly fostering their social participation in everyday settings (25, 26). These findings have contributed to the exponential development of social support theories related to Human-animal interaction (HAI) and validate the role of animals in both directly and indirectly boosting human social support (27). Consequently, AAAT is becoming a promising and widely accepted therapeutic approach within families coping with ASD (28).

Specifically, research conducted by Sams et al. (29) found that children with ASD exhibited elevated levels of social engagement compared to those in traditional occupational therapy courses. Furthermore, studies by Bass et al. (30), Taylor et al. (31), and Gabriels et al. (32) substantiated that therapeutic horseback riding (THR) and hippotherapy could ameliorate sensory sensitivities, enhance social motivation, and diminish stereotypical behaviors in children with ASD. Ward et al. (33) clarified that social communication and sensory processing capabilities in children with ASD were improved during the intervention period. Llambias et al. (8) reported a significant elevation in social engagement among children with ASD who participated in equine-assisted occupational therapy (EAOT), a benefit that persisted in subsequent follow-ups. Wijker et al. (34) found that dog-assisted therapy could alleviate perceptual stress and symptoms of agoraphobia in adult ASD patients, effectively enhancing their social awareness and communicative aptitudes. Conversely, some studies argue that AAAT for ASD are either not significantly effective or entirely ineffective. For example, Jenkins et al. (35) found no improvements in on-task behavior among children with ASD during animal-assisted interventions, a sentiment echoed by Hill et al. (28), who emphasized that there was no significant enhancement in on-task behavior when compared to traditional occupational therapy.

In summary, although rapidly growing research evidence supports the effectiveness of AAAT in treating ASD, current findings remain fragmented and unstratified due to varying factors such as experimental design, sample selection, outcome measurement, and lack of longitudinal follow-up. Such limitations have impeded the comprehensive compilation of scientific evidence that supports the effectiveness of these interventions, thereby restricting their broader dissemination and implementation. Therefore, this study aims to employ systematic review and meta-analysis to evaluate the effectiveness of AAAT for the core and principal associated symptoms of ASD. In doing so, we seek to provide a more nuanced, evidence-based perspective on the therapeutic elements of AAAT for ASD, thereby contributing to the body of scientific literature that informs targeted interventions.

## 2 Methods

### 2.1 Literature search

Three authors meticulously adhered to the guidelines for systematic reviews and meta-analyses (36, 37) and conducted an exhaustive search in two databases: Scopus and PubMed, with the search culminating on August 10, 2023. The search terms were designed based on strategies developed by Hedges’ team (38), encompassing types and means of intervention—such as AAI, including commonly practiced Equine-Assisted Therapy (EAT), Dog-Assisted Therapy (DAT), etc.—target population: ASD, and article types: articles published in English-language peer-reviewed journals (for detailed search strategy, see Appendix 1). In addition, supplementary searches were conducted on Google Scholar,^1^ and related systematic reviews and meta-analyses were reviewed to ensure comprehensive inclusion of qualified publications. Following the search, two authors independently reviewed the titles and abstracts of the included articles before proceeding to full-text evaluation. In cases of discrepancy, a third author would make the final decision on inclusion. All screening processes were conducted using Excel (Microsoft, Redmond, Washington State, United States of America) and Endnote X9 (Clarivate Analytics, California State, United States of America) (for included and excluded literature, see Appendices 2–4). The detailed protocol was registered on PROSPERO with registration number: CRD42023431425 (for adjustments, see Appendix 5).

### 2.2 Inclusion and exclusion criteria

The study rigorously followed the recommended PICO (Participant-Intervention-Comparison-Outcome) criteria for defining inclusion and exclusion standards. (1) Participants: individuals with a clinical or professionally diagnosed ASD, in accordance with the Diagnostic and Statistical Manual of Mental Disorders (DSM), International Classification of Diseases (ICD), Autism Diagnostic Observation Schedule (ADOS), or Social Communication Questionnaire (SCQ). No age restrictions were applied in our study. (2) Interventions: AAI, including commonly used therapy animals like horses, dogs, and dolphins. (3) Comparisons: the literature should include comparisons between AAAT and other treatments or a blank control. Pre- and post-comparisons are also eligible for inclusion. (4) Outcomes: the selected studies must indicate the improvement in at least one core or common symptom of ASD, such as overall symptom severity, social function deficits, or restrictive/repetitive behaviors. Additionally, we will also analyze other traits significantly correlated with ASD, like global cognitive ability (IQ), executive function (adaptive behavior), language expressive ability, and other disruptive or abnormal behaviors associated with ASD. (5) Types of literature: the literature should comprise clinical trial articles published in peer-reviewed journals in English, including randomized controlled trials (RCTs) and observational studies. Given the high probability of non-randomized controlled trials (non-RCTs) in this field, as indicated by our preliminary analyses, our study also included non-RCTs to provide a comprehensive evaluation of the overall intervention effectiveness.

### 2.3 Data extraction

All included studies will be imported into Excel (Microsoft, Redmond, Washington State, United States of America) for data extraction, segregated into four primary categories: (1) Bibliographic information: this includes the first author, publication year, and journal of the study. (2) Participant information: comprises the diagnostic outcome of the population involved (e.g., ASD, ADHD), the diagnostic criteria used (e.g., DSM, ICD), the total number of subjects in the experimental and control groups, as well as the sample’s mean age, age range, and sex. It further includes the sample’s mean IQ and IQ range. (3) Experimental Information: This involves the type of intervention and control (e.g., EAT, DAT), the kind of study (RCT or Non-RCT), the duration of the intervention, the outcomes post-intervention, and the effect sizes, standard errors (SE), and confidence intervals (CI). It also contains information about the review or approval authorities for the intervention. (4) Quality of Included Studies: Incorporates an analysis of publication bias, quality assessment measures, registration protocols, and data availability.

### 2.4 Data analysis

Upon data extraction, we will utilize Stata 17 (Stata Corp, Texas State, United States of America) for data analysis. It is noteworthy that in this study, the mean difference (MD) will be uniformly used as the outcome measure. Thus, we will primarily extract the mean and standard deviation (SD) from the original articles to compute the MD. If the original articles report only the standardized mean difference (SMD), these will be converted into MD. When the meta-analysis includes multiple outcomes, standard summarization methods will be employed. Additionally, the study adheres to the common I^2^ assessment for data heterogeneity. If I^2^ > 50%, we consider the heterogeneity to be high and will conduct heterogeneity tests or subgroup analyses; if I^2^ < 50%, the heterogeneity is considered within a reasonable range.

### 2.5 Assessing the credibility of evidence

Given that both RCTs and Non-RCTs are included in this study, we will employ the Physiotherapy Evidence Database scale (PEDro) and the Cochrane Risk of Bias Tool (RoB) to evaluate the quality of RCTs, and the ROBINS-I (Risk of Bias In Non-randomized Studies – of Interventions) scale for the quality of the included non-RCTs. The PEDro scale is specifically designed to evaluate the methodological quality of RCTs in the field of physical therapy. Comprising 11 items, including random assignment, concealment of allocation, baseline similarity, and blinding of patients, therapists, and assessors, among others, this method provides an accurate reflection of the internal validity of trial outcomes. The RoB covers seven specific areas of bias, including random sequence generation, allocation concealment, and blinding of participants and personnel, offering a nuanced examination of potential biases in RCTs, thus complementing the PEDro scale and enhancing the robustness of the review. ROBINS-I assesses bias in seven domains: confounding, participant selection, classification of interventions, deviations from intended interventions, missing data, measurement of outcomes, and selection of reported results, thereby providing a comprehensive overview of the reliability and validity of non-RCTs, and ensuring their results are appropriately weighted in the meta-analysis. Combining these tools allows for a thorough, detailed evaluation of both randomized and non-randomized controlled trials, ensuring that potential sources of bias are considered in our analysis, thereby providing a foundation for the quality and strength of the evidence.

## 3 Results

### 3.1 Descriptive analysis

**Figure 1:**
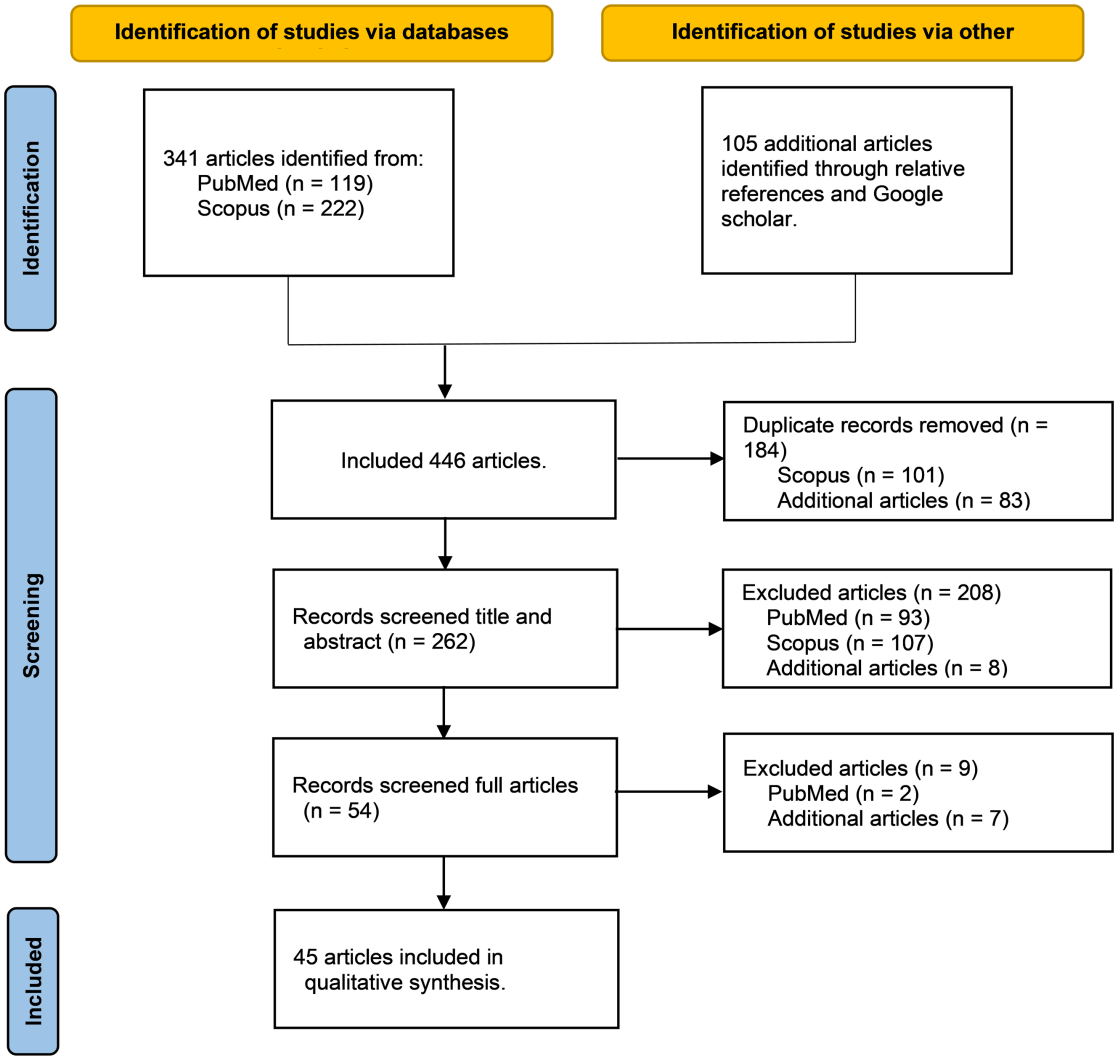
Flow diagram of systematic review and meta-analysis.

A comprehensive search of the Scopus and PubMed databases yielded 341 publications that met the initial inclusion criteria. Subsequently, a stringent selection process narrowed this to 38 articles that satisfied the final inclusion standards (for details on inclusion and exclusion, see Appendices 4, 5). Additionally, during the review of 57 previously published systematic reviews and meta-analyses, seven more publications were included (see Figure 1). Ultimately, 45 studies met the selection criteria. These 45 studies encompassed 1,212 ASD patients, averaging approximately 27 individuals per study; specifically, 859 were male, 328 were female, and three studies included 25 individuals without reporting sex. Among the participants, 51 were adults, 1,077 were minors, and 89 participants were unclassifiable as either. Regarding the duration of the interventions, 7 studies (15.22%) did not report this data. The remaining 38 studies had intervention durations ranging from 120 to 1,920 min, totaling 25,705 min, with an average intervention time per study of 676 min (for detailed information, see Table 1 and Supplementary Table S1). The animals used for assisted therapy included dogs, horses, guinea pigs, and dolphins. Horses were the most used species, featured in 32 out of the 45 studies, constituting 71.11% of all studies, followed by dogs at 20%. Regarding geographical distribution, 18 studies (40%) were conducted in the USA, 5 in Spain (11.11%), and 3 each in China and Australia. Developmental countries accounted for only 17.78% of all the research, suggesting a significant imbalance in the distribution of such studies.

**Table 1:** Sample characteristics.

First author, publish year, journal	Study ID	Reported inclusion/exclusion of participants	Sample size	Sex (male/female)	Age (mean ± SD)	Age group	IQ (mean ± SD)	Diagnosis	Diagnosis criterion	Recent AAT experience
B Caitlin Peters, 2022, J Autism Dev Disord	1	Y	*n* = 21 OTEE: 12 RA: 9	Male: 16 Female: 5 OTEE: 10/2 RA: 6/3	6–13 OTEE: 8.68 ± 2.09 RA: 9.45 ± 1.62	Group2	OTEE: 102.17 ± 12.46 RA: 78.33 ± 11.35	ASD	ADOS2 SCQ	Without experiences with EAT for 6 months
Rezapour-Nasrabad, R.R， 2022， Journal of Advanced Pharmacy Education and Research	2	Y	*n* = 8	Male: 7 Female: 1	8–13 10.62 ± 1.68	Group2	(−)	ASD	Clinician	(−)
Zhao, M, 2022, International Journal of Mental Health Promotion	3	Y	*n* = 53 THR: 26 CG: 27	Male: 33 Female: 20 THR: 15/11 CG: 18/9	5–10 THR: 6.0 ± 1.7 CG: 5.8 ± 1.5	Group1 & 2	(−)	ASD	DSM-5	(−)
Abadi, M.R.H., 2022, Anthrozoos	4	Y	*n* = 18	Male: 15 Female: 3	6–14 10.1 ± 2.5	Group2	(−)	ASD	(−)	(−)
Mengxian Zhao, 2021, Int J Environ Res Public Health	5	Y	*n* = 61 THR: 31 RA: 30	Male: 44 Female: 17 THR: 21/10 RA: 23/7	6–12 THR: 7.06 ± 1.50 RA: 7.13 ± 1.36	Group2	(−)	ASD	DSM-5	(−)
Leonardo Zoccante, 2021, J Clin Med	6	Y	*n* = 15	Male: 13 Female: 2	7–15, 9.8 ± 2.2	Group2	(−)	ASD	ADOS2	(−)
Carolien Wijker, 2021, Gen Hosp Psychiatry	7	Y	*n* = 51 AAT: 25 CG: 26	Male: 29 Female: 22 AAT: 12/13 CG: 17/9	AAT: 38 ± 12.49 CG: 39.96 ± 10.34	Group3	AAT: 103.08 ± 13.69 CG: 101.42 ± 14.17	ASD	DSM-5 WAIS PSS SCL-90-R	(−)
Peters, B.C., 2021, Research in Autism Spectrum Disorders	8	Y	*n* = 23 OTEE: 12 OT: 11	Male: 17 Female: 6 OTEE: 7/4 OT: 10/2	6–13 OTEE: 8.94 ± 2.46 OT: 9.64 ± 1.52	Group2	OTEE: 101.17 ± 13.92 OT: 83.45 ± 16.95	ASD	SCQ ADOS SRS ABC-C Leiter-3	Without EAAT for two hours or more in the last 6 months
Hernández-Espeso, 2021, Anthrozoos	9	Y	*n* = 43 DAT: 24 TWD: 19	Male:33 Female: 10 DAT: 19/5 TWD: 14/5	DAT: 4.4 ± 0.86 TWD: 4.49 ± 0.53	Group1 & 2	(−)	ASD	(−)	(−)
Jessica Hill, 2020, J Autism Dev Disord	10	Y	*n* = 22 CAOT: 11 CG: 11	Male: 16 Female: 6 CAOT: 8/3 CG: 8/3	4–6 CAOT: 5.9 ± 0.74 CG: 5.2 ± 0.93	Group1 & 2	(−)	ASD	DSM-5 CABTA ADOS2	(−)
Carolien Wijker, 2020, J Autism Dev Disord	11	Y	*n* = 52 DAT: 26 CG: 26	Male: 29 Female: 23	18–60	Group3	102.1 ± 13.7 (80–160)	ASD	DSM-5 PSS SCL-90-R ADI-R WAIS	N
B Caitlin Peters, 2020, OTJR (Thorofare N J)	12	Y	*n* = 6	(−)	6–13	Group2	> 55	ASD	SCQ ADOS Leiter-3 ABC-C	Without EAAT for two hours or more in the last 6 months
Adriana Ávila-Álvarez, 2020, Health Soc Care Community	13	Y	*n* = 55	Male: 27 Female: 28	2–16	Group1 & 2	(−)	(−)	(−)	(−)
Isabel Morales-Moreno, 2020, Holist Nurs Pract	14	Y	*n* = 16 DAT: 8 RA: 8	(−)	9–38, 20 ± 15	Group2 & 3	(−)	ASD	(−)	(−)
Portela-Pino, I., 2020， Journal of Human Sport and Exercise	15	N	*n* = 5	Male: 4 Female: 1	8–31	Group2 & 3	(−)	ASD	DSM	(−)
Kalmbach, D.， 2020， Occup Ther Health Care	16	(−)	*n* = 4	Male: 4 Female: 0	8–13	Group2	100 ± 15	ASD	ABAS	(−)
Ozyurt, Gonca., 2020, Montenegrin Journal of Sports Science and Medicine	17	(−)	*n* = 24 EAA: 12 CG: 12	Male: 17 Female: 7 EAA: 8/4 CG: 9/3	4–12, 6.77 ± 1.3 EAA: 6.75 ± 0.7 CG: 6.7 ± 0.64	Group1 & 2	(−)	ASD	(−)	Without previous experience with EAA
Monique M Germone, 2019, Autism	18	Y	*n* = 66	Male: 53 Female: 13	4–17	Group1 & 2	(−)	ASD	SCQ ADOS-2	(−)
Ana L L Michelotto, 2019, J Altern Complement Med	19	Y	*n* = 15	Male: 14 Female: 1	2–12 5.6 ± 1.6	Group1 & 2	(−)	ASD	DSM-5	(−)
Kwon, S., 2019, Ann Rehabil Med	20	Y	*n* = 29 THR: 18 CT: 11	Male: 16 Female: 13 THR: 11/7 CT: 5/6	6–11 THR: 8.2 ± 1.7 CT: 7.5 ± 1.1	Group 2	(−)	ASD ID	(−)	Without experience of THR
Robin L Gabriels, 2018, Front Vet Sci	21	Y	*n* = 64 THR: 36 BA: 28	Male: 54 Female: 10 THR: 29/7 BA: 25/3	6–16 THR: 10.7 ± 2.9 BA: 9.4 ± 2.5	Group 2	85 or > 85 THR: 88.4 ± 25.1 BA: 89.2 ± 19.8	ASD	SCQ ADOS-2	(−)
Pan, Z., 2018, Front Vet Sci	22	Y	*n* = 16 THR: 8 BA: 8	Male: 13 Female: 3 THR: 6/2 BA: 7/1	6–16 THR: 11.88 ± 2.45 BA: 9.80 ± 2.82	Group 2	THR: 102.88 ± 16.28 BA: 100.25 ± 29.26	ASD	SCQ ADOS-2 ABC-C Leiter-3	(−)
Tan, V. X., 2018, J Autism Dev Disord	23	(−)	*n* = 6	Male: 1 Female: 5	3–14	Group1 & 2	IQ: 40 and 56	ASD	(−)	(−)
Androulla Harris, 2017, J Environ Res Public Health	24	Y	*n* = 26 HR: 12 CG: 14	Male: 22 Female: 4 HR: 9/1 CG: 12/ 2	6.08–9.33, 7.5 ± 10.57 HR: 8.2 ± 10.56 CG: 7 ± 3.95	Group 2	(−)	ASD	DSM CARS2 ABC-C	(−)
Cecilia Llambias, 2016, Am J Occup Ther	25	Y	*n* = 7	Male: 4 Female: 3	4–8	Group1 & 2	(−)	ASD, ADHD	(−)	Without riding experiences for 3 months before the study
Marta Borgi, 2016, J Autism Dev Disord	26	Y	*n* = 28 EAT: 15 CG: 13	(−)	6–12, 8.6 ± 1.7 EAT: 9.2 ± 1.8 CG: 8.0 ± 1.5	Group 2	IQ > 70 EAT: 98.3 ± 16.2 CG: 92.8 ± 19.9	ASD	DSM ICD-10	(−)
Sophie Anderson, 2016, J Autism Dev Disord	27	Y	*n* = 15	Male: 11 Female: 4	5–16, 10 ± 3.8	Group 2	(−)	ASD	DSM	(−)
Robin L Gabriels， 2015, J Am Acad Child Adolesc Psychiatry	28	Y	*n* = 116 THR: 58 BA: 58	Male: 101 Female: 15 THR: 49/9 BA: 52/6	6–16 THR: 10.5 ± 3.2 BA: 10.0 ± 2.7	Group2	THR: 86.7 ± 25.5 BA: 86.1 ± 22.7	ASD	DSM-5	Without more than two hours of EAT within the past six months
H Steiner, 2015, Acta Physiol Hung	29	Y	*n* = 26 THR: 13 CG: 13	Male:12 Female: 14 THR:6/7 CG: 6/7	10–13	Group2	(−)	ASD	(−)	Control period of three months without any horse therapy
Beth A Lanning, 2014, J Autism Dev Disord	30	Y	*n* = 25 EAA: 13 CG: 12	Male: 21 Female: 4 EAA: 9/4 CG: 12	4–15 EAA: 4–15, 7.5 ± 3.2 CG: 5–14, 9.8 ± 3.2	Group 1 & 2	(−)	ASD	(−)	Without experiences with EAA during 6 months
Marguerite E O'Haire, 2014, J Altern Complement Med	31	N	*n* = 64	Male: 50 Female: 14 Waitlist: 28/9 non-waitlist: 22/5	5–12, 8.9 ± 2.2 Waitlist: 9.5 ± 2.4 non-waitlist: 8.2 ± 1.7	Group 1 & 2	(−)	ASD, Asperger, Pervasive Developmental Disorder	(−)	(−)
Margo B Holm, 2014, J Autism Dev Disord	32	Y	*n* = 3	Male: 3 Female: 0	6–8	Group2	(−)	ASD	Clinician physician	Riding experience once a week for approximately 1 year
Fung, S.-C., 2014, Journal of Contemporary Psychotherapy	33	Y	*n* = 10 AAPT = 5 CG = 5	Male: 8 Female: 2	7–10, 8.9	Group2	(−)	ASD	DSM	(−)
Heather F Ajzenman, 2013, Am J Occup Ther	34	Y	*n* = 7	Male: 4 Female: 3	5–12 8.4 ± 2.5	Group1 & 2	(−)	ASD	DSM	Excluded participants with previous exposure to any type of EAAT
Sandra C Ward, 2013, J Autism Dev Disord	35	Y	*n* = 21	Male: 15 Female: 6	8.1	Group2	(−)	ASD	DSM CAB-T	Thirteen of the participants without experience of TR
Ghorban, Hemati.， 2013, Journal of Education and Learning	36	Y	*n* = 6	Male: 1 Female: 5	6–12	Group2	(−)	ASD	(−)	(−)
Jenkins, Sarah R., 2013, Research in Autism Spectrum Disorders	37	Y	*n* = 7 THR: 4 CG: 3	Male: 6 Female: 1	6–14, 9.5	Group2	(−)	ASD	VABS	Without Experience with EAT
Emílio Salgueiro, 2012, BMC Res Notes	38	(−)	*n* = 10	Male: 8 Female: 2	3–16	Group1 & 2	(−)	ASD	(−)	(−)
Tabares, C., 2012, Neurochemical Journal	39	(−)	*n* = 8	Male: 8 Female: 0	8–16	Group2	(−)	ASD	(−)	(−)
MdYusof, 2012, International Journal of Special Education	40	N	*n* = 15	Male: 10 Female: 5	9–10	Group2	90–110	ASD	Clinical psychologists	(−)
Gabriels, Robin L., 2012, Research in Autism Spectrum Disorders	41	Y	*n* = 42 THR: 26 CG: 16	Male: 36 Female: 6 THR: 21/5 CG: 15/1	6–16, 8.7 THR: 6–16, 8.6 CG: 6–14, 8.8	Group2	95.2, 44–139	ASD Asperger Seizures	ABC-C SCQ ADOS Leiter-3	Without experience of THR within the past three years
Janet K Kern, 2011, Altern Ther Health Med	42	Y	*n* = 24	Male:18 Female: 6	3–12, 7.8 ± 2.9	Group1 & 2	(−)	ASD	CARS	Without previous participation in EAA
Robert Viau, 2010, Psychoneuroendocrinology	43	Y	*n* = 42	Male: 37 Female: 5	3.6–14.8, 7.1 ± 3.1	Group1 & 2	(−)	ASD, Asperger PDDNOS	Clinician	(−)
Taylor, Renee R., 2009, Occupational Therapy in Mental Health	44	(−)	*n* = 3	(−)	4–6	Group1 & 2	(−)	ASD	(−)	(−)
Bass, M. M., 2009, J Autism Dev Disord	45	Y	*n* = 34 THR: 19 CG: 15	Male: 29 Female: 5 THR: 17/2 CG: 12/3	5–10 THR: 6.95 ± 1.67 CG: 7.7 3 ± 1.65	Group1 & 2	(−)	ASD Asperger	DSM	Without experiences with EAA

This table is sorted by chronology and abbreviation in alphabetical order. (−), not reported; Y, yes; N, No; CG, control group; age group: group1, preschool children (0–5 years old); group2, school-age children and adolescents (6–18 years old); gropu3, adults (>18 years old). EAT, Equine-assisted therapy; THR, Therapeutic Horseback Riding; DSM-5, Diagnostic and Statistical Manual of Mental Disorders-5; RA, Regular Activities; CABTA, Children’s Attitudes and Behaviors Towards Animals; CAOT, Canine Assisted Occupational Therapy; ADOS, Autism Diagnostic Observation Schedule; ADHD, Attention deficit hyperactivity disorder; PSS, Perceived Stress Scale; SCL-90-R, Symptom Checklist-90-Revised; ADI-R, Autistic disorder interview-revised; WAIS, Wechsler Adult Intelligence Scale III/IV; DAT, Dog Assisted Therapy; RA, Regular activity; OTEE, Occupational therapy in an equine environment; SCQ, Social Communication Questionnaire; CARS, Childhood Autism Rating Scale; CAB-T, Clinical assessment battery teacher rating form; AAPT, Animal-Assisted Play Therapy; AAA, Animal-Assisted Activities; ID, Intellectual Disability; ABAS, Adaptive Behavior Assessment System; TWD, Therapy Without Dolphins.

**Figure 2:**
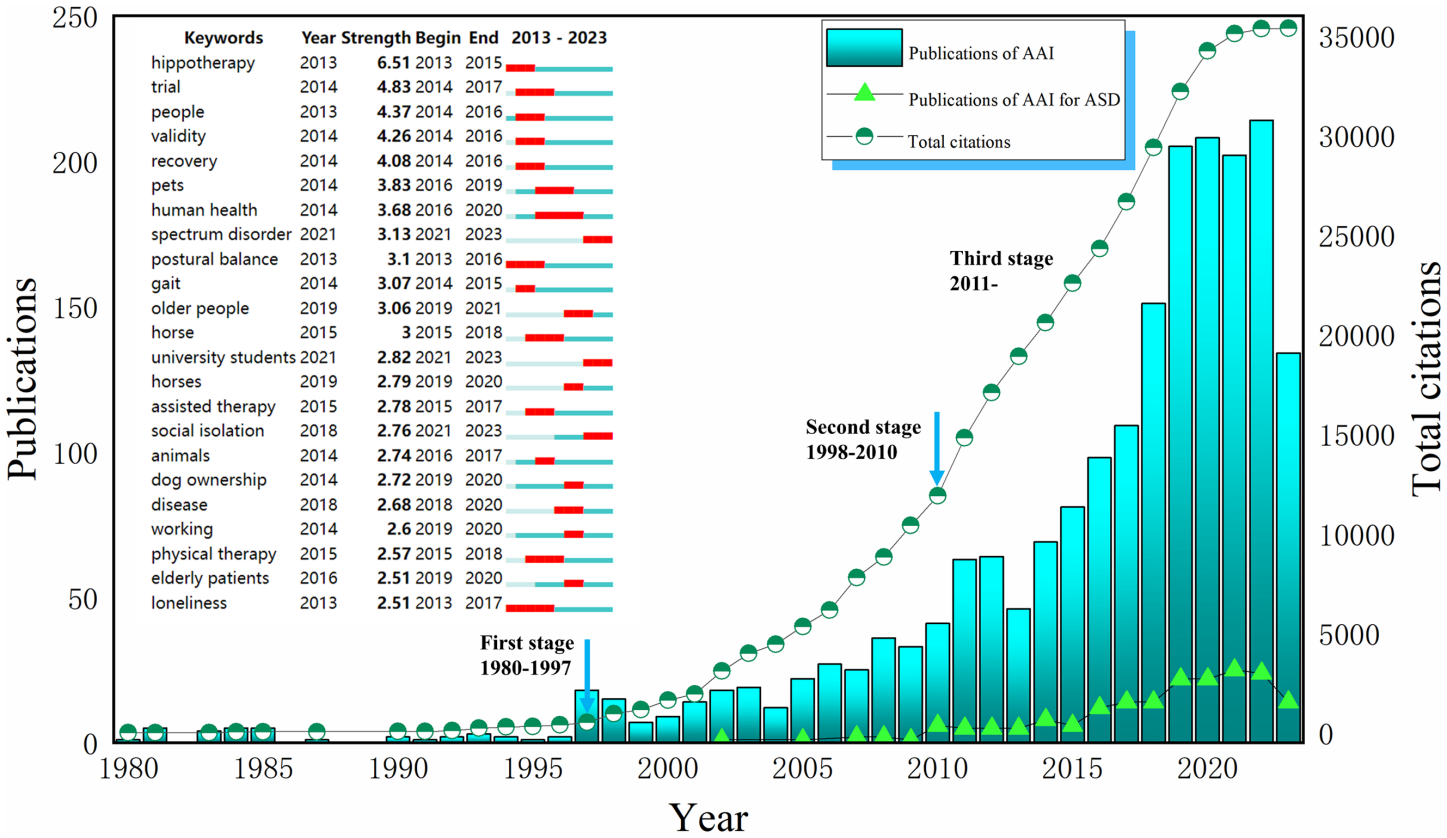
The number of publications and keywords citation bursts. AAI, Animal-assisted interventions; ASD, Autism spectrum disorder. Search terms in Web of Science (SCIE and SSCI): (1) TS = (“Animal Assisted Therapy”) OR TS = (“Animal Assisted Therap*”) OR TS = (“Therap*, Animal Assisted”) OR TS = (“Animal Facilitated Therap*”) OR TS = (“Animal Assisted Intervention*”) OR TS = (“Animal Assisted Activit*”) OR TS = (“Pet Therap*”) OR TS = (“Pet Assisted Therap*”) OR TS = (“Equine-Assisted Therapy”) OR TS = (“Equine Assisted Therap*”) OR TS = (“Equine Assisted Psychotherap*”) OR TS = (“Hippotherap*”) OR TS = (“Horseback Riding Therap*”) OR TS = (“Canine Assisted Therap*”) OR TS = (“Dog Assisted Therap*”) OR TS = (“Animal-Assisted Counseling”) OR TS = (“Animal-Assisted Education”) OR TS = (“Dolphin Assisted Therapy”) (2) TS = (“autism spectrum disorder”) OR TS = (“autistic spectrum disorder*”) OR TS = (“ASD”) OR TS = (“disorder, autistic spectrum”) OR TS = (“asperger syndrome”) OR TS = (“autis*”) OR TS = (“Pervasive Developmental Disorders”) OR TS = (“Rett syndrome”) OR TS = (“childhood disintegrative disorder”).

Moreover, a detailed examination using Web of Science revealed exponential growth in the field of AAAT from 1980 to 2022. This growth can be segmented into three phases: the first phase from 1980–1997 saw the nascent stages of AAAT, with fewer than ten publications per year and limited citation impact. The second phase spanned from 1998–2010, during which the annual publication rate increased from 2–3 to over 30, and citations swelled to 11,902 by 2010. AAAT for ASD patients started to appear in the literature around 2002. The third phase started in 2011, with even more rapid growth in research and citations, including more than 20 annual publications focused on AAAT for ASD. The analysis of the burst intensity of the keywords revealed that the outbreak of the field started from 2013, and there were 23 keywords with a burst intensity of more than 2.50, and the highest attention was paid to the means of intervention constituted by animals such as horse, dog, etc., the population of intervention constituted by spectrum disorder, disease, elderly patients, etc., and the assisted therapy, physical therapy, and other keywords (see Figure 2).

### 3.2 Quality assessment

**Figure 3:**
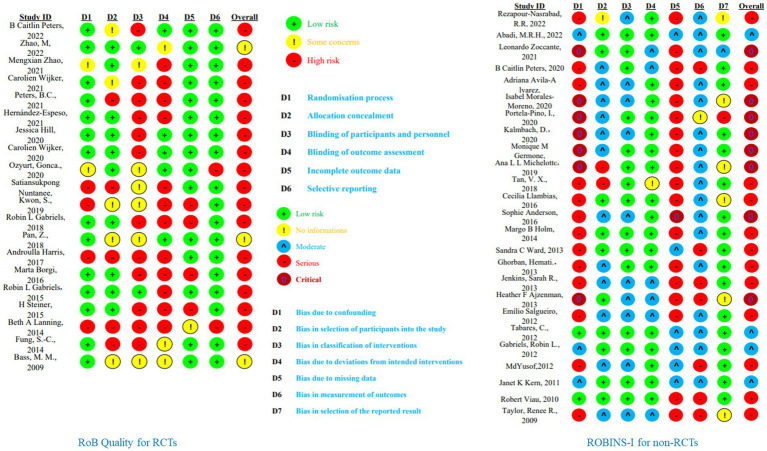
Quality assessment of included studies. This calculated by random-effects REML model; BA, barn activity; OT, occupational therapy.

Because one study was missing quality data, we evaluated 19 RCTs and 25 Non-RCTs. For RCTs, the PEDro and Cochrane Risk of Bias Tools were used for quality assessment, and for Non-RCTs, the ROBINS-I scale was employed. Among the 19 included RCTs, only three studies were free from high risk in all six subcategories: random sequence generation, allocation concealment, blinding of participants and personnel, blinding of outcome assessment, incomplete outcome data, and selective reporting. The remaining 17 studies had some level of high risk, especially in the subcategories of blinding of participants and personnel and blinding of outcome assessment. This is mainly due to the inherent challenges of conducting double-blind experiments in this type of research (see Figure 3). According to the PEDro scale, eight studies scored 7 points and were considered high quality, eleven studies scored between 4–6 points and were deemed fair quality, and one study scored 3 points, indicating poor quality. For the 25 assessed non-RCTs, four were rated as moderate risk, thirteen as serious risk, and eight as critical risk. None of the included non-RCTs had a low risk level (see Figure 3, Table 2, Appendices 6, 7). Overall, the studies included in this research generally demonstrated a high risk of bias and moderate quality levels.

**Table 2:** PEDro Scoring for RCTs.

First author, publish year	Eligibility criteria specified	Random subject allocation	Allocation concealment	Baseline similarity of groups	Blinding of subjects	Blinding of therapies	Blinding of assessors	Measures of outcomes	Intention to treat	Between group comparisons	Point measures and variability measures	Total
B Caitlin Peters, 2022	Y	Y	(−)	Y	(−)	(−)	Y	Y	Y	Y	Y	7
Zhao, M, 2022	Y	Y	Y	Y	(−)	(−)	(−)	Y	Y	Y	Y	7
Mengxian Zhao, 2021	Y	Y	(−)	Y	(−)	(−)	N	Y	Y	Y	Y	6
Carolien Wijker, 2021	Y	Y	(−)	Y	N	N	N	Y	Y	Y	Y	6
Peters, B.C., 2021	Y	Y	N	N	N	N	(−)	Y	(−)	Y	Y	4
Hernández-Espeso, 2021	Y	Y	Y	Y	N	N	N	Y	Y	Y	Y	7
Jessica Hill, 2020	Y	Y	Y	N	N	N	Y	Y	Y	Y	Y	7
Carolien Wijker, 2020	Y	Y	Y		(−)	(−)	Y	Y	Y	Y	Y	7
Ozyurt, Gonca., 2020	N	Y	(−)	(−)	(−)	(−)	Y	Y	Y	Y	Y	5
Kwon, S., 2019	Y	N	N	(−)	N	N	N	Y	N	Y	Y	3
Robin L Gabriels, 2018	Y	Y	Y	Y	N	N	N	Y	Y	Y	Y	7
Pan, Z., 2018	Y	Y	(−)	Y	(−)	(−)	Y	Y	Y	Y	Y	7
Androulla Harris, 2017	Y	N	N	Y	N	N	N	Y	Y	Y	N	4
Marta Borgi, 2016	Y	Y	Y	N	N	N	N	Y	Y	Y	Y	6
Robin L Gabriels， 2015	Y	Y	(−)	Y	(−)	N	N	Y	Y	Y	Y	6
H Steiner, 2015	Y	Y	Y	Y	N	N	N	Y	Y	Y	Y	7
Beth A Lanning, 2014	Y	N	N	Y	N	N	N	N	Y	Y	Y	4
Fung, S.-C., 2014	Y	Y	N	Y	(−)	N	(−)	Y	Y	Y	Y	6
Bass, M. M., 2009	Y	Y	(−)	Y	(−)	(−)	(−)	Y	Y	Y	Y	6

Scores: 7–10 (high quality), 4–6 (fair quality), and < 4 (poor quality); Y, yes; N, No.

### 3.3 Outcome measurement instruments

In assessing the effectiveness of AAAT, standardized scales serve as the most prevalent methodologies. The 45 incorporated studies collectively employed a total of 24 distinct scales to assess core and common symptom domains in ASD patients, including social functioning and responsiveness, behavioral competencies, and motor skills, as well as stress levels.

For evaluating social functioning and responsiveness, instruments such as the Social Responsiveness Scale (SRS) (30, 34, 39–41), Pediatric Evaluation of Disability Inventory Computer Adaptive Test, Autism Spectrum Disorder Module (PEDI-CAT ASD) (41), Psychoeducational Profile Revised (PER-R) (42), Sensory Profile (SP) (30), Stone’s Social Skills Scale (SSSS) (43), and the Receptive and Expressive Vocabulary Test (REVT) (44) were commonly utilized. These instruments gauge various dimensions, such as social cognition, linguistic capabilities, and the capacity for daily social activities in ASD patients.

To assess motor functioning and behavioral competencies, scales like Aberrant Behavior Checklist–Community (ABC-C) (32), VABS (32), BOT (32), SIPT (32), Test of Gross Motor Development-Third Edition (TGMD-3) (45), Vineland (46), Developmental Coordination Disorder Questionnaire, as revised in 2007 (DCDQ’07) (46), and Interaction Emotions Motor Skills (IEMS) (46) were employed as primary tools.

Furthermore, the evaluation of stress states in ASD patients included scales that measure perceived stress, such as the Perceived Stress Scale (PSS) (34), the Symptom Checklist-90-Revised (SCL-90-R) (34) for psychological and physical symptoms, the ABC-C (39, 41) for irritability and hyperactivity, and the Rosenberg Self Esteem Scale (RSES) (34) for self-esteem levels. Physiological indicators, specifically cortisol levels, were also assessed (39, 41, 47–49).

In studies adopting descriptive statistics, measures such as the Canadian Occupational Performance Measure (COPM) (40), on-task behavior, and Goal Attainment Scaling (GAS) were primarily employed to ascertain whether AAI would impact the rate of goal achievement and task completion time among ASD participants. This served to validate the potential improvements AAI could confer on social behaviors and task performance for individuals with ASD.

### 3.4 Primary intervention outcomes: social responsiveness and functioning

**Figure 4:**
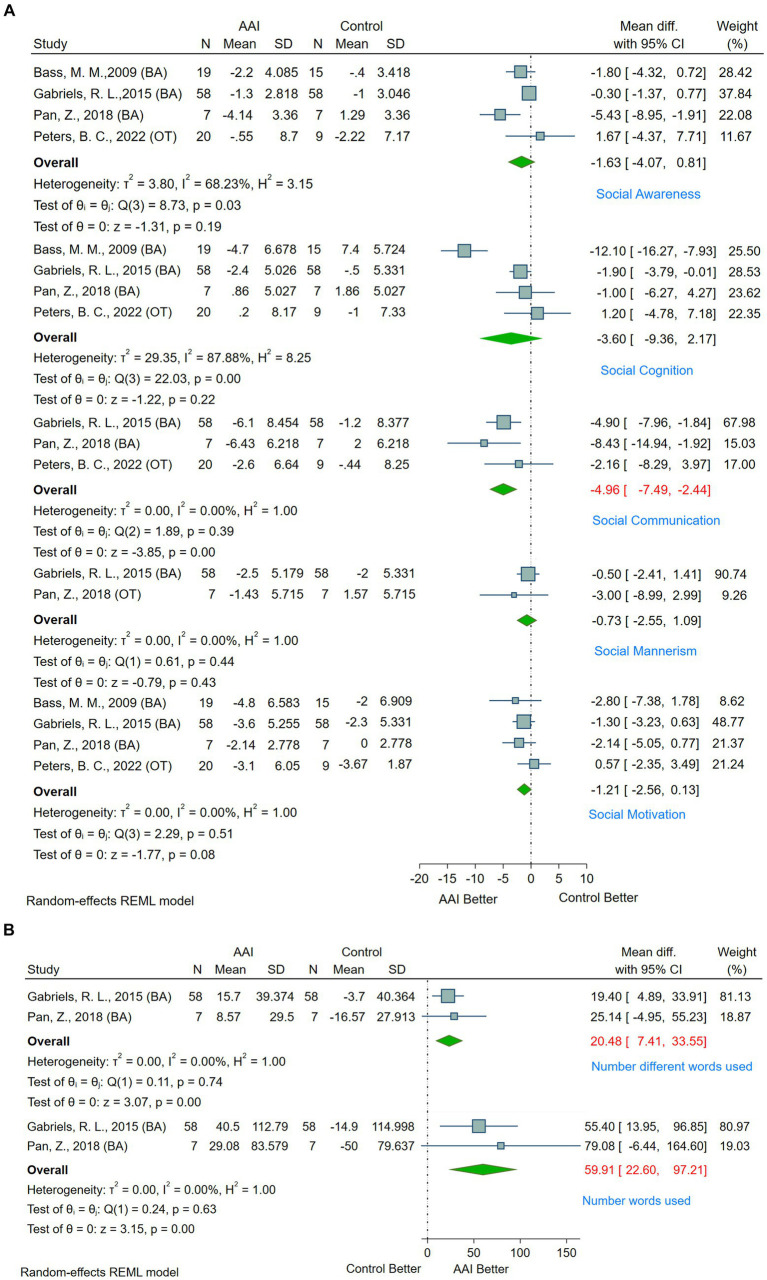
**(A)** Forest plot of social function by SRS. **(B)** Forest plot of language ability by SALT.

Social responsiveness and functioning are pivotal symptom domains in individuals with ASD. Within the scope of this study, four research articles employed the SRS to comprehensively analyze the social responsiveness and functioning of ASD patients. Of these, three studies utilized Barn Activities (BA) as the control group (25, 30, 39), and one employed Occupational Therapy (OT) (41). The SRS dissects social functioning into five sub-categories: social awareness, social cognition, social communication, social mannerism, and social motivation. According to the meta-analysis, in comparison with control groups, AAAT exhibited a significant improvement in the social communication domain for ASD patients (MD = −4.96, 95% CI [−7.49, −2.44]). However, no improvements were observed in social awareness (MD = −1.63, 95% CI [−4.07, 0.81]), social cognition (MD = −3.60, 95% CI [−9.36, 2.17]), social mannerism (MD = −0.73, 95% CI [−2.55, 1.09]), and social motivation (MD = −1.21, 95% CI [−2.56, 0.13]) (see Figure 4 and Supplementary Table S2). Even after eliminating studies with high sources of heterogeneity, adjusted results continued to show no improvements in social awareness (MD = −0.47, 95% CI [−1.44, 0.50], I^2^ = 0.00%) or social cognition (MD = −1.55, 95% CI [−3.26, 0.15], I^2^ = 0.00%) (see Appendix 8).

Another critical area of social functioning is linguistic capability, a significant impediment for ASD patients. Among the 45 studies included, only two provided comprehensive data using SALT for analyzing language abilities in ASD patients (25, 39). The analysis indicated a significant improvement in the utilization of different words (MD = 20.48, 95% CI [7.41, 33.55]) and the existing vocabulary (MD = 59.91, 95% CI [22.60, 97.21]) in ASD patients undergoing AAI (see Figure 4B).

These findings align generally with previous research, indicating improvements in social motivation and performance (30, 41, 43, 50), enhanced language expression and communication skills (32, 39, 44, 51–54), and elevated emotional understanding, empathy, and perspective-taking abilities (43, 50, 55). It also confirmed the efficacy of AAAT in enhancing social functioning (39, 56). In summary, AAI interventions appear to facilitate some improvement in the social and linguistic abilities of ASD patients. However, current meta-analytical evidence strongly supports the impact of AAI only on social communication, leaving improvements in other domains a subject of ongoing debate.

### 3.5 Behavioral capability

**Figure 5:**
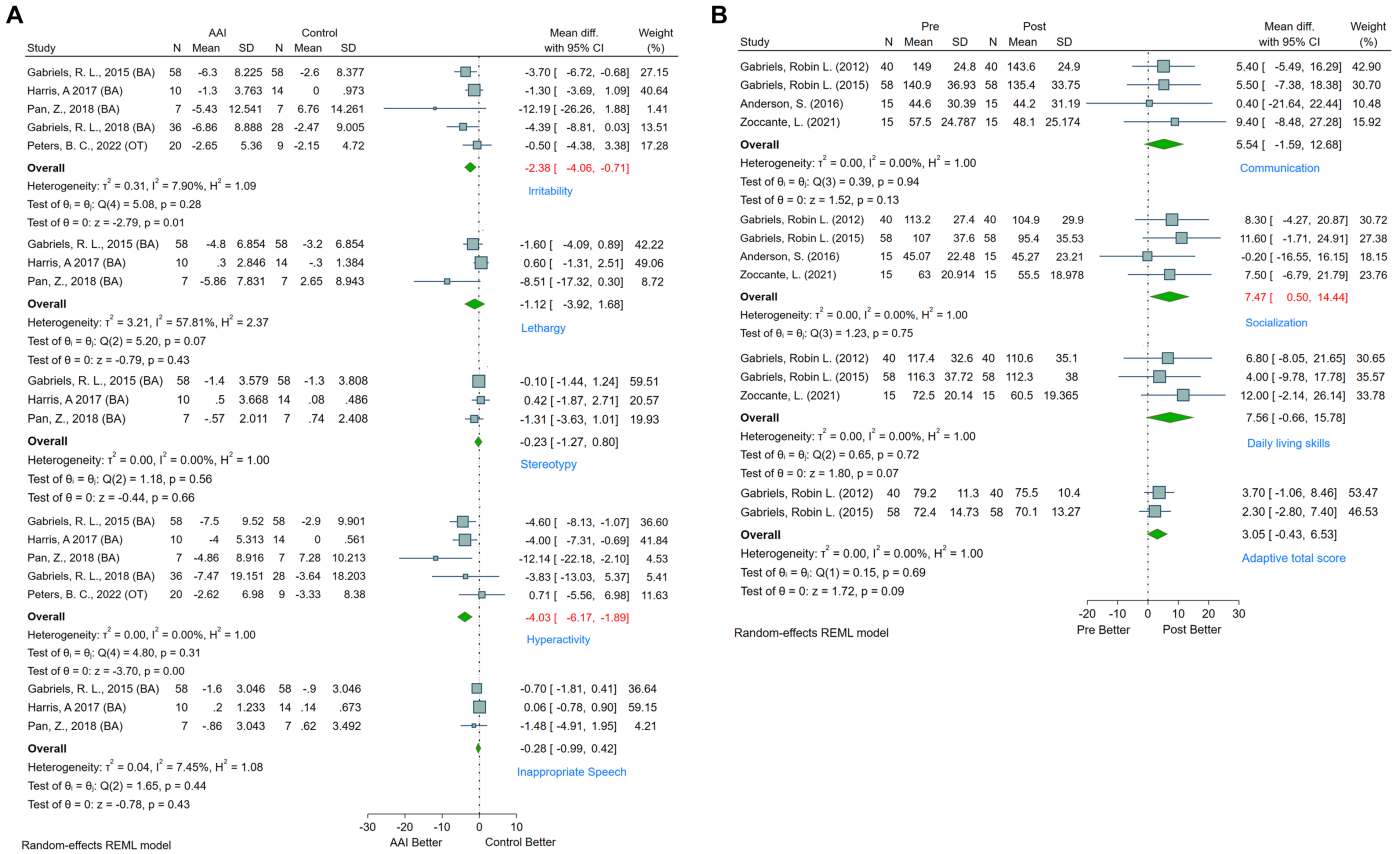
**(A)** Forest plot of behavioral and physical function by ABC-C. **(B)** Forest plot of behavioral and physical function by VABS.

Behavioral impairments are among the core symptoms manifest in individuals with ASD. Research reveals that approximately 83% of children with ASD demonstrate varying degrees of developmental delays in behavioral ability (45). Such deficits curtail the likelihood of these individuals participating in physical and social activities, exacerbating social and familial isolation and consequent anxiety (57). This further escalates the manifestation of repetitive and restrictive behaviors. The current study predominantly employed the ABC-C and VABS scales to gauge behavioral functions in ASD patients. Five studies that utilized the ABC-C and provided comprehensive data (25, 39, 41, 56, 58) were incorporated. According to meta-analysis, AAAT significantly decreased irritability (MD = −2.38, 95%CI [−4.06, −0.71]) and hyperactivity (MD = −4.03, 95%CI [−6.17, −1.89]) in ASD patients. No significant improvements were noted in subdomains, such as lethargy (MD = −1.12, 95%CI [−3.92, 1.68]), stereotypical (MD = −0.23, 95%CI [−1.27, 0.80]), or inappropriate speech (MD = −0.28, 95%CI [−0.99, 0.42]) (see Figure 5).

Additionally, four studies employed a pre-post design using VABS to measure behavioral function in ASD patients (25, 32, 46, 55). The evidence indicates significant improvements in the socialization subdomain (MD = 7.47, 95%CI [0.50, 14.44]) following AAAT, whereas no significant changes were observed in the communication (MD = 5.54, 95%CI [−1.59, 12.68]), daily living skills (MD = 7.56, 95%CI [−0.66, 15.78]), or adaptive total score (MD = 3.05, 95%CI [−0.43, 6.53]) (see Figure 5B).

These findings are consistent with earlier research that attests to the significant improvement of behavioral capacities in ASD patients through AAAT programs (25, 50, 59). Such interventions have also been found to reduce problem behaviors and aggression (60, 61), as well as enhance self-regulation, adaptive behavior, and coordination (32, 46, 54, 55, 62). Importantly, AAAT has been noted to significantly increase the minutes of mild physical activity and substantially reduce sedentary minutes in ASD patients (30, 63). However, some studies suggest that improvements in task completion rates and times in AAAT-involved ASD patients do not differ significantly from those in non-animal-involved occupational therapy control groups (28). Further evidence is needed to ascertain the comparative efficacy of AAI over other therapies.

### 3.6 Stress

Prior research has substantiated those individuals with ASD experience higher levels of stress and lower self-esteem compared to the general population (2, 34, 49). Stress positively correlates with depression, anxiety, and cardiovascular diseases (64), while self-esteem strongly inversely correlates with stress (65). Long-term exposure to high stress levels can exacerbate cognitive flexibility challenges in ASD patients, thereby hindering the acquisition of basic social skills (66). Therefore, mitigating stress can effectively improve the depressive and anxious states of ASD patients and augment their self-esteem and social interest. On stress-related outcome measures, AAAT has been shown to alleviate both physiological and subjective stress states in ASD patients as reported by O’Haire, Wijker et al., Peters et al., among others (41, 47–49). However, current evidence falls short in confirming the long-term efficacy of AAAT in stress reduction for ASD patients.

### 3.7 Parent–child interaction

The dynamics of parent–child relationships and parental stress serve as significant mediators in the treatment of individuals with ASD. Prior research has highlighted elevated levels of stress and frustration experienced by caregivers of ASD patients (67). Such stress levels are instrumental in shaping the management and caregiving strategies for ASD. Among the studies incorporated in this research, two specifically addressed this criterion. Zoccante et al. (46) found that EAT yielded no significant effect in ameliorating parent–child relationships or alleviating parental stress. Conversely, Ozyurt et al. (68) reported that AAT contributed positively to the mental health and familial functionality of mothers of children with ASD. At present, the scarcity of studies on this outcome measure makes it challenging to arrive at conclusive judgments.

### 3.8 Follow-up outcomes

Another crucial metric for assessing interventions for ASD encompasses follow-up outcomes. However, only seven studies reported on these. Llambias et al. (8) documented those children with ASD who participated in AAT programs maintained significantly improved social engagement skills. Wijker and associates (34, 58) emphasized that post-AAT, the reduction in perceived stress and agoraphobia among ASD patients continued to be effective, and improvements in social skills persisted. However, both Viau et al. and Wijker et al. noted that despite the decline in cortisol levels among ASD patients following AAT, no long-term effects were observed (48, 49).

Furthermore, Ward et al. (33), O’Haire et al. (16), and Holm et al. (69) conducted intermittent experiments, where initial AAAT sessions resulted in notable improvements in social functioning and substantial reductions in social withdrawal behaviors. However, the outcomes varied during the interlude between AAAT sessions. When AAAT was resumed, the improvement resurged. Holm et al. (69) also emphasized the significance of dosage in AAT, asserting that increasing the dosage of AAAT is instrumental for enhancing target behaviors in ASD patients. Nevertheless, no conclusive evidence is currently available to determine the optimal dosage of AAAT for maximal efficacy.

## 4 Discussion and limitations

Although most of the existing literature endorses the effectiveness of AAAT in mitigating both the core and peripheral symptoms of ASD, several critical limitations must not be overlooked. Most studies in the field are plagued by a range of issues, including but not limited to small sample sizes, minimal use of blinding methods, low levels of randomization, a multitude of confounding variables, absence of control groups, lack of follow-up, and suboptimal experimental designs. These limitations collectively restrain the generalizability and broad applicability of AAAT as an effective intervention for ASD patients.

From a methodological standpoint, the most pressing challenges in the current research landscape revolve around difficulties in implementing blinding, the presence of numerous potential confounding factors, reliance on singular measurement methods, and insufficient longitudinal data. The current research deficiencies limit the credibility of experimental results and the practical application of AAAT. It’s harder to draw broad conclusions from studies with small sample sizes because they do not have enough statistical power to look at subgroups of people with ASD based on things like age, symptom severity, or other conditions they may have. Due to the nature of the intervention, blinding in AAAT studies is inherently challenging, leading to potential biases that can exaggerate the perceived efficacy of AAAT. Furthermore, the dearth of comparative studies across diverse cultural and economic backgrounds, primarily conducted in developed countries, results in a geographical bias that limits the applicability of findings to other regions. More details, in the 42 studies included in this review, a mere eight studies (17.8%) reported whether the participants were concurrently receiving other interventions, making it difficult to tease apart the specific effects of AAAT. Similarly, only 19 studies (42.2%) employed a controlled experimental design, with 16 utilizing a pre-post design. In terms of control conditions, most studies either employed blank control or control without animal intervention. The absence of robust control groups, especially when other therapeutic interventions are not reported, compromises the external validity of the findings, thereby undermining the credibility of treatment outcomes. It is also worth noting that small sample sizes are a significant barrier to the generalizability of AAAT outcomes; Out of the 45 studies that were reviewed, 21 (46.7%) had fewer than 20 patients with ASD, nine (20%) had more than 50 patients, and the remaining 15 (33.3%) had sample sizes between 20 and 50. Moreover, the prevailing reliance on scale-based measurements poses challenges to the accurate representation of treatment effects and is incapable of eliminating confounding variables. For example, task completion time and success rates in ASD patients cannot be adequately captured by scales alone, lacking insights into emotional and cognitive investment. Additionally, the fact that most researchers serve as the evaluators in these studies raises the risk of assessment bias.

Given these constraints and the current state of research, future research should consider collaborating with multiple research centers, clinics, and community organizations to recruit larger and more diverse participant groups from different cultural and economic backgrounds. Employing stratified randomization will ensure a balanced distribution of key demographic and clinical variables between intervention and control groups. It is essential to establish comprehensive follow-up assessment systems, develop detailed data management plans, and create standardized intervention protocols to ensure consistency and replicability across studies. Incorporating mixed-method approaches, including objective outcome measures such as physiological biomarkers and standardized behavioral assessments, will enhance the rigor and reliability of research findings. Eye-tracking technology and biological markers of stress, such as heart rate and salivary cortisol and oxytocin levels, could serve as auxiliary measures to enhance the accuracy and reliability of research findings. This multi-modal approach could provide a more nuanced understanding of treatment effects and should be considered a focal point for upcoming research endeavors.

Apart from the experimental design limitations, another critical impediment to the broader application of AAAT in the context of ASD is the paucity of research examining the underlying mechanisms of its effectiveness (70). Current hypotheses about the mechanisms through which AAAT affects ASD patients focus on five principal domains: (1) AAAT effectively captures the attention of ASD children, filtering out irrelevant external stimuli and thereby enabling longer periods of focus on environmental demands, which enhances social engagement (8, 41); (2) AAAT stimulates the vestibular and proprioceptive sensory systems in ASD patients, facilitating better body awareness, which in turn leads to improvements in sensory integration and bodily control (45); (3) the act of tactile interaction with animals allows ASD patients to establish a healthy emotional feedback loop, consequently alleviating stress levels (34, 47, 71); (4) physical exercise plays a crucial role in ASD treatment. The allure of animals encourages various forms of physical engagement in ASD patients, which then promotes improved behavioral and motor skills (8, 63); (5) during the AAAT process, therapists often issue commands to animals while encouraging ASD patients to interact with them, thereby enhancing the communicative abilities of the patients (32). Overall, the efficacy of AAAT for treating ASD can be explained through a biopsychosocial model, where biological, psychological, and social factors interact to produce beneficial therapeutic outcomes. From a physiological perspective, petting animals can increase oxytocin levels, reduce cortisol levels, and improve heart rate variability (HRV) in patients. These changes indicate that AAT programs can effectively regulate stress and anxiety, promoting social interaction and emotional bonding. Psychologically, animals provide unconditional acceptance and companionship, meeting the emotional needs of individuals with ASD and serving as a bridge for communication with therapists and caregivers. The multisensory experiences provided by interacting with animals help improve sensory integration, reduce problematic behaviors, and enhance treatment motivation and adherence. Therefore, AAAT programs combine the physiological mechanisms of stress reduction and oxytocin release with the psychological benefits of emotional support, sensory integration, and behavioral reinforcement to aid in the treatment of individuals with ASD. While these hypotheses offer a preliminary explanation for the observed benefits of AAAT, they fall short of elucidating which specific mechanisms play a pivotal role in this therapeutic process. Therefore, future research should center its focus on uncovering these underlying mechanisms. We contend that a concentrated investigation into the mechanisms of AAAT will substantially advance the field, providing the much-needed empirical basis for more targeted and effective interventions in the treatment of ASD.

Beyond the core symptoms and treatment methodologies, there are critical aspects of ASD and AAAT that warrant academic scrutiny. For instance, the challenging behaviors manifested by ASD patients, such as hyperactivity, aggression, repetitive actions, and attentional difficulties, have been noted to exert a negative impact on the well-being of caregivers, leading to outcomes like emotional exhaustion and depersonalization (28). Surprisingly, scant research has explored whether these behaviors similarly affect the animals involved in the therapeutic process.

Another significant observation relates to sex disparities among study participants. Out of 1,212 individuals included in the existing literature, only 328 are female. Although the general male-to-female ratio for ASD is roughly 4:1 (1), the relative underrepresentation of female participants in these studies is concerning. Female with ASD often exhibit better ‘masking’ abilities, complicating their diagnosis and, subsequently, their inclusion in research samples. Therefore, future studies must aim to include a more proportionate number of female participants to capture a more comprehensive picture of ASD and its treatment avenues. Regarding the duration of AAAT interventions, no conclusive research has ascertained the optimal treatment timeframe. However, a study by Llambias et al. (8) found that social participation skills in ASD children showed significant improvement in the early stages of AAI but plateaued over time, possibly due to a ‘ceiling effect.’ This observation necessitates further validation in subsequent research.

Moreover, abundant evidence confirms that AAAT can alleviate stress in ASD patients (49). Despite stress not being a core symptom of ASD, it remains a critical variable due to its capacity to precipitate a myriad of severe health concerns. And stress also significantly affects individuals’ quality of life (72). High-functioning ASD individuals, who often lack adequate social support, may resort to “masking” or concealing their symptoms to gain social acceptance. Such masking behaviors can perpetuate a chronic state of heightened stress, culminating in exhaustion, social withdrawal, depression, or even suicidal tendencies (66, 73). We argue that greater scholarly attention should be directed toward the stress levels in ASD patients, and perhaps also in their caregivers, to establish a more nuanced understanding of ASD pathophysiology. When assessing the efficacy of any intervention, including AAI, changes in stress levels among ASD patients should be incorporated as a key evaluation metric.

In summary, based on current evidence, we assert that AAI has the potential to effectively ameliorate core symptoms and alleviate anxiety in patients with ASD. However, it is important to acknowledge the existing gaps in high-quality research within this domain. The success of AAI as a viable alternative or adjunctive treatment method extends beyond the mere involvement of animals. The strategic selection of specific techniques and interventions, tailored to particular contexts, is essential. More precisely, AAAT programs should be designed with structured content, employing well-trained animals under the guidance of qualified animal handlers. Additionally, the involvement of skilled volunteers and safety personnel is crucial. The programs should also incorporate reasonable scheduling and the use of sensory aids, such as visual and auditory tools, to facilitate treatment. Moreover, a multi-disciplinary approach, engaging expertise from psychiatry, psychology, speech therapy, occupational therapy, and nursing, should be employed to create a comprehensive treatment plan for the ASD population. Given these considerations, while AAI offers promising prospects for improving the lives of those affected by ASD, the existing body of research calls for further empirical rigor. A multi-faceted, methodologically robust framework is necessary to substantiate AAAT’s efficacy and provide clear guidelines for its systematic application in ASD treatment.

## Data availability statement

The original contributions presented in the study are included in the article/Supplementary material, further inquiries can be directed to the corresponding authors.

## Author contributions

NX: Conceptualization, Data curation, Formal analysis, Funding acquisition, Investigation, Methodology, Project administration, Resources, Software, Visualization, Writing – original draft, Writing – review & editing. VB: Data curation, Resources, Writing – original draft. DY: Funding acquisition, Validation, Writing – original draft. XH: Funding acquisition, Validation, Writing – original draft. LZ: Data curation, Project administration, Writing – original draft. SK: Supervision, Validation, Writing – review & editing. MB: Supervision, Validation, Writing – review & editing. IT: Supervision, Validation, Writing – review & editing. VC: Supervision, Validation, Writing – review & editing.

## Footnotes

^1^ https://scholar.google.com/

## Glossary

AAA: Animal-Assisted Activities
AAAT: Animal-assisted activities and therapies
AAPT: Animal-Assisted Play Therapy
AAT: Animal Assisted Therapy
ABAS: Adaptive Behavior Assessment System
ABC-C: Aberrant Behavior Checklist–Community
ABLLS-R: The Assessment of Basic Language and Learning Skills-Revised
ADHD: Attention deficit hyperactivity disorder
ADI-R: Autistic disorder interview-revised
ADOS: Autism Diagnostic Observation Schedule
AHA: American Hippotherapy Association
AOTA: American Occupational Therapy Association
ASD: Autism Spectrum Disorders
ASQ: Autism spectrum quotient
BA: Barn Activities
BDCAS-P: Behavioral disorders of children with autism spectrum (parent form)
BOT: Bruininks-Oseretsky Test
CABTA: Children’s Attitudes and Behaviors Towards Animals
CAB-T: Clinical assessment battery teacher rating form
CAOT: Canine Assisted Occupational Therapy
CARS: Childhood Autism Rating Scale
COPM: Canadian Occupational Performance Measure
DAPAI: Dog-Assisted Physical Activity Intervention
DAT: Dog Assisted Therapy
DCDQ’07: Developmental Coordination Disorder Questionnaire, as revised in 2007
DSDFSHN: Dutch service dog foundation Stichting Hulphond Nederland
DSM: Diagnostic and Statistical Manual of Mental Disorders
DST: Dolphin Service Therapy
EAOT: Equine-assisted occupational therapy
EAT: Equine-assisted therapy
GARS: Gilliam Autism Rating Scale
GAS: Goal Attainment Scaling
GGZ-OB: GGZ Oost Brabant
ICD: International Classification of Diseases
ID: Intellectual Disability
IEMS: Interaction Emotions Motor Skills
IETC①: International Equestrian Training Center, China
OTEE: Occupational therapy in an equine environment
PATH: Professional Association of Therapeutic Horsemanship International
PEDro: Physiotherapy Evidence Database scale
PEDI-CAT ASD: Pediatric Evaluation of Disability Inventory Computer Adaptive Test, Autism Spectrum Disorder Module
PDDBI: Pervasive Developmental Disorder Behavior Inventory
PER-R: Psychoeducational Profile Revised
PSI-SF: Parenting Stress Index–Short Form
PSS: Perceived Stress Scale
RA: Regular Activities
REVT: Receptive and Expressive Vocabulary Test
ROBINS-I: Risk Of Bias In Non-randomized Studies—of Interventions
SALT: Systematic Analysis of Language Transcripts
SCL-90-R: Symptom Checklist-90-Revised
SCQ: Social Communication Questionnaire
SIPT: Sensory Integration and Praxis Test
SP: Sensory Profile
SRS: Social Responsiveness Scale
SSIS-RS: Social Skills Improvement System Ratting Scales
SSSS: Stone’s social skills Scale
TGMD-3: Test of Gross Motor Development-Third Edition
THR: Therapeutic Horseback Riding
UCOT: Usual Care Occupational Therapy
UQHREC: University of Queensland Human Research Ethic Committee
UQPCAEC: University of Queensland Production and Companion Animals Ethics Committee
VABS: Vineland Adaptive Behavioral Scales
WAIS: Wechsler Adult Intelligence Scale III/IV.
