# Supplementary material for: Effectiveness of animal-assisted activities and therapies for autism spectrum disorder: a systematic review and meta-analysis
Source: Front Vet Sci. 2024 Jun 3;11:1403527. doi: 10.3389/fvets.2024.1403527 (PMC11184216; doi:10.3389/fvets.2024.1403527)
Supplement: Supplementary file 1 [file Table_1.docx]

Supplementary Table 1. Experimental characteristics

| First Author, Publish year | Study_ID | Type of intervention | Type of animal | Type of trial | Description of intervention | Other therapy | Control Group | Setting of country | Duration (days) | Session time (min) | Total time (min) | Intervention provider accreditation |
| --- | --- | --- | --- | --- | --- | --- | --- | --- | --- | --- | --- | --- |
| B Caitlin Peters, 2022 | 1 | OTEE | Horse | RCTs | (1) greetings (2) activities with horses (3) goodbyes, and caregiver debrief | (-) | OT | USA | 10 | 60 | 600 | AOTA, AHA, PATH |
| Rezapour-Nasrabad, R.R， 2022 | 2 | Hippotherapy | Horse | Pre-post | (1) sit properly on the horse (2) ride a horse (3) visit the stables (4) goodbye | (-) | n/a | Iran | 10 | 45 - 60 | 450 - 600 | UAA |
| Zhao, M, 2022 | 3 | THR | Horse | RCTs | (1) Warm-up exercise (2) Riding and horsemanship skills instruction (3) THR exercises and activities (4) Cool-down and reward activity | (-) | Waitlist | China | 24 | 60 | 1440 | (-) |
| Abadi, M.R.H., 2022, Anthrozoos | 4 | DAPAI | Dog | Crossover design | Therapy dog handlers guided the participants’ interactions | (-) | n/a | USA | 4 | 60 | 240 | IHO |
| Mengxian Zhao, 2021 | 5 | THR | Horse | RCTs | (1) Warm activities (2) Riding skills and horsemanship skills instruction (3) THR exercises and activities (4) Cool down and reward activities | (-) | RA | China | 32 | 60 | 1920 | IETC① |
| Leonardo Zoccante, 2021 | 6 | EAT | Horse | Pre-post | Moving from school to vacation and vice versa | (-) | n/a | Italy | 20 | 45 | 900 | University Hospital of Verona |
| Carolien Wijker, 2021 | 7 | AAT | Dog | RCTs | Executing the exercises together with the therapy dog | (-) | Waitlist | Netherlands | 10 | 60 | 600 | CMO region Arnhem-Nijmegen |
| Peters, B.C., 2021 | 8 | OTEE | Horse | RCTs | (1) Use horses to optimize attention and engagement (2) Design therapeutic activities with horses (3) Provide positive reinforcement for goal behaviors (4) Scaffold goal performance using behavioral techniques (5) Arrange environment to best support goal performance | (-) | OT | USA | 10 | 60 | 600 | PATH, Colorado State University’s IRB |
| Hernández-Espeso, 2021 | 9 | DAI | Dolphin | RCTs | (1) Put on their wetsuits and shoes (2) Introduce the session structure to the child and remind him/her of safety rules  (3) Greeting the dolphin trainer.  (4) Feed the dolphin and make the initial greeting (5) Activities on the edge of the pool with the dolphin  (6) Activities in the water with the dolphin | (-) | TWD | Spain | 18 | 45 | 810 | (-) |
| Jessica Hill, 2020 | 10 | CAOT | Horse | RCTs | (1) Introduce therapy dog (2) Beginning intervention, various activities (3) Ending the session (4) Follow-up session | Y | UCOT | Australia | 9 | 60 | 540 | UQHREC UQPCAEC |
| Carolien Wijker, 2020 | 11 | DAT | Dog | RCTs | The program had a structured protocol and consisted of 10 weekly one-on-one sessions of 60 min per session. A therapy dog was involved during all the therapy sessions | Y | Waitlist | Netherlands | 10 | 60 | 600 | DSDFSHN GGZ-OB |
| B Caitlin Peters, 2020 | 12 | OTEE | Horse | Multiple baseline SCED | (1) manipulation of equine movement to promote functional outcomes (2) Activities to address individual goals (3) Facilitation of social interaction (4) Positive reinforcement of communication | (-) | n/a | USA | 10 | 45 - 60 | 450 - 600 | PATH |
| Adriana Ávila-Álvarez, 2020 | 13 | AAI | Dog | Quasi-experiment | 5 Groups: Group 1 - activities involving getting to know the animal Group 2 - activities involving interaction with the dog Group 3 - activities involving caring for the animal Group 4 - distractions involving playing with the dog Group 5 - features of intervention with therapy dogs | (-) | (-) | Spanish | 24 | 20-30 | 480-720 | (-) |
| Isabel Morales-Moreno, 2020 | 14 | AAT | Dog | Quasi-experiment | (1) stimulation of language (2) learning of basic activities of daily life (3) relaxation and concentration (4) promote and tailor social relationships (5) promote physical benefits | (-) | RA | Spanish | 12 | 60 | 720 | (-) |
| Portela-Pino, I.,  2020 | 15 | EAT | Horse | Pre-post | (-) | (-) | (-) | Spanish | 32 | 60 | 1920 | (-) |
| Kalmbach, D.， 2020 | 16 | OTEE | Horse | Pre-post | (1) pre-mounted segment (2) mounted segment (3) post-mounted segment | (-) | (-) | USA | 10 | 45 - 60 | 450 - 600 | AOTA |
| Ozyurt, Gonca., 2020 | 17 | EAA | Horse | RCTs | (1) preparation, warm-up (2) grooming and feeding (3) mounting and dismounting  (4) horsemanship activities (5) finishing | (-) | RT | Turkey | 8 | 30 | 240 | PATH |
| Monique M Germone, 2019 | 18 | AAA | Dog | crossover study design | Quiet play (baseline), 10-min experimental or control condition separated by a 2-day wash-out period. | (-) | Control Group | USA | (-) | 30 | (-) | (-) |
| Ana L L Michelotto, 2019 | 19 | AAA | Dog | Pre-post | (-) | (-) | (-) | Brazil | 10 | 30 | 300 | (-) |
| Kwon, S., 2019 | 20 | THR | Horse | RCTs | (1) Stretching Exercises (2) Riding skills and riding (3) Interact with horse (such as brushing, feeding, putting stickers on their horses) | (-) | CT | Korea | 8 | 30 | 240 | (-) |
| Robin L Gabriels, 2018 | 21 | THR | Horse | RCTs | Therapeutic riding skills (e.g., mounting, halting, steering, turning, and trotting) and 2) horsemanship skills (e.g., how to lead and care for their horse). Lessons followed a consistent routine presented as a picture schedule: 1) put on riding helmet, 2) wait on the bench, 3) mount horse, 4) riding activities, 5) dismount horse, 6) groom horse, and 7) put away equipment | (-) | BA | USA | 10 | 45 | 450 | PATH |
| Pan, Z., 2018 | 22 | THR | Horse | RCTs | (1) Saliva collection (2) Sit with a volunteer (3) Start group (4) Review group schedule (5) Warm up exercises (6) Lesson & activity (7) Cool down exercises (8) THR group dismount &thank horses -all groups thank volunteers (9) Drawing activity at table (20 min) (10) Saliva collection | (-) | BA | USA | (-) | 45 | (-) | PATH |
| Tan, V. X., 2018 | 23 | EAI | Horse | Pre-post | (-) | ST SST | n/a | Australia | (-) | (-) | (-) | (-) |
| Androulla Harris, 2017 | 24 | AAI | Horse | RCTs | (1) Preparing and Mounting (2) Riding Skills and Exercises (3) Stretching Exercises (4) Thanks to instructor and horse | (-) | Waitlist | UK | 5 - 7 | 45 | 225 - 315 | BHS |
| Cecilia Llambias, 2016 | 25 | EAOT | Horse | Pre-post | All sessions (baseline, intervention, follow-up) were 45–60 min long. Children spent >20 min in gross motor (GM), or physical, activities and 20 min in fine motor (FM), or cognitive, activities in all sessions in all phases. Activities off (considered FM) and on (considered GM) the horse during intervention. | Y | N | Canada | 8 | 45 - 60 | 360 - 480 | AHA, PATH |
| Marta Borgi, 2016 | 26 | EAT | Horse | RCTs | (1) Grooming and hand walking the horse (2) Horseback riding (3) closure, feeding the horse and saying goodbye to the horse ad to the group | (-) | Waitlist | Italy | 25 | 60-70 | 1500 - 1750 | IFES |
| Sophie Anderson, 2016 | 27 | EAA | Horse | Pre-post | (1) Heathy and safety briefing (2) Parents completed self-assessments and interviews (3) Horsemanship activities, including grooming, leading and mucking out (4) Therapeutic Riding | (-) | n/a | UK | 5 | 180 | 900 | BHS |
| Robin L Gabriels， 2015 | 28 | EAT, THR | Horse | RCTs | (1) Warm up (2) Therapeutic Riding skills (mounting, halting, steering, running, trotting) (3) Horsemanship skills (How to lead and care for horse) (4) Cool down | Psychotropic medicine | BA | USA | 10 | 45 | 450 | PATH |
| H Steiner, 2015 | 29 | THR | Horse | RCTs | (1) Warming up exercise of stretching on horseback while horses were not moving (2) Horseback riding | (-) | RT | Hungary | 4 | 30 | 120 | (-) |
| Beth A Lanning, 2014 | 30 | EAA | Horse | RCTs | (1) Basic safety lessons (2) Grooming lessons (3) Riding activities | (-) | Non-equine intervention | USA | 12 | 60 | 720 | PATH |
| Marguerite E O'Haire, 2014 | 31 | AAA | Guinea pig | Control-to-intervention design | Holding, Feeding, Floor time, Visual art, Health monitoring, Construction, Grooming & Cage Cleaning | (-) | Waitlist VS Non-waitlist | Australia | 8 | 40 | 320 | (-) |
| Margo B Holm, 2014 | 32 | THR | Horse | Pre-post | (1) Grooming, emphasizing touch, naming of parts  and following instructor (2) Riding session | (-) | n/a | USA | 4 | 30 - 45 | 120 - 180 | NARHA |
| Fung, S.-C., 2014 | 33 | AAPT | Dog | RCTs | Focused on the child’s interaction with the dog or the doll. Focused on interaction between the child, the dog, or the doll and the therapist. Focused on the child-therapist interaction in the presence of the dog or the doll. Focused on the child-therapist interaction with the fade-out of the dog or the doll. | (-) | Waitlist | China | 14 | 20 | 280 | (-) |
| Heather F Ajzenman, 2013 | 34 | EAOT | Horse | Pre-post | Forward sitting (astride), prone, supine, backward astride, side sit, kneeling, quadruped, and standing. | (-) | (-) | USA | 12 | 45 | 540 | PATH |
| Sandra C Ward, 2013 | 35 | TR | Horse | Pre-post | (1) Orientation (2) Mounting and Riding (3) Riding Skills  (4) Closure | OT | n/a | USA | 14 | 40 - 45 | 560 - 630 | PATH |
| Ghorban, Hemati.， 2013 | 36 | THR | Horse | Pre-post | 1) Familiarity Stage (2) Practices (3) Riding Skills (4) End of Riding Stage | (-) | n/a | Iran | 8 | 45 | 360 | (-) |
| Jenkins, Sarah R., 2013 | 37 | THR | Horse | Pre-post | Creating lesson plans based on each rider’s skill level and acquisition of target horsemanship skills | (-) | Waitlist | USA | 9 | 60 | 540 | PATH |
| Emílio Salgueiro, 2012 | 38 | DAI | Dolphin | Pre-post | Swim-with-the-dolphins | (-) | n/a | Portugal | 12 | (-) | (-) | (-) |
| Tabares, C., 2012 | 39 | Hippotherapy | Horse | Pre-post | (1) make contact with the animal (2) mounts the horse (3) exercise ring (4) dismount the horses  (5) says goodbye | (-) | n/a | Spanish | 4 | (-) | (-) | AZE |
| MdYusof, 2012 | 40 | DST | Dolphin | Pre-post | Swimming with, touching or taking care of dolphins. Based on a structured program designed to meet the needs of the individual concerned | (-) | (-) | Singapore | (-) | (-) | (-) | (-) |
| Gabriels, Robin L., 2012 | 41 | THR | Horse | Pre-post | (1) put riding helmet on (2) sit and wait on the bench (3) mount horse (4) THR activities (5) dismount horse (6) groom horse (7) put away equipment | Psychoactive medications | Waitlist | USA | 10 | 60 | 600 | PATH |
| Janet K Kern, 2011 | 42 | EAA | Horse | Pre-post | (1) before beginning a 3-to-6 month waiting period (2) before starting the riding treatment  (3) after 3 months  (4) 6 months of riding | (-) | n/a | USA | 24 | 60 | 1440 | (-) |
| Robert Viau, 2010 | 43 | DAT | Dog | Pre-post | Interact with the dog | (-) | n/a | Canada | (-) | (-) | (-) | MIRA |
| Taylor, Renee R., 2009 | 44 | Hippotherapy | Horse | Pre-post | (1) donning of the helmet, preparation to mount (2) on the horse, and time for dismount | (-) | n/a | USA | 16 | 45 | 720 | (-) |
| Bass, M. M., 2009 | 45 | THR | Horse | RCTs | (1) Mounting and dismounting (2) Warm-up exercises to stretch their bodies (3) Riding skills (4) Mounted Games (5) Horsemanship Activities | Y | Waitlist | USA | 12 | 60 | 720 | (-) |

Table 2 is sorted by chronology and abbreviation in alphabetical order.

Abbreviation: BA, Barn Activities; PATH, Professional Association of Therapeutic Horsemanship International; IETC①, International Equestrian Training Center, China; UCOT, Usual Care Occupational Therapy; UQHREC, University of Queensland Human Research Ethic Committee; UQPCAEC, University of Queensland Production and Companion Animals Ethics Committee; AHA, American Hippotherapy Association; EAOT, Equine-assisted occupational therapy; DSDFSHN, Dutch service dog foundation Stichting Hulphond Nederland; GGZ-OB, GGZ Oost Brabant; AOTA, American Occupational Therapy Association; UAA, Urmia Autism Association; IETC, International Equestrian Training Center; DAPAI, Dog-Assisted Physical Activity Intervention; IHO, International humanitarian organization; SCED, single-case experimental design; AZE, Association of Zootherapy of Extremadura; DAI, Dolphins assisted intervention; DST, Dolphin Service Therapy; NARHA, the North American Riding for the Handicapped Association; RT, Regular therapy; BHS, British Horse Society; IFES, Italian Federation of Equestrian Sports; METP, motorized elephant-assisted therapy program;
