# Supplementary material for: Effectiveness of animal-assisted activities and therapies for autism spectrum disorder: a systematic review and meta-analysis
Source: Front Vet Sci. 2024 Jun 3;11:1403527. doi: 10.3389/fvets.2024.1403527 (PMC11184216; doi:10.3389/fvets.2024.1403527)
Supplement: Supplementary file 2 [file Table_2.docx]

Supplementary Table 2. Outcome characteristics

| First Author, Publish year | Measured outcome | Type of  effect (B/W) | Reported effect (p < 0.05) | | | | | | | Estimate effect (95%CI) (p < 0.05) | |  |
| --- | --- | --- | --- | --- | --- | --- | --- | --- | --- | --- | --- | --- |
|  |  |  | Experimental group | | | Control group | | | |  |  |  |
|  |  |  | Baseline (Mean ± SD) | After treatment (Mean ± SD) | Change | | Baseline (Mean ± SD) | After treatment (Mean ± SD) | Change | |  | |
| B Caitlin Peters, 2022 | Self-regulation, social communication, and social play | B/W | GAS: Primary goal: -2.00 Average of all goals: -2.00  ABC: Irritability: 14.65 ± 6.99 SRS-2: Social motivation: 69.85 ± 9.39 | GAS: Primary goal: 0.75 ± 1.45 Average of all goals: 0.39 ± 1.13 ABC: Irritability: 12.00 ± 5.89 SRS-2: Social motivation: 66.75 ± 12.39 | GAS: Primary goal: 2.75 Average of all goals: 2.39 ABC: Irritability: -2.65 ± 5.36 SRS-2: Social motivation: -3.1 ± 6.05 | | GAS: Primary goal: -2.00 Average of all goals: -2.00 ABC: Irritability: 17.68 ± 4.69 SRS-2: Social motivation: 74.67 ± 8.20 | GAS: Primary goal: 0.00 ± 1.22 Average of all goals: -0.48 ± 1.03 ABC: Irritability: 15.53 ± 6.84 SRS-2: Social motivation: 71.00 ± 7.86 | GAS: Primary goal: 2.00 Average of all goals: 1.52 ABC: Irritability: -2.15 ± 4.72 SRS-2: Social motivation: -3.67 ± 1.87 | | (-) | |
| Rezapour-Nasrabad, R.R， 2022 | Behavioral and executive function | W | BDCAS-P: Initiation: 19.75 ± 1.38 Active memory: 32.37 ± 2.19 planning: 31.62 ± 1.59 Organizing tools: 22.37 ± 1.3 Metacognition index: 130.12 ± 4.79 Inhibition: 28.38 ± 2.06 The transfer: 30.87 ± 1.64 Emotional control: 37.12 ± 1.45 Behavior regulation index: 96.37 ± 2.92 Executive function: 226.5 ± 5.5 | BDCAS-P: Initiation: 15.87 ± 1.95 Active memory: 22.25 ± 3.77 planning: 25.37 ± 2.5 Organizing tools: 16.75 ± 3.77 Metacognition index: 101 ± 6.11 Inhibition: 18.62 ± 1.3 The transfer: 25.62 ± 2.77 Emotional control: 20.37 ± 3.42 Behavior regulation index: 64.62 ± 3.88 Executive function: 165.62 ± 8.1 | (-) | | (-) | (-) | (-) | | (-) | |
| Zhao, M, 2022 | Motor skills | B/W | TGMD-3:  Run: 3.58 ± 0.58 Gallop: 2.73 ± 0.53 Total: 14.85 ± 2.41 | TGMD-3:  Run: 4.15 ± 0.61 Gallop: 3.19 ± 0.49 Total: 16.04 ± 2.32 | (-) | | TGMD-3:  Run: 3.37 ± 0.63 Gallop: 2.78 ± 0.58 Total: 14.56 ± 2.38 | TGMD-3:  Run: 3.48 ± 0.64 Gallop: 2.85 ± 0.53 Total: 14.81 ± 2.35 | (-) | | (-) | |
| Abadi, M.R.H., 2022 | PA | W | (-) | (-) | (-) | | (-) | (-) | (-) | | Light PA: 3.5 (1.2, 5.8), p = 0.005 Sedentary Time: –2.4 (–4.3, –0.1), p = 0.04 | |
| Mengxian Zhao, 2021 | Behavior changes in social; Communication skills; | B/W | SISS: Social skills: 44.68 ± 7.48 Sub-domains: Communication: 6.71 ± 1.77 Cooperation: 7.55 ± 1.77 Assertion: 4.90 ± 1.58 Responsibility: 6.23 ± 1.23 Empathy: 5.42 ± 1.29 Engagement: 6.65 ± 1.45 Self-control: 7.23 ± 1.73 ABLLS-R: Social interaction scores: 24.03 ± 3.38 | SISS: Social skills: 50.87 ± 6.47 Sub-domains: Communication: 8.48 ± 1.86 Cooperation: 8.16 ± 1.73 Assertion: 5.71 ± 1.47 Responsibility: 7.00 ± 1.24 Empathy: 5.90 ± 1.27 Engagement: 7.68 ± 1.51 Self-control: 7.94 ± 1.55 ABLLS-R: Social interaction scores: 33.84 ± 4.00 | (-) | | SISS: Social skills: 44.27 ± 4.31 Sub-domains: Communication: 7.03 ± 1.54 Cooperation: 7.50 ± 1.41 Assertion: 4.63 ± 1.10 Responsibility: 5.87 ± 1.01 Empathy: 5.70 ± 1.02 Engagement: 6.47 ± 1.14 Self-control: 7.07 ± 1.53 ABLLS-R: Social interaction scores: 24.13 ± 3.59 | SISS: Social skills: 45.43 ± 5.08 Sub-domains: Communication: 7.27 ± 1.46 Cooperation: 7.63 ± 1.22 Assertion: 5.07 ± 1.39 Responsibility: 6.13 ± 1.17 Empathy: 5.53 ± 1.17 Engagement: 7.03 ± 1.19 Self-control: 6.77 ± 1.55 ABLLS-R: Social interaction scores: 25.87 ± 3.05 | (-) | | (-) | |
| Leonardo Zoccante, 2021 | Adaptive Behavior and Motor Function | W | Vineland-Ⅱ:  Communication: 48.1, SE[6.5] Socialization: 55.5, SE[4.9] Daily living skills: 60.5, SE[5.0] Motor skills: 66.9, SE[8.3] PSI-SF: Total Score: 86.4, SE[4.3] Parental distress: 30.5, SE[2.4] PCDI: 25.7, SE[1.4] Difficult Child: 29.6, SE[1.7] DCDQ'07: Total Score: 37.5, SE[2.4] | Vineland-Ⅱ:  Communication: 57.5, SE[6.4] Socialization: 63.0, SE[5.4] Daily living skills: 72.5, SE[5.2] Motor skills: 83.6, SE[6.9] PSI-SF: Total Score: 87.7, SE[6.0] Parental distress: 30.1, SE[2.6] PCDI: 26.2, SE[1.9] Difficult Child: 32.4, SE[1.9] DCDQ'07: Total Score: 40.2, SE[2.1] | (-) | | (-) | (-) | (-) | | Vineland-2: Communication: 9.4 (4.8 to 14), p < 0.001 Daily Living Skills: 12 (8.3 to 15.7), p < 0.001 Socialization: 7.47 (2.1 to 12.8), p = 0.01 Motor skills: 16.73 (6.1 to 27.4), p < 0.001 DCDQ’07:  3.6 (1 to 6.3), p = 0.01 PSI-SF:  Difficult Child: 2.7 (0.2 to 5.2), p = 0.03 IEMS:  Social interaction: 0.32 (0.2 to 0.4), p < 0.001 Emotions-relation: 0.31 (0.2 to 0.4), p < 0.001 Behavior: 0.27 (0.2 to 0.3), p < 0.001 Gross motor skills: 0.25 (0.2 to 0.3), p < 0.001 Fine motor skills: 0.19 (0.1 to 0.3), p < 0.001 | |
| Carolien Wijker, 2021 | Stress | B/W | Cortisol: 10.04 ± 3.14 | Cortisol: 8.74 ± 3.14 | (-) | | (-) | (-) | (-) | | Cortisol: -0.41 (-0.72 to -0.11), p = 0.01 | |
| Peters, B.C., 2021 | (-) | B/W | (-) | (-) | (-) | | (-) | (-) | (-) | | (-) | |
| Hernández-Espeso, 2021 | Social and Communication Skills | B/W | ADOS - G:   Language and communication domain: 5.87 ± 2.39  Frequency of vocalizations: 1.39 ± 0.72  Gestures: 1.45 ± 0.67  Social Interaction Domain: 9.14 ± 3.03 | ADOS - G:  Language and communication domain: 4.73 ± 2.25  Frequency of vocalizations: 1.18 ± 0.59  Gestures: 1.14 ± 0.64  Social Interaction Domain: 8.82 ± 3.18 | ADOS - G:  Language and communication domain: 1.14 Frequency of vocalizations: 0.21  Gestures: 0.31 Social Interaction Domain: 0.32 | | ADOS - G:  Language and communication domain: 5.12 ± 1.40  Frequency of vocalizations: 1.53 ± 0.62 Gestures: 1.29 ± 0.69  Social Interaction Domain: 10.12 ± 3.35 | ADOS - G:  Language and communication domain: 4.53 ± 1.33  Frequency of vocalizations: 1.53 ± 0.62  Gestures: 1.35 ± 0.70  Social Interaction Domain: 9.94 ± 4.12 | ADOS - G:  Language and communication domain: 0.59  Frequency of vocalizations: 0  Gestures: -0.06  Social Interaction Domain: 0.18 | | Pre- to posttest (Z = –3.86, p < 0.001) Frequency of the vocalizations (Z = –2.24, p < 0.05). Comprehension skills in both groups (Z = –4.24, p < 0.001) Expression skills (Z = –3.39, p < 0.001) | |
| Jessica Hill, 2020 | On-task behavior | B/W | On-task behavior statistics:  First session: 92.3232 ± 5.70509  Last session: 91.1250 ± 8.15559  Sessions combined: 92.3232 ± 5.70509 | (-) | (-) | | On-task behavior statistics:  First session: 89.7659 ± 10.24441  Last session: 85.0836 ± 17.13939  Sessions combined: 89.7659 ± 10.24441 | (-) | (-) | | An ICC value of 0.957 was obtained (p < 0.01; 95% CI 0.921 – 0.976) | |
| Carolien Wijker, 2020 | Stress Self-esteem Social responsiveness | B/W | PSS: IQ, WAIS III/IV: 102.1 ± 13.7  Stress, PSS: 22.1 ± 6.2 SCL-90-R: 201.7 ± 56.8 Self-esteem, RSES: 24.1 ± 5.2 Social responsiveness, SRS-A : 92.4 ± 25.6  Social responsiveness (I), SRS-A (I) : 83.9 ± 26.1 | (-) | (-) | | (-) | (-) | (-) | | PSS: -3.3 (-6.1 to -0.5), p = 0.02; SRS: -11.9 (-20.3 to -3.5), p = 0.01; Subscales of SCL-90-R, Agoraphobia: -1.9 (-3.4 to -0.36), p = 0.016; Subscales of SRS-A, Social awareness: -3.4 (-6.2 to -0.6), p = 0.019; Social communication: -5.5 (-9.1 to -1.9), p = 0.03) | |
| B Caitlin Peters, 2020 | Performance goals, behavior, and social functioning | W | ABC-C: Irritability: 20.42 Hyperactivity: 24.75  Social motivation: 14  Social communication: 40 | ABC-C: Irritability: 12.28  Hyperactivity: 17.55  Social motivation: 12  Social communication: 38 | ABC-C: Irritability: 8.14 Hyperactivity: 7.2  Social motivation: 2  Social communication: 2 | | (-) | (-) | (-) | | ABC-C: social motivation: p = 0.04 social communication: z = 0.00, p = 0.04 | |
| Adriana Ávila-Álvarez, 2020 | Emotional well-being | W | Median: (Q1–Q3)  Happy: 4 (3–5) Lonely: 5 (4–5) Scared: 4 (3–5) Relaxed: 3 (3–5) Total score: 15 (13–18) | Median: (Q1–Q3)  Happy: 5 (5–5) Lonely:5 (5–5) Scared: 5 (5–5) Relaxed: 5 (5–5) Total score: 20 (18–20) | Median (Q1–Q3) Happy: -1  Lonely: 0 Scared: -1 Relaxed: -2 Total score: -5 | | (-) | (-) | (-) | | p < 0.001 | |
| Isabel Morales-Moreno, 2020 | Language, conversation, and stereotypes | B/W | (-) | (-) | (-) | | (-) | (-) | (-) | | (-) | |
| Portela-Pino, I.,  2020 | Motor development, social communication | W | Relaxation :  Dynamic coordination: 1.8  Motor dissociation: 1.8 Visuomotor coordination: 1.2  Spatial orientation: 1.4  Time structure: 1.6  Postural Attitudes:  Tonic-postural control: 1.4  Balance: 1.8 Lateralization: 1  Touch and contact:  Visual contact: 1.4  Body gestures: 1 Physical contact: 1.6  Relation with age pairs: 1  Learning dexterities:  Actions asking about or showing objects of interest: 1  Participation in social games during therapy: 1  Acknowledgement and verbal or body communication with the horse: 1   Ability to have a conversation with another person: 1  Emotios:  Intent on sharing the experience: 1.2  Intent on sharing personal achievement with others: 1.2  Emotional reciprocity: 1.2  Understanding the feelings of others: 1.8 | Relaxation:  Dynamic coordination: 2.6  Motor dissociation: 2.6  Visuomotor coordination: 2.6  Spatial orientation: 2.4 Time structure: 2.6  Postural Attitudes:  Tonic-postural control: 2.4  Balance: 2.4  Lateralization: 2  Touch and contact:  Visual contact: 2.8  Body gestures: 2.2 Physical contact: 2.8 Relation with age pairs: 1.8 Learning Dexterities: Actions asking about or showing objects of interest: 2.4  Participation in social games during therapy: 2  Acknowledgement and verbal or body communication with the horse: 2.8  Ability to have a conversation with another person: 1.2  Emotions:  Intent on sharing the experience: 2.6  Intent on sharing personal achievement with others: 2.4  Emotional reciprocity: 2.8  Understanding the feelings of others: 3 | (-) | | (-) | (-) | (-) | | (-) | |
| Kalmbach, D.， 2020 | Parental perspectives on the child's experience of occupational therapy | n/a | n/a | n/a | n/a | | n/a | n/a | n/a | | n/a | |
| Ozyurt, Gonca., 2020 | Social functioning, autistic behaviors, family functioning, and clinical severity | B/W | CGAS: 57 ± 9.24 FAD:  communication 2.5 ± 0.52 Role’s subscale 2.31 ± 0.59 Involvement 2.38 ± 0.58 Behavioral Control 2.23 ± 0.55 SCQ: 19.92 ± 4.12 BDI: 18.5 ± 6.31 | CGAS: 61.83 ± 11.47 FAD:  communication 2.2 ± 0.59 Role’s subscale 1.88 ± 0.38 Involvement 1.93 ± 0.59 Behavioral Control 1.93 ± 0.38 SCQ: 18.25 ± 3.70 BDI: 16.25 ± 5.46 | (-) | | (-) | FAD: Involvement 2.42 ± 0.56 Behavioral Control 2.35 ± 0.47 | (-) | | CGAS: p = 0.0004 FAD: communication p = 0.001 Responsiveness p > 0.05 Involvement p = 0.01 Behavioral Control p = 0.01 General Functions p > 0.05 SCQ: p = 0.002 BDI: p = 0.0001 | |
| Monique M Germone, 2019 | Communication | B/W | ABC-C: Irritability 25.9 ± 8.4  Lethargy 13.1 ± 8.9  Stereotypic 5.0 ± 4.1  Hyperactivity 28.0 ± 10.3  Inappropriate 4.8 ± 3.3 | (-) | (-) | | (-) | (-) | (-) | | (-) | |
| Ana L L Michelotto, 2019 | Behavior | W | Positive gestures or facial expression in dogs’ presence - 60.0%  Unquiet or excited in dogs’ presence - 33.3% | Positive gestures or facial expression in dogs’ presence - 80.0%  Unquiet or excited in dogs’ presence - 53.3% | Positive gestures or facial expression in dogs’ presence: -20.0%  Unquiet or excited in dogs’ presence: -20.3% | | (-) | (-) | (-) | | p = 0.010 | |
| Kwon, S., 2019 | Language Function; Cognitive Function; Intelligence and Achievement | W | REVT Reception: 17.44 ± 19.97 BSID-of Cognitive domain: IG: 130.38 ± 21.87 | REVT Reception: 20.11 ± 20.84 BSID-of Cognitive domain: 133.69 ± 23.29 | (-) | | REVT Reception: 13.82 ± 18.81 BSID-of Cognitive domain: 136.00 ± 19.51 | REVT Reception: 15.27 ± 18.12 BSID-of Cognitive domain: 138.33 ± 20.20 | (-) | | (-) | |
| Robin L Gabriels, 2018 | Irritability & Hyperactivity | B/W | Irritability: 15.86 ± 9.52  Hyperactivity: 20.75 ± 20.71 | Irritability: 9.00 ± 8.08  Hyperactivity: 13.28 ± 17.07 | Irritability: 6.86  Hyperactivity: 7.47 | | Irritability: 14.43 ± 8.69  Hyperactivity: 11.96 ± 9.29 | Irritability: 20.71 ± 20.75 Hyperactivity: 17.07 ± 13.28 | Irritability: -6.28  Hyperactivity: -5.11 | | Irritability: p < 0.02, after 6 months p =0.52 Hyperactivity: p = 0.08 after 6 months p = 0.2 | |
| Pan, Z., 2018 | Adaptive skills, aberrant and social behaviors | B/W | ABC-C:  Hyperactivity: 20.86 (12.13)  SRS: Social awareness: 15.43 (3.95)  Social Communication: 41.00 (9.33) | ABC-C:  Hyperactivity: 16.00 (8.64) SRS: Social awareness: 11.29 (1.38) Social Communication: 34.57 (3.95) | ABC-C: Hyperactivity: -4.86 (3.37) SRS: Social awareness: -4.14 (1.27) Social Communication: −6.43 (2.35) | | ABC-C:  Hyperactivity: 17.33 (4.46) SRS: Social awareness: 12.29 (2.56) Social Communication: 29.29 (9.83) | ABC-C:  Hyperactivity: 24.33 (6.02) SRS: Social awareness: 13.57 (4.12) Social Communication: 31.29 (10.98) | ABC-C: Hyperactivity: 7.28 (3.86) SRS: Social awareness: 1.29 (1.27) Social Communication: 2.00 (2.35) | | ABC-C:  Hyperactivity: p = 0.04 SRS: Social awareness: p = 0.01 Social Communication: p = 0.03 | |
| Tan, V. X., 2018 | Psychosocial outcomes | n/a | (-) | (-) | (-) | | (-) | (-) | (-) | | (-) | |
| Androulla Harris, 2017 | Irritability, Lethargy, Stereotypy, Hyperactivity, and Inappropriate Speech | B/W | CARS2 Score: 40.95 ± 6.07  ABC-C:  Hyperactivity: 26.30 ± 10.73  Irritability: 20.20 ± 8.78  Lethargy: 14.60 ± 3.86  Stereotypy: 10 ± 4.74  Inappropriate Speech: 3.40 ± 3.89 | CARS2 Score: 40.05 ± 5.57  ABC-C:  Hyperactivity: 22.30 ± 9.67  Irritability: 18.90 ± 7.58  Lethargy: 14.90 ± 3.78  Stereotypy: 10.50 ± 3.69  Inappropriate Speech: 3.60 ± 3.92 | CARS2 Score: -0.9  ABC-C:  Hyperactivity: -4  Irritability: -1.3  Lethargy: 0.3  Stereotypy: 0.5  Inappropriate Speech: 0.2 | | CARS2 Score: 42.61 ± 7.52  ABC-C:  Hyperactivity: 21 ± 11.07  Irritability: 22.50 ± 10.08  Lethargy: 14.43 ± 5.76  Stereotypy: 7.71 ± 6.09  Inappropriate Speech: 2.93 ± 3.56 | CARS2 Score: 42.61 ± 7.52  ABC-C:  Hyperactivity: 21 ± 11.07  Irritability: 22.50 ± 10.08  Lethargy: 14.14 ± 6.40  Stereotypy: 7.79 ± 6.22  Inappropriate Speech: 3.07 ± 3.56 | CARS2 Score: 0  ABC-C:  Hyperactivity: 0  Irritability: 0  Lethargy: - 0.3  Stereotypy: 0.08 Inappropriate Speech: 0.14 | | CARS2 Score - p: 0.013  Hyperactivity - p: 0.009 Lethargy - p: 0.227 | |
| Cecilia Llambias, 2016 | Behavioural Engagement | W | (-) | (-) | (-) | | (-) | (-) | (-) | | (-) | |
| Marta Borgi, 2016 | Communication, Daily Living Skills, Socialization, and Motor Skills | B/W | VABS: Socialization: 0.72 ± 0.22  Motor Skills: 0.28 ± 0.06  TOL: Planning time: -20.7 ± 6.6 | (-) | (-) | | VABS: Socialization: 0.23 ± 0.21  Motor Skills: -0.26 ± 0.20 TOL:  Planning time: -6.46 ± 5.2 | (-) | (-) | | (-) | |
| Sophie Anderson, 2016 | Communication Socialization Maladaptive behavior  Empathizing quotient  Systemizing quotient | W | ASQ: 62.13 ± 24.20 VABS:  Total: 210.13 ± 124.32  Communication: 44.20 ± 31.19  Socialization: 45.27 ± 23.21  Maladaptive behavior: 28.26 ± 12.82 Empathizing quotient: 14.86 ± 10.21 Systemizing quotient: 26.53 ± 14.20  EQ/SQ: 14.86 ± 16.73 | ASQ: 60.66 ± 24.19  VABS:  Total: 214.86 ± 121.63  Communication: 44.60 ± 30.39  Socialization: 45.07 ± 22.48  Maladaptive behavior: 26.73 ± 12.41  empathizing quotient: 6.20 ± 9.32  Systemizing quotient: 26.27 ± 13.72  EQ/SQ : 13.93 ± 14.99 | ASQ: 1.47  VABS:  Total: -4.73  Communication: -0.40  Socialization: 0.20  Maladaptive behavior: 1.53  Empathizing quotient: 8.66  Systemizing quotient: 0.26 EQ/SQ: 0.93 | | (-) | (-) | (-) | | VABS maladaptive behavior score [F(1,11) = 5.65, p = 0.037, gp 2 = 0.339]  EQ score [F(1,11) = 5.19, p = 0.04, gp 2 = 0.320] | |
| Robin L Gabriels， 2015 | Self-Regulation; Socialization; Communication; Adaptive, and motor behaviors; | B/W | ABC: Irritability: 16.0 ± 9.84 Hyperactivity: 21.9 ± 10.7 SRS: Social Cognition: 20.3 ± 5.63 Social Communication: IG 36.8 ± 10.04 SALT: Number different words used: 104.6 ± 58.45 Number words used: 219.2 ± 132.19 | ABC: Irritability: 9.5 ± 7.98 Hyperactivity: 14.3 ± 9.66 SRS: Social Cognition: 17.6 ± 5.55 Social Communication 30.2 ± 8.75 SALT: Number different words used: 116.7 ± 66.00 Number words used: 253.7 ± 154.62 | (-) | | ABC: Irritability: 16.1 ± 9.80 Hyperactivity: 21.0 ± 9.69 SRS: Social Cognition: 19.3 ± 5.58 Social Communication: 33.9 ± 8.84 SALT: Number different words used: 119.1 ± 64.55 Number words used: 277.6 ± 171.53 | ABC: Irritability: 13.6 ± 10.08 Hyperactivity: 18.4 ± 10.26 SRS: Social Cognition: 19.1 ± 5.64 Social Communication 33.6 ± 11.38 SALT: Number different words used: 118.4 ± 62.75 Number words used: 270.5 ± 162.88 | (-) | | (-) | |
| H Steiner, 2015 | Communication, self-care, motor skills and socialization | B/W | (-) | (-) | (-) | | (-) | (-) | (-) | | (-) | |
| Beth A Lanning, 2014 | Physical Functioning, Emotional Functioning, Social Functioning, and School Functioning | B/W | (-) | (-) | (-) | | (-) | (-) | (-) | | (-) | |
| Marguerite E O'Haire, 2014 | Social Approach, Social Withdrawal, Social Skills & Problem Behaviors | B/W | Parent measures - PDDBI:  Social Approach Behavior: 67.6 ± 14.2  Social Withdrawal Behaviors: 26.7 ± 10.6  Parent measures - SSRS: Social Skills: 76.0 ± 17.2  Problem Behaviors: 121.6 ± 14.1  Teacher measures - PDDBI:  Social Approach Behaviors: 55.9 ±14.8 Social Withdrawal Behaviors: 21.2 ± 14.2 Teacher measures -SSRS: Social Skills: 85.7 ± 15.4  Problem Behaviors: 111.9 ± 13.2 | (-) | (-) | | Parent measures - PDDBI:  Social Approach Behavior: 68.0 ± 12.0 Social Withdrawal Behaviors: 28.0 ± 9.9  Parent measures - SSRS:  Social Skills: 77.3 ± 12.5  Problem Behaviors: 119.1 ± 15.0  Teacher measures - PDDBI:  Social Approach Behaviors: 48.3 ± 15.6  Social Withdrawal Behaviors: 24.3 ± 12.0  Teacher measures - SSRS:  Social Skills: 82.1 ± 17.7  Problem Behaviors: 112.1 ± 13.4 | (-) | (-) | | (-) | |
| Margo B Holm, 2014 | Parent-nominated target  behaviors | W | (-) | (-) | (-) | | (-) | (-) | (-) | | (-) | |
| Fung, S.-C., 2014 | Social Interaction | B/W | (-) | (-) | SB: z = –2.02, p = 0.043 NSB: z = -2.03, p = 0.042 VSB: z = –2.02, p = 0.043 | | (-) | (-) | (-) | | (-) | |
| Heather F Ajzenman, 2013 | Social and Communication Skills | W | (-) | (-) | (-) | | (-) | (-) | (-) | | (-) | |
| Sandra C Ward, 2013 | Social communication and sensory processing skills | W | (-) | (-) | (-) | | (-) | (-) | (-) | | GARS:  Autism Index: F (5, 60) = 2.43, p < 0.05 Social Interaction: F (5, 60) = 4.61, p < 0.05 | |
| Ghorban, Hemati.， 2013 | Social skills | W | (-) | (-) | (-) | | (-) | (-) | (-) | | SSSS: Total score of social skills: sig.= 0.04 Subtest:  Affective Understanding/ perspective Taking and initiating interaction: sig.= 0.01 maintaining interaction: sig.= 0.003 | |
| Jenkins, Sarah R., 2013 | Behavior | n/a | (-) | (-) | (-) | | VABS: Socialization: 0.23 ± 0.21  Motor Skills: -0.26 ± 0.20 TOL:  Planning time: -6.46 ± 5.2 | (-) | (-) | | (-) | |
| Emílio Salgueiro, 2012 | Behavioral and cognition | W | (-) | (-) | (-) | | (-) | (-) | (-) | | CARS: Non-verbal Communication: F(3,24.407) = 2.151; p = 0.022 PER-R:  Overall development score: F(3,25.041) = 7.829; p = 0.001 Fine motor development: F(3, 25.174) = 4.54; p = 0.011 Cognitive performance: F(3, 25.079) = 4.333; p = 0.014 Cognitive verbal: F(3, 25.042) = 5.231; p = 0.006 | |
| Tabares, C., 2012 | Hormonal Changes | W | Hormone cortisol: 2.79 ± 0.52 ng/mL Hormone progesterone: 28.63 ± 12.81 pg/Ml | Hormone cortisol: Post Hippotherapy: 4.015 ± 1.59 ng/mL The rest of the Post Hippotherapy sessions: 2.23 ± 0.75 ng/mL Hormone progesterone: Post Hippotherapy: 51.59 ± 33.11 pg/mL The rest of the sessions: 26.03 ± 11.98 pg/mL | (-) | | (-) | (-) | (-) | | (-) | |
| MdYusof, 2012 | Stereotyped behaviors, communication, and social interaction | W | (-) | (-) | (-) | | (-) | (-) | (-) | | (-) | |
| Gabriels, Robin L., 2012 | Self-regulation, adaptive living skills, motor skills | B/W | ABC-C: Irritability 20.2 ± 8.9 Lethargy 12.4 ± 7.7 Stereotypy 6 ± 4.2 Hyperactivity 23.7 ± 9.9 VABS: Raw social score 104.9 ± 29.9 Raw communication score 143.6 ± 24.9 Raw daily score 110.6 ± 35.1 Adaptive total score 75.5 ± 10.4 Motor skills  BOT-2: 45.5 ± 15.5 SIPT: verbal score 16 ± 7.2 SIPT: postural score 19.5 ± 7.4 | ABC-C: Irritability 12.9 ± 8.5 Lethargy 6.3 ± 7.1 Stereotypy 3.3 ± 3.5 Hyperactivity 17.1 ± 11.6 VABS: Raw social score 113.2 ± 27.4 Raw communication score 149 ± 24.8 Raw daily score 117.4 ± 32.6 Adaptive total score 79.2 ± 11.3 Motor skills  BOT-2: 53.4 ± 15.2 SIPT: verbal score 18.8 ± 7 SIPT: postural score 22.9 ± 7.1 | (-) | | (-) | (-) | (-) | | (-) | |
| Janet K Kern, 2011 | Severity of autism symptoms, parent-child interactions | W | (-) | (-) | (-) | | (-) | (-) | (-) | | (-) | |
| Robert Viau, 2010 | Cortisol | W | (-) | (-) | (-) | | (-) | (-) | (-) | | Cortisol: F (2,42) = 53.6, p < 0.01 | |
| Taylor, Renee R., 2009 | Motivation | n/a | (-) | (-) | (-) | | (-) | (-) | (-) | | (-) | |
| Bass, M. M., 2009 | Social Functioning | B/W | SRS:  Social response scale: 85.9 ± 37.5 Social motivation: 17.3 ± 7.1 SP: Sensory profile: 237.6 ± 55.9 Sensory seeking: 58.4 ± 10.6 Inattention/distractibility: 21 ± 7.1 Sensory sensitivity: 15.7 ± 3.6 Sedentary: 13.5 ± 5 | SRS:  Social response scale: 73.6 ± 24.1 Social motivation: 12.5 ± 5.9 SP: Sensory profile: 269.4 ± 51.6 Sensory seeking: 62 ± 9 Inattention/distractibility: 27 ± 4.6 Sensory sensitivity: 17.2 ± 2.6 Sedentary: 16 ± 3.3 | (-) | | SRS:  Social response scale: 89.3 ± 35.4 Social motivation: 18.2 ± 7.1 SP: Sensory profile: 240.9 ± 50.9 Sensory seeking: 53.9 ± 10.9 Inattention/distractibility: 21.6 ± 4.6 Sensory sensitivity: 16.1 ± 4.6 Sedentary: 11.9 ± 5.1 | SRS:  Social response scale: 94.4 ± 32.1 Social motivation: 16.2 ± 6.7 SP: Sensory profile: 245.7 ± 50.3 Sensory seeking: 53.2 ± 10.5 Inattention/distractibility: 21.4 ± 4.5 Sensory sensitivity: 15.7 ± 4.8 Sedentary: 11.3 ± 4.8 | (-) | | (-) | |

Table 4 is sorted by chronology and abbreviation in alphabetical order.

Abbreviation: B, Between-group Effect; W, Within-group Effect; ABC-C, Aberrant Behavior Checklist–Community; SRS, Social Responsiveness Scale; SALT, Systematic Analysis of Language Transcripts; SSIS-RS, Social Skills Improvement System Ratting Scales; ABLLS-R, The Assessment of Basic Language and Learning Skills-Revised; GAS, Goal Attainment Scaling; BDCAS-P, Behavioral disorders of children with autism spectrum (parent form); TGMD-3, Test of Gross Motor Development-Third Edition; PA, Physical activity; DCDQ’07, Developmental Coordination Disorder Questionnaire, as revised in 2007; PSI-SF, Parenting Stress Index–Short Form; IEMS, Interaction Emotions Motor Skills; PER-R, Psychoeducational Profile Revised; GARS, Gilliam Autism Rating Scale; SP, Sensory Profile; VABS, Vineland Adaptive Behavioral Scales; BOT, Bruininks-Oseretsky Test; SIPT, Sensory Integration and Praxis Test; SSSS, Stone's social skills Scale; SB, Social Behaviors; NSB, non-Social Behaviors; VSB, verbal social behaviors; NVSB, nonverbal social behaviors; PDDBI, Pervasive Developmental Disorder Behavior Inventory; ASQ, Autism spectrum quotient; TOL: Tower of London; BOT2, Bruininks Oseretsky Test; REVT, Receptive and Expressive Vocabulary Test;
