# Supplementary material for: Effectiveness of animal-assisted activities and therapies for autism spectrum disorder: a systematic review and meta-analysis
Source: Front Vet Sci. 2024 Jun 3;11:1403527. doi: 10.3389/fvets.2024.1403527 (PMC11184216; doi:10.3389/fvets.2024.1403527)
Supplement: Supplementary file 3 [file Data_Sheet_1.docx]

**Supplementary Materials**

Ningkun Xiao, Vaishnavi Bagayi, Dandan Yang, Xinlin Huang, Zhong Lei, Sergey Kiselev, Mikhail A Bolkov, Irina A Tuzankina, Valery A Chereshnev

**Appendix 1. Search strategy**

**Appendix 2. List of included studies**

**Appendix 3. List of excluded studies**

**Appendix 4. List of systematic review or meta-analysis**

**Appendix 5. Major modifications for protocol in PROSPERO**

**Appendix 6. RoB Quality for RCTs**

**Appendix 7. ROBINS-I for non-RCTs**

**Appendix 8. Forest plot of social function**

**Appendix 9. PRISMA checklist**

**Appendix 1. Search strategy**

**PubMed**

| Step | Search Terms | Outcome |
| --- | --- | --- |
| #1 | (autism spectrum disorder [MeSH Terms]) OR (autistic spectrum disorder* [tw]) OR (ASD [tw]) OR (disorder, autistic spectrum [tw]) OR (asperger syndrome [tw]) OR (autis* [tw]) OR (Pervasive Developmental Disorders [MeSH Terms]) OR (Rett syndrome[tw]) OR (childhood disintegrative disorder[tw]) | 83151 |
| #2 | (Animal Assisted Therapy[MeSH Terms]) OR (Animal Assisted Therap* [tw]) OR (Therap*, Animal Assisted [tw]) OR (Animal Facilitated Therap* [tw]) OR (Animal Assisted Intervention* [tw]) OR (Animal Assisted Activit* [tw]) OR (Pet Therap* [tw]) OR (Pet Assisted Therap* [tw]) OR (Equine-Assisted Therapy[MeSH Terms]) OR (Equine Assisted Therap* [tw]) OR (Equine Assisted Psychotherap* [tw]) OR (Hippotherap* [tw]) OR (Horseback Riding Therap* [tw]) OR (Canine Assisted Therap* [tw]) OR (Dog Assisted Therap* [tw]) OR (Animal-Assisted Counseling[tw]) OR (Animal-Assisted Education[tw]) OR (Dolphin Assisted Therapy[tw]) | 1533 |
| #3 | (#1 AND #2) | 119 |

**Scopus**

| Step | Search Terms | Outcome |
| --- | --- | --- |
| #1 | (TITLE-ABS-KEY("Animal Assisted Therapy") OR TITLE-ABS-KEY("Animal Assisted Therap*") OR TITLE-ABS-KEY("Therap*, Animal Assisted") OR TITLE-ABS-KEY("Animal Facilitated Therap*") OR TITLE-ABS-KEY("Animal Assisted Intervention*") OR TITLE-ABS-KEY("Animal Assisted Activit*") OR TITLE-ABS-KEY("Pet Therap*") OR TITLE-ABS-KEY("Pet Assisted Therap*") OR TITLE-ABS-KEY("Equine-Assisted Therapy") OR TITLE-ABS-KEY("Equine Assisted Therap*") OR TITLE-ABS-KEY("Equine Assisted Psychotherap*") OR TITLE-ABS-KEY("Hippotherap*") OR TITLE-ABS-KEY("Horseback Riding Therap*") OR TITLE-ABS-KEY("Canine Assisted Therap*") OR TITLE-ABS-KEY("Dog Assisted Therap*") OR TITLE-ABS-KEY("Animal-Assisted Counseling") OR TITLE-ABS-KEY("Animal-Assisted Education") OR TITLE-ABS-KEY("Dolphin Assisted Therapy")) | 2961 |
| #2 | (TITLE-ABS-KEY("autism spectrum disorder") OR TITLE-ABS-KEY("autistic spectrum disorder*") OR TITLE-ABS-KEY("ASD") OR TITLE-ABS-KEY("disorder, autistic spectrum") OR TITLE-ABS-KEY("asperger syndrome") OR TITLE-ABS-KEY("autis*") OR TITLE-ABS-KEY("Pervasive Developmental Disorders") OR TITLE-ABS-KEY("Rett syndrome") OR TITLE-ABS-KEY("childhood disintegrative disorder")) | 135229 |
| #3 | (#1 AND #2) | 271 |
| #4 | #3 AND (LIMIT-TO (LANGUAGE, "English")) AND (LIMIT-TO (SRCTYPE, "j" )) | 222 |

**Google scholar**

We searched Google Scholar (https://scholar.google.com/) for 4620 publications using the keywords *"autism spectrum disorder" and "animal-assisted therapy"*. And then we screened the titles and abstracts of the literature for inclusion of eligible articles to ensure that we comprehensively included all eligible articles.

**Other additional publications**

We also searched for relevant systematic review and meta-analysis articles in Scopus and PubMed, of which there were 17 relevant articles in PubMed and 40 in Scopus and screened through these documents. We compiled X articles that met the requirements and were not duplicated.

**PubMed**

| Step | Search Terms | Outcome |
| --- | --- | --- |
| #1 | (autism spectrum disorder [MeSH Terms]) OR (autistic spectrum disorder* [tw]) OR (ASD [tw]) OR (disorder, autistic spectrum [tw]) OR (asperger syndrome [tw]) OR (autis* [tw]) OR (Pervasive Developmental Disorders [MeSH Terms]) OR (Rett syndrome[tw]) OR (childhood disintegrative disorder[tw]) | 82949 |
| #2 | (Animal Assisted Therapy[MeSH Terms]) OR (Animal Assisted Therap* [tw]) OR (Therap*, Animal Assisted [tw]) OR (Animal Facilitated Therap* [tw]) OR (Animal Assisted Intervention* [tw]) OR (Animal Assisted Activit* [tw]) OR (Pet Therap* [tw]) OR (Pet Assisted Therap* [tw]) OR (Equine-Assisted Therapy[MeSH Terms]) OR (Equine Assisted Therap* [tw]) OR (Equine Assisted Psychotherap* [tw]) OR (Hippotherap* [tw]) OR (Horseback Riding Therap* [tw]) OR (Canine Assisted Therap* [tw]) OR (Dog Assisted Therap* [tw]) OR (Animal-Assisted Counseling[tw]) OR (Animal-Assisted Education[tw]) OR (Dolphin Assisted Therapy[tw]) | 1527 |
| #3 | (#1 AND #2) | 118 |
| #4 | (meta-analysis [tw]) OR (systematic review [tw]) OR (systematic review [pt]) OR (meta analy* [tw]) OR (literature review[tw]) | 559098 |
| #5 | #3 AND #4 | 17 |

**Scopus**

| Step | Search Terms | Outcome |
| --- | --- | --- |
| #1 | (TITLE-ABS-KEY("Animal Assisted Therapy") OR TITLE-ABS-KEY("Animal Assisted Therap*") OR TITLE-ABS-KEY("Therap*, Animal Assisted") OR TITLE-ABS-KEY("Animal Facilitated Therap*") OR TITLE-ABS-KEY("Animal Assisted Intervention*") OR TITLE-ABS-KEY("Animal Assisted Activit*") OR TITLE-ABS-KEY("Pet Therap*") OR TITLE-ABS-KEY("Pet Assisted Therap*") OR TITLE-ABS-KEY("Equine-Assisted Therapy") OR TITLE-ABS-KEY("Equine Assisted Therap*") OR TITLE-ABS-KEY("Equine Assisted Psychotherap*") OR TITLE-ABS-KEY("Hippotherap*") OR TITLE-ABS-KEY("Horseback Riding Therap*") OR TITLE-ABS-KEY("Canine Assisted Therap*") OR TITLE-ABS-KEY("Dog Assisted Therap*") OR TITLE-ABS-KEY("Animal-Assisted Counseling") OR TITLE-ABS-KEY("Animal-Assisted Education") OR TITLE-ABS-KEY("Dolphin Assisted Therapy")) | 2953 |
| #2 | (TITLE-ABS-KEY("autism spectrum disorder") OR TITLE-ABS-KEY("autistic spectrum disorder*") OR TITLE-ABS-KEY("ASD") OR TITLE-ABS-KEY("disorder, autistic spectrum") OR TITLE-ABS-KEY("asperger syndrome") OR TITLE-ABS-KEY("autis*") OR TITLE-ABS-KEY("Pervasive Developmental Disorders") OR TITLE-ABS-KEY("Rett syndrome") OR TITLE-ABS-KEY("childhood disintegrative disorder")) | 134910 |
| #3 | (#1 AND #2) | 270 |
| #4 | TITLE-ABS-KEY ("meta-analysis") OR TITLE-ABS-KEY ("systematic review") OR TITLE-ABS-KEY ("meta analy*") OR TITLE-ABS-KEY ("literature review") | 905331 |
| #5 | #3 AND #4 | 40 |

**Appendix 2. List of included studies**

References of all trial studies included in the study:

| PubMed |
| --- |
| Randomized Controlled Trial of Therapeutic Horseback Riding in Children and Adolescents With Autism Spectrum Disorder. 2015. Robin L Gabriels, Zhaoxing Pan, Briar Dechant, John A Agnew, Natalie Brim, Gary Mesibov. J Am Acad Child Adolesc Psychiatry. DOI: 10.1016/j.jaac.2015.04.007. |
| Canine Assisted Occupational Therapy for Children on the Autism Spectrum: A Pilot Randomised Control Trial. 2020. Jessica Hill, et al., J Autism Dev Disord. DOI: 10.1007/s10803-020-04483-7. |
| Effects of a Therapeutic Horseback Riding Program on Social Interaction and Communication in Children with Autism. 2021. Mengxian Zhao, et al., Int J Environ Res Public Health. DOI: 10.3390/ijerph18052656. |
| Equine-Assisted Occupational Therapy: Increasing Engagement for Children With Autism Spectrum Disorder. 2016. Cecilia Llambias, et al., Am J Occup Ther. DOI: 10.5014/ajot.2016.020701. |
| Effects of Dog Assisted Therapy for Adults with Autism Spectrum Disorder: An Exploratory Randomized Controlled Trial. 2020. Carolien Wijker, et al., J Autism Dev Disord. DOI: 10.1007/s10803-019-03971-9. |
| Effects of therapeutic horse riding on gait cycle parameters and some aspects of behavior of children with autism. 2015. H Steiner, Zs Kertesz. Acta Physiol Hung. DOI: 10.1556/036.102.2015.3.10. |
| Effectiveness of a Standardized Equine-Assisted Therapy Program for Children with Autism Spectrum Disorder. 2016. Marta Borgi, et al., J Autism Dev Disord. DOI: 10.1007/s10803-015-2530-6. |
| The Impact of a Horse Riding Intervention on the Social Functioning of Children with Autism Spectrum Disorder. 2017. Androulla Harris, Joanne M Williams. Int J Environ Res Public Health. DOI: 10.3390/ijerph14070776. |
| Animal-assisted activity improves social behaviors in psychiatrically hospitalized youth with autism. 2019. Monique M Germone, et al., Autism. DOI: 10.1177/1362361319827411. |
| Pilot Study: Occupational Therapy in an Equine Environment for Youth With Autism. 2020. B Caitlin Peters, et al. OTJR (Thorofare N J). DOI: 10.1177/1539449220912723.  Effect of hippotherapy on motor control, adaptive behaviors, and participation in children with autism spectrum disorder: a pilot study. 2013. Heather F Ajzenman, et al., Am J Occup Ther. DOI: 10.5014/ajot.2013.008383.  Preliminary Efficacy of Occupational Therapy in an Equine Environment for Youth with Autism Spectrum Disorder. 2022. B Caitlin Peters, et al., J Autism Dev Disord. DOI: 10.1007/s10803-021-05278-0.  Improving social participation of children with autism spectrum disorder: Pilot testing of an early animal-assisted intervention in Spain. 2020. Adriana Ávila-Álvarez, et al., Health Soc Care Community. DOI: 10.1111/hsc.12955.  Animal-Assisted Activity for Children with Autism Spectrum Disorder: Parents' and Therapists' Perception. 2019. Ana L L Michelotto. J Altern Complement Med. DOI: 10.1089/acm.2019.0196.  Effects of equine assisted activities on autism spectrum disorder. 2014. Beth A Lanning, et al., J Autism Dev Disord. DOI: 10.1007/s10803-014-2062-5.  Prospective trial of equine-assisted activities in autism spectrum disorder. 2011. Janet K Kern, et al., Altern Ther Health Med. PMID: 22164808. |
| Effect of Motorized Elephant-Assisted Therapy Program on Balance Control of Children with Autism Spectrum Disorder. 2019. Satiansukpong Nuntanee, Sasat Daranee. Occup Ther Int. DOI: 10.1155/2019/5914807. |
| Long-Term Effect of Therapeutic Horseback Riding in Youth With Autism Spectrum Disorder: A Randomized Trial. 2018. Robin L Gabriels, et al., Front Vet Sci. DOI: 10.3389/fvets.2018.00156. |
| Effects of a dolphin interaction program on children with autism spectrum disorders: an exploratory research. 2012. Emílio Salgueiro, et al., BMC Res Notes. DOI: 10.1186/1756-0500-5-199. |
| Effectiveness of Equine-Assisted Activities and Therapies for Improving Adaptive Behavior and Motor Function in Autism Spectrum Disorder. 2021. Leonardo Zoccante, et al., J Clin Med. DOI: 10.3390/jcm10081726. |
| Effects of classroom animal-assisted activities on social functioning in children with autism spectrum disorder. 2014. Marguerite E O'Haire, et al., J Altern Complement Med. DOI: 10.1089/acm.2013.0165. |
| The effects of Animal Assisted Therapy on autonomic and endocrine activity in adults with autism spectrum disorder: A randomized controlled trial. 2021. Carolien Wijker, et al., Gen Hosp Psychiatry. DOI: 10.1016/j.genhosppsych.2021.05.003. |
| The association between therapeutic horseback riding and the social communication and sensory reactions of children with autism. 2013. Sandra C Ward, et al., J Autism Dev Disord. DOI: 10.1007/s10803-013-1773-3. |
| Brief Report: The Effects of Equine-Assisted Activities on the Social Functioning in Children and Adolescents with Autism Spectrum Disorder. 2016. Sophie Anderson, Kerstin Meints. J Autism Dev Disord. DOI: 10.1007/s10803-016-2869-3. |
| Therapeutic horseback riding outcomes of parent-identified goals for children with autism spectrum disorder: an ABA' multiple case design examining dosing and generalization to the home and community. 2014. Margo B Holm, et al., J Autism Dev Disord. DOI: 10.1007/s10803-013-1949-x. |
| Implementation of Assisted Therapy With Dogs in the Therapeutic Approach to People With Autistic Spectrum Disorder. 2020. Isabel Morales-Moreno, et al., Holist Nurs Pract. DOI: 10.1097/HNP.0000000000000403. |
|  |

Scopus

| Hippotherapy and its effect on behavioral and executive disorders in children with autism spectrum disorder. 2022. Rezapour-Nasrabad, R.R., et al., Journal of Advanced Pharmacy Education and Research. |
| --- |
| The Effects of Therapeutic Horseback Riding Program on Motor Skills in Children with Autism Spectrum Disorder. 2022. Zhao, M, et al., International Journal of Mental Health Promotion. |
| Dog-Assisted Physical Activity Intervention in Children with Autism Spectrum Disorder: A Feasibility and Efficacy Exploratory Study. 2022.  Abadi, M.R.H., et al., Anthrozoos. |
| The Feasibility and Acceptability of Occupational Therapy in an Equine Environment for Youth with Autism Spectrum Disorder. 2021. Hernández-Espeso, et al., Research in Autism Spectrum Disorders. |
| Effects of Dolphin-Assisted Therapy on the Social and Communication Skills of Children with Autism Spectrum Disorder. 2021. Hernández-Espeso, N., et al., Anthrozoos. |
| Evaluation of an equine therapy program in students with Autism spectrum disorder. 2020. Portela-Pino, I., et al., Journal of Human Sport and Exercise. |
| Pilot Study Investigating the Role of Therapy Dogs in Facilitating Social Interaction among Children with Autism. 2014. Fung, S.-C., Leung, A.S.-M. Journal of Contemporary Psychotherapy |
| Therapeutic horseback riding outcomes of parent-identified goals for children with autism spectrum disorder: An ABA′ multiple case design examining dosing and generalization to the home and community. 2014. Holm, M.B, et al., Journal of Autism and Developmental Disorders.  Quantification of hormonal changes by effects of hippotherapy in the autistic population. 2012. Tabares, C., et al., Neurochemical Journal.  Dolphin encounter for special children (DESC) program: Effectiveness of dolphin-assisted therapy for children with autism. 2012. MdYusof, M.S.B., Chia, N.K.H. International Journal of Special Education  Guinea pigs-the small great therapist for autistic children, or: Do guinea pigs have positive effects on autistic child social behavior? 2010. Kršková, L., Talarovičová, A., Olexová, L. Society and Animals  Thai Elephant-assisted Therapy Program: The Feasibility in Assisting an Individual with Autism. 2008. Satiansukpong, N., et al., World Federation of Occupational Therapists Bulletin  Volitional Change in Children With Autism: A Single-Case Design Study of the Impact of Hippotherapy on Motivation. 2009. Renee R. Taylor, et al., Occupational Therapy in Mental Health. |

| Google scholar |
| --- |
| Effect of service dogs on salivary cortisol secretion in autistic children. 2010. Robert Viau et al., Psychoneuroendocrinology.  The Effect of Therapeutic Horseback Riding on Social Functioning in Children with Autism. 2009. Margaret M. Bass, et al., Journal of Autism and Developmental Disorders.  Pilot study measuring the effects of therapeutic horseback riding on school-age children and adolescents with autism spectrum disorders. 2012. Robin L. Gabriels, et al., Research in Autism Spectrum Disorders.  Effectiveness of Therapeutic Horseback Riding on Social Skills of Children with Autism Spectrum Disorder in Shiraz, Iran. 2013. Hemati Ghorban, et al., Journal of Education and Learning.  An experimental analysis of the effects of therapeutic horseback riding on the behavior of children with autism. 2013. Sarah R. Jenkins, et al., Research in Autism Spectrum Disorders. |

**Appendix 3. List of excluded studies**

References of all trial studies excluded in the study:

| Publications | Excluded reason |
| --- | --- |
| PubMed | |
| Autism and Equine-Assisted Interventions: A Systematic Mapping Review. 2017. B Caitlin McDaniel Peters, Wendy Wood. J Autism Dev Disord. DOI: 10.1007/s10803-017-3219-9 | Mapping review |
| Animal Assisted Therapy for Children and Adolescents with Autism Spectrum Disorder: Parent perspectives. 2020. Maeve Doyle London, Lynette Mackenzie, Meryl Lovarini, Claire Dickson, Alberto Alvarez-Campos. DOI: 10.1007/s10803-020-04512-5 | Parent perspectives |
| Companion animals and human health: benefits, challenges, and the road ahead for human-animal interaction. E Friedman, C A Krause-Parello. 2018. Rev Sci Tech. DOI: 10.20506/rst.37.1.2741. | Not for ASD |
| Animal-assisted intervention for autism spectrum disorder: a systematic literature review. 2013. Marguerite E O'Haire. J Autism Dev Disord. DOI: 10.1007/s10803-012-1707-5. | Systematic review |
| What is hippotherapy? The indications and effectiveness of hippotherapy. 2016. Tuba Tulay Koca, Hilmi Ataseven. North Clin Istanb. DOI: 10.14744/nci.2016.71601. | Perspectives |
| Project AIM: Autism intervention meta-analysis for studies of young children. 2020. Micheal Sandbank, et al., Psychol Bull. DOI: 10.1037/bul0000215. | Meta-analysis |
| Animal-assisted interventions as innovative tools for mental health. 2011. Francesca Cirulli, et al., Ann Ist Super Sanita. DOI: 10.4415/ANN_11_04_04. | Review |
| Effects of Equine Therapy on Individuals with Autism Spectrum Disorder: A Systematic Review. 2018. Sudha M Srinivasan, et al., Rev J Autism Dev Disord. DOI: 10.1007/s40489-018-0130-z. | Systematic review |
| Autism spectrum disorder and pet therapy. 2015. Caitlin M Siewertsen, et al., Adv Mind Body Med. PMID: 25831431. | Review |
| Equine assisted activities and therapies in children with autism spectrum disorder: A systematic review and a meta-analysis. 2019. Tomasz Trzmiel, et al., Complement Ther Med. DOI: 10.1016/j.ctim.2018.11.004. | Systematic review and meta-analysis |
| 'It just opens up their world': autism, empathy, and the therapeutic effects of equine interactions. 2018. Roslyn Malcolm, et al., Anthropol Med. DOI: 10.1080/13648470.2017.1291115. | Not trial |
| Effectiveness of Animal-Assisted Therapy in the Pediatric Population: Systematic Review and Meta-Analysis of Controlled Studies. 2018. Jesús David Charry-Sánchez, et al., J Dev Behav Pediatr. DOI: 10.1097/DBP.0000000000000594. | Systematic review and meta-analysis |
| Hippotherapy in neurodevelopmental disorders: a narrative review focusing on cognitive and behavioral outcomes. 2022. Giuseppa Maresca, et al., Appl Neuropsychol Child. DOI: 10.1080/21622965.2020.1852084. | Narrative review |
| Dog training alleviates PTSD symptomatology by emotional and attentional regulation. 2021. Inon Maoz, et al., Eur J Psychotraumatol. DOI: 10.1080/20008198.2021.1995264. | PTSD |
| Effects of Therapeutic Horseback-Riding Program on Social and Communication Skills in Children with Autism Spectrum Disorder: A Systematic Review and Meta-Analysis. 2022. Shihui Chen, et al., Int J Environ Res Public Health. DOI: 10.3390/ijerph192114449. | Systematic review and meta-analysis |
| Effects of Equine-Assisted Activities and Therapies for Individuals with Autism Spectrum Disorder: Systematic Review and Meta-Analysis. 2023. Ningkun Xiao, et al., Int J Environ Res Public Health. DOI: 10.3390/ijerph20032630. | Systematic review and meta-analysis |
| Calm with horses? A systematic review of animal-assisted interventions for improving social functioning in children with autism. 2022. Jon H Sissons, et al., Autism. DOI: 10.1177/13623613221085338. | Systematic review |
| Putting Cats on the Spectrum: A Scoping Review of the Role of Cats in Therapy and Companionship for Autistic Adults and Children. 2023. Michelle Cleary, et al., Issues Ment Health Nurs. DOI: 10.1080/01612840.2023.2195509. | Scoping review |
| Using Animal-assisted Therapy to Enrich Psychotherapy. 2016. Jeanne Louise Amerine, Grace B Hubbard. Adv Mind Body Med. PMID: 27541053. | Not for ASD |
| Reflections on Recent Research Into Animal-Assisted Interventions in the Military and Beyond. 2017. Christina B Rumayor, Amy M Thrasher. Curr Psychiatry Rep. DOI: 10.1007/s11920-017-0861-z. | Not for ASD |
| Canine-assisted occupational therapy for children on the autism spectrum: Parents' perspectives. 2020. Jessica Rachel Hill, et al., Aust Occup Ther J. DOI: 10.1111/1440-1630.12659. | Perspectives |
| Equine assisted services impact on social skills in autism spectrum disorder: A meta-analysis. 2023. Jérémy Madigand, et al., Prog Neuropsychopharmacol Biol Psychiatry. DOI: 10.1016/j.pnpbp.2023.110765. | Meta-analysis |
| Animal-Assisted Interventions for School-Aged Children with Autism Spectrum Disorder: A Meta-Analysis. 2021. Mirena Dimolareva, Thomas J Dunn. J Autism Dev Disord. DOI: 10.1007/s10803-020-04715-w. | Meta-analysis |
| An Evaluation of Animal-Assisted Therapy for Autism Spectrum Disorders: Therapist and Parent Perspectives. 2022. Chin-Siang Ang, Freya Anne MacDougall. Psychol Stud (Mysore). DOI: 10.1007/s12646-022-00647-w. | Perspectives |
| Attitudes to and beliefs about animal assisted therapy for children with disabilities. 2017. Esther Yap, et al., Complement Ther Clin Pract. DOI: 10.1016/j.ctcp.2016.11.009. | Perspectives |
| The influence of animals on the development of children. 2011. Nienke Endenburg, Hein A van Lith. Vet J. DOI: 10.1016/j.tvjl.2010.11.020. | Not trial |
| Animal-assisted interventions in internal and rehabilitation medicine: a review of the recent literature. 2011. S Muñoz Lasa, et al., Panminerva Med. PMID: 21659977. | Review |
| Equine Assisted Therapy for Patients with Post Traumatic Stress Disorder: A Case Series Study. 2019. Assaf Shelef, et al., Mil Med. DOI: 10.1093/milmed/usz036. | PTSD |
| Third time's the charm or three strikes you're out? An updated review of the efficacy of dolphin-assisted therapy for autism and developmental disabilities. 2021. Lori Marino, Scott O Lilienfeld. J Clin Psychol. DOI: 10.1002/jclp.23110. | Review |
| Research on animal-assisted intervention and autism spectrum disorder, 2012-2015. 2017. Marguerite O'Haire. Appl Dev Sci. DOI: 10.1080/10888691.2016.1243988. | Review |
| Alternative therapeutic intervention for individuals with Rett syndrome. 2007. Meir Lotan. ScientificWorldJournal. DOI: 10.1100/tsw.2007.4. | Rett syndrome |
| Australian parents' experiences of owning an autism assistance dog. 2022. Rebecca Appleby, et al., Health Soc Care Community. DOI: 10.1111/hsc.13805. | Perspectives |
| An Overview of the Available Intervention Strategies for Postural Balance Control in Individuals with Autism Spectrum Disorder. 2022. Rabeeh Hariri. DOI: 10.1155/2022/3639352. | Review |
| The effectiveness of animal-assisted therapy for children and adolescents with autism spectrum disorder: A systematic review. 2023. Amy Kate Rehn, et al., Complement Ther Clin Pract. DOI: 10.1016/j.ctcp.2022.101719. | Systematic review |
| Horseback riding therapy for a deafblind individual enabled by a haptic interface. 2018. Matjaž Ogrinc, e al., Assist Technol. DOI: 10.1080/10400435.2017.1288178. | Deafblind |
| Dolphin-Assisted Therapy: Claims versus Evidence. 2012. Britta L Fiksdal, et al., Autism Res Treat. DOI: 10.1155/2012/839792. | Claims versus Evidence |
| Benefits of animal-Assisted interventions in preschool children: A systematic review. 2023. Ana Myriam Lavín-Pérez, et al., Clin Child Psychol Psychiatry. DOI: 10.1177/13591045221142115. | Systematic review |
| Animal-Assisted Therapies for Youth with or at risk for Mental Health Problems: A Systematic Review. 2017. Kimberly Eaton Hoagwood, et al., Appl Dev Sci. DOI: 10.1080/10888691.2015.1134267. | Systematic review |
| Use of assistance and therapy dogs for children with autism spectrum disorders: a critical review of the current evidence. 2013. Alessandra Berry, et al., J Altern Complement Med. DOI: 10.1089/acm.2011.0835. | Review |
| Parental Perspectives of Occupational Therapy in an Equine Environment for Children with Autism Spectrum Disorder. 2020. Dorothy Kalmbach, et al., Occup Ther Health Care. DOI: 10.1080/07380577.2020.1751903. | Perspectives |
| Setting the One Health agenda and the human-companion animal bond. 2014. Gregg K Takashima, Michael J Day. Int J Environ Res Public Health. DOI: 10.3390/ijerph111111110. | Not trial |
| Social Development of Adults with Autism Spectrum Disorder During Dog-Assisted Therapy: A Detailed Observational Analysis. 2020. Carolien Wijker, et al., Int J Environ Res Public Health. DOI: 10.3390/ijerph17165922. | Observational analysis |
| Assessing Preferences for Animals in Children with Autism: A New Use for Video-Based Preference Assessment. 2017. Noémie A Guérin, et al., Front Vet Sci. DOI: 10.3389/fvets.2017.00029. | Not intervention |
| Evaluating preference for and reinforcing efficacy of a therapy dog to increase verbal statements. 2020. Courtney D Jorgenson, et al., J Appl Behav Anal. DOI: 10.1002/jaba.668. | Evaluating preference |
| The psychiatric aspects of animal assisted therapy. 2012. Noémi Bánszky, et al., Psychiatr Hung. PMID: 22781543. | Psychiatric |
| Complementary and alternative medicine use in adults with autism spectrum disorder in Germany: results from a multi-center survey. 2019. Juliana Höfe, et al., BMC Psychiatry. DOI: 10.1186/s12888-019-2043-5. | Survey |
| Animal Interaction Affecting Core Deficit Domains Among Children with Autism: A Meta-Analysis. 2021. Michael J Droboniku, Matthew P Mychailyszyn. J Autism Dev Disord. DOI: 10.1007/s10803-021-04891-3. | Meta-analysis |
| Parent Perceptions of Psychosocial Outcomes of Equine-Assisted Interventions for Children with Autism Spectrum Disorder. 2018. Vanessa Xue-Ling Tan, Janette Graetz Simmonds. J Autism Dev Disord. DOI: 10.1007/s10803-017-3399-3. | Perspectives |
| Effects of Dog-Assisted Education on Physical and Communicative Skills in Children with Severe and Multiple Disabilities: A Pilot Study. 2021. Luis Lucio Lobato Rincón, et al., Animals (Basel). DOI: 10.3390/ani11061741. | Severe and Multiple Disabilities |
| Social rivalry triggers visual attention in children with autism spectrum disorders. 2017. Marine Grandgeorge, et al., Sci Rep. DOI: 10.1038/s41598-017-09745-6. | Not intervention |
| "The connection just happens": Therapists' perspectives of canine-assisted occupational therapy for children on the autism spectrum. 2020. Jessica Rachel Hill, et al., Aust Occup Ther J. DOI: 10.1111/1440-1630.12680. | Perspectives |
| Animal-Assisted Interventions for Autism Spectrum Disorder: A Systematic Review of the Literature from 2016 to 2020. 2023. Leanne O Nieforth, et al., Rev J Autism Dev Disord. DOI: 10.1007/s40489-021-00291-6. | Systematic review and meta-analysis |
| Psycho-educational Horseback Riding to Facilitate Communication Ability of Children with Pervasive Developmental Disorders. 2009. Hiromi Keino, et al., J Equine Sci. DOI: 10.1294/jes.20.79. | Pervasive Developmental Disorders |
| Autistic rider. 2009. Carl A Hammerschlag. PMID: 19772030 | Not avaliable |
| Design and development of a Virtual Dolphinarium for children with autism. 2013. Yiyu Cai, et al., IEEE Trans Neural Syst Rehabil Eng. DOI: 10.1109/TNSRE.2013.2240700. | Virtual Dolphinarium |
| A Stakeholder-Engaged Approach to Development of an Animal-Assisted Intervention for Obesity Prevention Among Youth With Autism Spectrum Disorder and Their Pet Dogs. 2021. Deborah E Linder, et al., Front Vet Sci. DOI: 10.3389/fvets.2021.735432. | Obesity Prevention |
| Impact of a 12-month multifaceted neurological physiotherapy intervention on gross motor function in women with Rett syndrome. 2022. Alen Kapel, et al., J Integr Neurosci. DOI: 10.31083/j.jin2102059. | Rett syndrome |
| Evidence-based analysis of multi-pronged approaches for education and behavior management of autistic patients in a dental setting. 2023. Tavisha Goyal, et al., Spec Care Dentist. DOI: 10.1111/scd.12867. | Evidence-based analysis |
| Therapeutic Horseback Riding Crossover Effects of Attachment Behaviors with Family Pets in a Sample of Children with Autism Spectrum Disorder. 2017. Jessie D Petty, et al., Int J Environ Res Public Health. DOI: 10.3390/ijerph14030256. | For pets |
| Changes in behavioural synchrony during dog-assisted therapy for children with autism spectrum disorder and children with Down syndrome. 2020. Richard Eric Griffioen, et al., J Appl Res Intellect Disabil. DOI: 10.1111/jar.12682. | Down syndrome |
| Verbal Interactional Synchronization between Therapist and Children with Autism Spectrum Disorder during Dolphin Assisted Therapy: Five Case Studies. 2019. Richard Griffioen, et al., Animals (Basel). DOI: 10.3390/ani9100716. | Case |
| Animal-Assisted Intervention: A Promising Approach to Obesity Prevention for Youth With Autism Spectrum Disorder. 2021. Aviva Must, et al., Front Vet Sci. DOI: 10.3389/fvets.2021.646081. | Obesity Prevention |
| A thematic analysis of influences on parents' autism intervention decisions. 2021. Meghan Wilson, et al., Res Dev Disabil. DOI: 10.1016/j.ridd.2021.104035. | Thematic analysis |
| Editorial: Children and Companion Animals: Psychosocial, Medical and Neurobiological Implications. 2018. Andrea Beetz, et al., Front Vet Sci. OI: 10.3389/fvets.2018.00112. | Editorial |
| Circumscribed interests in autism: Can animals potentially re-engage social attention? 2023. Georgitta J Valiyamattam, et al., Res Dev Disabil. DOI: 10.1016/j.ridd.2023.104486. | Not intervention |
| Equine-Assisted Intervention in a child diagnosed with autism spectrum disorder: a case report. 2016. Stefania Cerino, et al., Riv Psichiatr. DOI: 10.1708/2596.26730. | Case |
| Comparison of contingent and noncontingent access to therapy dogs during academic tasks in children with autism spectrum disorder. 2020. Alexandra Protopopova, et al., J Appl Behav Anal. DOI: 10.1002/jaba.619. | Not core syndrome |
| The influence of equine-assisted services on the balance of a participant with visual impairment and autism characteristics. 2022. Marieli Matias Ramos, Marli Nabeiro. J Bodyw Mov Ther. DOI: 10.1016/j.jbmt.2022.03.007. | Case |
| Advancing Methods in Animal-Assisted Intervention: Demonstration of Starting Points in Clinical Practice for Children with Autism Spectrum Disorder. 2022. Casey J Clay, et al., Behav Anal Pract. DOI: 10.1007/s40617-022-00704-w. | Not intervention |
| Process Evaluation of Animal-Assisted Therapy: Feasibility and Relevance of a Dog-Assisted Therapy Program in Adults with Autism Spectrum Disorder. 2019. Carolien Wijker, et al., Animals (Basel). DOI: 10.3390/ani9121103. | Not trial |
| Do Animals Perceive Human Developmental Disabilities? Guinea Pigs' Behaviour with Children with Autism Spectrum Disorders and Children with Typical Development. A Pilot Study. 2019. Marine Grandgeorge, et al., Animals (Basel). DOI: 10.3390/ani9080522. | Not intervention |
| Can Dogs Assist Children with Severe Autism Spectrum Disorder in Complying with Challenging Demands? An Exploratory Experiment with a Live and a Robotic Dog. 2018. Karine Silva, et al., J Altern Complement Med. DOI: 10.1089/acm.2017.0254. | Exploratory experiment |
| What Factors Are Associated with Positive Effects of Dog Ownership in Families with Children with Autism Spectrum Disorder? The Development of the Lincoln Autism Pet Dog Impact Scale. 2016. Sophie Susannah Hall, et al., PLoS One. DOI: 10.1371/journal.pone.0149736. | Scale |
| Vitality from Experiences in Nature and Contact with Animals-A Way to Develop Joint Attention and Social Engagement in Children with Autism? 2019. Kristina Byström, et al., Int J Environ Res Public Health. DOI: 10.3390/ijerph16234673. | Multiple interventions |
| Human-Animal Interaction Research: Progress and Possibilities. 2019. James A Griffin, et al., Front Psychol. DOI: 10.3389/fpsyg.2019.02803. | Not intervention |
| Does pet arrival trigger prosocial behaviors in individuals with autism? 2012. Marine Grandgeorge, et al., PLoS One. DOI: 10.1371/journal.pone.0041739. | Not intervention |
| Evaluating Animal-Assisted Interventions: An Empirical Illustration of Differences between Outcome Measures. 2019. Steffie van der Steen, et al., Animals (Basel). DOI: 10.3390/ani9090645. | Not trial |
| Parents' perspectives on the value of assistance dogs for children with autism spectrum disorder: a cross-sectional study. 2014. Louise Burgoyne, et al., BMJ Open. DOI: 10.1136/bmjopen-2014-004786. | Perspectives |
| Reliability and Validity Assessment of the Observation of Human-Animal Interaction for Research (OHAIRE) Behavior Coding Tool. 2018. Noémie A Guérin, et al., Front Vet Sci. DOI: 10.3389/fvets.2018.00268. | Not intervention |
| Brief report: the smiles of a child with autism spectrum disorder during an animal-assisted activity may facilitate social positive behaviors--quantitative analysis with smile-detecting interface. 2014. Atsushi Funahashi, et al., J Autism Dev Disord. DOI: 10.1007/s10803-013-1898-4. | Brief report |
| Animals may act as social buffers: Skin conductance arousal in children with autism spectrum disorder in a social context. 2015. Marguerite E O'Haire, et al., Dev Psychobiol. DOI: 10.1002/dev.21310. | Not core syndrome |
| ttenuated adenylosuccinate lyase deficiency: a report of one case and a review of the literature. 2014. Agnieszka Jurecka, et al., Neuropediatrics. DOI: 10.1055/s-0033-1337335. | Not ASD |
| The effectiveness of simulated developmental horse-riding program in children with autism. 2010. Yee-Pay Wuang, et al., Adapt Phys Activ Q. DOI: 10.1123/apaq.27.2.113, | Simulated horse-riding |
| Increasing the Social Communication of a Boy With Autism Using Animal-assisted Play Therapy: A Case Report. 2015. Suk Chun Fung. Adv Mind Body Med. PMID: 26026154. | Case |
| Mira and autism. 2015. Perspect Infirm. | Not available |
| Hippotherapy and life habits with children with motor deficit and neurodevelopmental impairment: A pilot survey of parents. 2021. Alex Potvin-Bélanger, et al., J Pediatr Rehabil Med. DOI: 10.3233/PRM-190641. | Neurodevelopmental impairment |
| Hippotherapy in the paedopsychiatric care project. 2011. Laurence Hameury, et al., Soins Pediatr Pueric. PMID: 21328838. | Paedopsychiatric |
| Can dogs prime autistic children for therapy? Evidence from a single case study. 2011. Karine Silva, et al., J Altern Complement Med. DOI: 10.1089/acm.2010.0436. | Case |
| Play with online virtual pets as a method to improve mirror neuron and real world functioning in autistic children. 2008. Eric Lewin Altschuler. Med Hypotheses. DOI: 10.1016/j.mehy.2007.07.030. | Virtual pets |
| "But-He'll Fall!": Children with Autism, Interspecies Intersubjectivity, and the Problem of 'Being Social'. 2015. Olga Solomon. Cult Med Psychiatry. DOI: 10.1007/s11013-015-9446-7. | Case |
| Lost in a lost world. 2011. William Benda. ltern Ther Health Med. PMID: 22164807. | Not for ASD |
| The effect of dolphin-assisted therapy sessions on the functional status of children with psychoneurological disease symptoms. 1999. L N Lukina. Fiziol Cheloveka. PMID: 10641384. | Russian |
| Randomised controlled study of inter-hemispheric electroencephalographic coherence following assisted therapy with dolphins in children with autism spectrum disorders. 2018. P Ortiz-Sanchez, et al., Rev Neurol. PMID: 29516455 | Spanish |
|  |  |
| Scopus |  |
| Equine assisted services impact on social skills in autism spectrum disorder: A meta-analysis. 2023.  Madigand, J., Rio, M., Vandevelde, A. Progress in Neuro-Psychopharmacology and Biological Psychiatry. | Duplication |
| Circumscribed interests in autism: Can animals potentially re-engage social attention? 2023.  Valiyamattam, G.J., et al., Research in Developmental Disabilities. | Duplication |
| Animal-Assisted Interventions for Autism Spectrum Disorder: A Systematic Review of the Literature from 2016 to 2020. 2023. Nieforth, L.O., et al., Review Journal of Autism and Developmental Disorders. | Duplication |
| ‘The best way to get it right is to listen to us’ — autistic people argue for a stronger voice in research. 2023.  Rodríguez Mega, E. Nature. | Perspectives |
| The Healthcare Experience of Autistic Patients in Orthopaedic Surgery and Closely Related Fields: A Scoping Review. 2023. Criss, S.D., et al., Children. | Scoping Review |
| Benefits of animal-Assisted interventions in preschool children: A systematic review. 2023. Lavín-Pérez, A.M., et al., Clinical Child Psychology and Psychiatry. | Duplication |
| Experiences of equine assisted therapy for females with autism spectrum disorders. 2023. Warner, K., et al., Advances in Autism. | Perspectives |
| Effects of Equine-Assisted Activities and Therapies for Individuals with Autism Spectrum Disorder: Systematic Review and Meta-Analysis. 2023. Xiao et al., International Journal of Environmental Research and Public Health. | Duplication |
| The effectiveness of animal-assisted therapy for children and adolescents with autism spectrum disorder: A systematic review. 2023. Rehn, A.K., et al., Complementary Therapies in Clinical Practice. | Duplication |
| Non-pharmacological interventions for autistic children: An umbrella review. 2023. Trembath, D., et al. Autism | Umbrella review |
| Evidence-based analysis of multi-pronged approaches for education and behavior management of autistic patients in a dental setting. 2023. Goyal, T., et al., Special Care in Dentistry. | Duplication |
| Putting Cats on the Spectrum: A Scoping Review of the Role of Cats in Therapy and Companionship for Autistic Adults and Children. 2023. Cleary, M. et al., Issues in Mental Health Nursing. | Duplication |
| The Potential and Effects of Equine-Assisted Activities in a Day Care Center for Children and Adolescents with Developmental Disorders. 2023. Hayashibara, C. Occupational Therapy in Mental Health. | ADHD |
| Clinical EEG of Rett Syndrome: Group Analysis Supplemented with Longitudinal Case Report. 2022. Portnova, G., et al., Journal of Personalized Medicine. | Rett Syndrome |
| Effects of Therapeutic Horseback-Riding Program on Social and Communication Skills in Children with Autism Spectrum Disorder: A Systematic Review and Meta-Analysis. 2022. Chen, S., et al., International Journal of Environmental Research and Public Health. | Duplication |
| Occupational therapy incorporating dogs for autistic children and young people: Parent perspectives. 2022.  Richardson, K., et al., British Journal of Occupational Therapy. | Perspectives |
| Australian parents' experiences of owning an autism assistance dog. 2022. Rebecca Appleby, et al., Health Soc Care Community. | Duplication |
| Dog Therapy in Supporting the Rehabilitation Process of Children with Autism \| Dogoterapia we wspomaganiu procesu rehabilitacji dzieci z autyzmem. 2022. Marszałek, A., et al., Rehabilitacja Medyczna. | Review |
| The Benefits of Dog-Assisted Therapy as Complementary Treatment in a Children’s Mental Health Day Hospital. 2022. Guillen Guzmán, E., et al., Animals. | Mental health |
| Preliminary Efficacy of Occupational Therapy in an Equine Environment for Youth with Autism Spectrum Disorder. 2022. B Caitlin Peters, et al., J Autism Dev Disord. | Duplication |
| Role of pets and animal assisted therapy in suicide prevention. 2022. shoib, S., et al., Annals of Medicine and Surgery. | Suicide prevention |
| Calm with horses? A systematic review of animal-assisted interventions for improving social functioning in children with autism. 2022. Jon H Sissons, et al., Autism. | Duplication |
| Selected Methods of Therapeutic Interactions With People With Mild Symptoms of Autism Spectrum Disorder. 2022. Marta, K., et al., Frontiers in Psychiatry. | Review |
| The influence of equine-assisted services on the balance of a participant with visual impairment and autism characteristics. 2022. Marieli Matias Ramos, Marli Nabeiro. J Bodyw Mov Ther. | Duplication |
| The influence of equine-assisted services on the balance of a participant with visual impairment and autism characteristics. 2022. Peters, B.C., et al., Frontiers in Pediatrics. | Exploratory secondary analysis |
| Can dogs reduce stress levels in school children? effects of dog-assisted interventions on salivary cortisol in children with and without special educational needs using randomized controlled trials. 2022. Meints, K., et al., PLoS ONE. | Not for ASD |
| Impact of a 12-month multifaceted neurological physiotherapy intervention on gross motor function in women with Rett syndrome. 2022. Alen Kapel, et al., J Integr Neurosci. | Duplication |
| An Evaluation of Animal-Assisted Therapy for Autism Spectrum Disorders: Therapist and Parent Perspectives. 2022. Chin-Siang Ang, Freya Anne MacDougall. Psychol Stud (Mysore). | Duplication |
| A Systematic Review of Dog-Assisted Therapy in Children with Behavioural and Developmental Disorders. 2022. Kapel, A., et al., Journal of Integrative Neuroscience. | Behavioural and Developmental Disorders |
| Canine-assisted Therapy in Neurodevelopmental Disorders: A Scoping Review. 2022. Narvekar, H.N. European Journal of Integrative Medicine. | Neurodevelopmental Disorders |
| Engaging with animal-assisted interventions (AAIs): exploring the experiences of young people with ASD/ADHD diagnoses. Chadwick, Z., et al., Support for Learning. | Perspectives |
| A callosal biomarker of behavioral intervention outcomes for autism spectrum disorder? A case-control feasibility study with diffusion tensor imaging. 2022. Virues-Ortega, J., et al., PLoS ONE. | Not AAI |
| Early Support for the Development of Children with Autistic Spectrum Disorder: a Case Study. 2022. Płoszaj, M. | Case |
| Characterization of Children with Autism Spectrum Disorder’s Interactions with a Service Dog During Their First Encounter. 2022. Dollion, N., et al., Anthrozoos. | Not intervention |
| Pervasive Refusal Syndrome: Three Case Reports - Autism as a Predisposing Factor and Gentle Coercion to Shorten Duration of Disorder? 2022. Jarbin, H., et al., Case Reports in Psychiatry. | Case |
| The efficacy of executive function interventions in children with autism spectrum disorder: a systematic review and meta-analysis. 2022. Cavalli, G., et al., Expert Review of Neurotherapeutics. | Systematic review and meta-analysis |
| Hippotherapy in neurodevelopmental disorders: a narrative review focusing on cognitive and behavioral outcomes. 2022. Giuseppa Maresca, et al., Appl Neuropsychol Child. | Duplication |
| Animal Interaction Affecting Core Deficit Domains Among Children with Autism: A Meta-Analysis. 2021. Michael J Droboniku, Matthew P Mychailyszyn. J Autism Dev Disord. | Duplication |
| The Impact of Animal Exposure for Children with ASD: a Scoping Review. 2021. Lisk, C., et al., Review Journal of Autism and Developmental Disorders. | Scoping Review |
| A Stakeholder-Engaged Approach to Development of an Animal-Assisted Intervention for Obesity Prevention Among Youth With Autism Spectrum Disorder and Their Pet Dogs. 2021. Deborah E Linder, et al., Front Vet Sci. | Duplication |
| Using Telehealth to Deliver Family-Based Cognitive Behavioral Treatment of Insomnia in a School-Aged Child With Autism Spectrum Disorder. 2021. Davenport, M.A., et al., Journal of Cognitive Psychotherapy. | Not AAI |
| 2021 AAHA Working, Assistance, and Therapy Dog Guidelines. 2021. Otto, C.M., et al., Journal of the American Animal Hospital Association. | Guidelines |
| Effects of dog-assisted therapy on the physical function and communication skills of adults with autism: A study protocol for a controlled study. 2021. Gómez-Calcerrada, I., et al., Applied Sciences (Switzerland). | Protocol |
| Opinions of Turkish parents of children with autism spectrum disorder on use of complementary and alternative medicine methods. 2021. Cevik Guner, U., et al., Research in Autism Spectrum Disorders. | Perspectives |
| A thematic analysis of influences on parents' autism intervention decisions. 2021. Meghan Wilson, et al., Res Dev Disabil. | Duplication |
| The effects of Animal Assisted Therapy on autonomic and endocrine activity in adults with autism spectrum disorder: A randomized controlled trial. 2021. Carolien Wijker, et al., Gen Hosp Psychiatry. | Duplication |
| Animal-Assisted Interventions for School-Aged Children with Autism Spectrum Disorder: A Meta-Analysis. 2021. Mirena Dimolareva, Thomas J Dunn. J Autism Dev Disord. | Duplication |
| Note: Marino, L., Lilienfeld, S.O. Third time's the charm or three strikes you're out? An updated review of the efficacy of dolphin-assisted therapy for autism and developmental disabilities. J Clin Psychol. 1–15 (2021). <https://doi.org/10.1002/jclp.23110>. | Not available |
| Effects of Dog-Assisted Education on Physical and Communicative Skills in Children with Severe and Multiple Disabilities: A Pilot Study. 2021. Luis Lucio Lobato Rincón, et al., Animals (Basel). | Duplication |
| Third time's the charm or three strikes you're out? An updated review of the efficacy of dolphin-assisted therapy for autism and developmental disabilities. 2021. Lori Marino, Scott O Lilienfeld. J Clin Psychol. | Duplication |
| Animal-Assisted Intervention: A Promising Approach to Obesity Prevention for Youth With Autism Spectrum Disorder. 2021. Aviva Must, et al., Front Vet Sci. | Duplication |
| Effectiveness of Equine-Assisted Activities and Therapies for Improving Adaptive Behavior and Motor Function in Autism Spectrum Disorder. 2021. Leonardo Zoccante, et al., J Clin Med. | Duplication |
| Animal Farm in healthcare: definitions, policies, laws and implications for health professionals. 2021. Ben-Sefer, E., Shields, L. Journal of the Royal Society of Medicine. | Not AAI |
| Effects of a Therapeutic Horseback Riding Program on Social Interaction and Communication in Children with Autism. 2021. Mengxian Zhao, et al., Int J Environ Res Public Health. | Duplication |
| The Process of Integrating Animal-Assisted Therapy into Clinical Social Work Practice. 2021. Compitus, K., et al.,  Clinical Social Work Journal. | Perspectives |
| The role of hippotherapy in children rehabilitation \| Hipoterapia w rehabilitacji dzieci. 2021. Kłoda, M., et al., Fizjoterapia Polska. | Review |
| Dog training alleviates PTSD symptomatology by emotional and attentional regulation. 2021. Inon Maoz, et al., Eur J Psychotraumatol. | Duplication |
| Hippotherapy and life habits with children with motor deficit and neurodevelopmental impairment: A pilot survey of parents. 2021. Alex Potvin-Bélanger, et al., J Pediatr Rehabil Med. | Duplication |
| Caregiver perceptions of the benefits of hippotherapy for children with various disorders, disabilities, and medical conditions. 2021. Scotland-Coogan, D., et al., Journal of Human Behavior in the Social Environment. | Perspectives |
| "The connection just happens": Therapists' perspectives of canine-assisted occupational therapy for children on the autism spectrum. 2020. Jessica Rachel Hill, et al., Aust Occup Ther J. | Duplication |
| Animal Assisted Therapy for Children and Adolescents with Autism Spectrum Disorder: Parent perspectives. 2020. Maeve Doyle London, Lynette Mackenzie, Meryl Lovarini, Claire Dickson, Alberto Alvarez-Campos. | Duplication |
| Canine Assisted Occupational Therapy for Children on the Autism Spectrum: A Pilot Randomised Control Trial. 2020. Jessica Hill, et al., J Autism Dev Disord. | Duplication |
| Canine-assisted occupational therapy for children on the autism spectrum: Parents' perspectives. 2020. Jessica Rachel Hill, et al., Aust Occup Ther J. | Duplication |
| Implementation of Assisted Therapy With Dogs in the Therapeutic Approach to People With Autistic Spectrum Disorder. 2020. Isabel Morales-Moreno, et al., Holist Nurs Pract. | Duplication |
| Social Development of Adults with Autism Spectrum Disorder During Dog-Assisted Therapy: A Detailed Observational Analysis. 2020. Carolien Wijker, et al., Int J Environ Res Public Health. | Duplication |
| Exploring Human–Companion Animal Interaction in Families of Children with Autism. 2020. Carlisle, G.K., et al., Journal of Autism and Developmental Disorders. | Not intervention |
| Perspectives of Former Students with ASD from Australia and New Zealand on Their University Experience. 2020. Anderson, A.H., et al., Journal of Autism and Developmental Disorders. | Perspectives |
| Parental Perspectives of Occupational Therapy in an Equine Environment for Children with Autism Spectrum Disorder. 2020. Dorothy Kalmbach, et al., Occup Ther Health Care. | Duplication |
| Pilot Study: Occupational Therapy in an Equine Environment for Youth With Autism. 2020. B Caitlin Peters, et al. OTJR (Thorofare N J). | Duplication |
| Improving social participation of children with autism spectrum disorder: Pilot testing of an early animal-assisted intervention in Spain. 2020. Adriana Ávila-Álvarez, et al., Health Soc Care Community. | Duplication |
| Evaluating preference for and reinforcing efficacy of a therapy dog to increase verbal statements. 2020. Courtney D Jorgenson, et al., J Appl Behav Anal. | Duplication |
| Canine-Assisted Therapy for Children with Autism Spectrum Disorder: a Systematic Review. 2020. Hardy, K.K., Weston, R.N. Review Journal of Autism and Developmental Disorders. | Systematic Review |
| Effects of Dog Assisted Therapy for Adults with Autism Spectrum Disorder: An Exploratory Randomized Controlled Trial. 2020. Carolien Wijker, et al., J Autism Dev Disord. | Duplication |
| Changes in behavioural synchrony during dog-assisted therapy for children with autism spectrum disorder and children with Down syndrome. 2020. Richard Eric Griffioen, et al., J Appl Res Intellect Disabil. | Duplication |
| Comparison of contingent and noncontingent access to therapy dogs during academic tasks in children with autism spectrum disorder. 2020. Alexandra Protopopova, et al., J Appl Behav Anal. | Duplication |
| Canine-assisted occupational therapy for children on the autism spectrum: Challenges in practice. 2020. Hill, J., et al., British Journal of Occupational Therapy. | Perspectives |
| Introducing animal-assisted intervention for special education in integrated farming system. 2020. Zhi, T.X., et al., IAFOR Journal of Education. | Perspectives |
| Donkey therapy and hippotherapy: Two faces of the same coin? 2020. Portaro, S., et al., Innovations in Clinical Neuroscience. | Perspectives |
| Human-Animal Interaction Research: Progress and Possibilities. 2019. Griffin, J.A., et al., Frontiers in Psychology. | Perspectives |
| Process Evaluation of Animal-Assisted Therapy: Feasibility and Relevance of a Dog-Assisted Therapy Program in Adults with Autism Spectrum Disorder. 2019. Carolien Wijker, et al., Animals (Basel). | Duplication |
| Vitality from Experiences in Nature and Contact with Animals-A Way to Develop Joint Attention and Social Engagement in Children with Autism? 2019. Kristina Byström, et al., Int J Environ Res Public Health. | Duplication |
| An umbrella review of the evidence for equine-assisted interventions. 2019. Stern, C., Chur-Hansen, A. Australian Journal of Psychology. | Umbrella review |
| Verbal Interactional Synchronization between Therapist and Children with Autism Spectrum Disorder during Dolphin Assisted Therapy: Five Case Studies. 2019. Richard Griffioen, et al., Animals (Basel). | Duplication |
| Animal-assisted activity improves social behaviors in psychiatrically hospitalized youth with autism. 2019. Monique M Germone, et al., Autism. | Duplication |
| Equine-Assisted Interventions for Psychosocial Functioning in Children and Adolescents with Autism Spectrum Disorder: a Literature Review. 2019. Tan, V.X.-L., Simmonds, J.G. Review Journal of Autism and Developmental Disorders. | Literature Review |
| Evaluating Animal-Assisted Interventions: An Empirical Illustration of Differences between Outcome Measures. 2019. Steffie van der Steen, et al., Animals (Basel). DOI: 10.3390/ani9090645. | Duplication |
| Animal-Assisted Activity for Children with Autism Spectrum Disorder: Parents' and Therapists' Perception. 2019. Ana L L Michelotto. J Altern Complement Med. | Duplication |
| Do Animals Perceive Human Developmental Disabilities? Guinea Pigs' Behaviour with Children with Autism Spectrum Disorders and Children with Typical Development. A Pilot Study. 2019. Marine Grandgeorge, et al., Animals (Basel). | Duplication |
| A therapy beyond. 2019. Reid, A.S. Irish Journal of Psychological Medicine. | Not for ASD |
| Comments on the dilemma in the April issue: Enrolling in animal-assisted therapy programmes. 2019. McCulloch, S. In Practice. | Comments |
| Equine Assisted Therapy for Patients with Post Traumatic Stress Disorder: A Case Series Study. 2019. Assaf Shelef, et al., Mil Med. | Duplication |
| Animal-assisted interventions for treatment of childhood psychiatric disorders. 2019. Shotwell, J.S., Wagner, K.D. Psychiatric Annals | Psychiatric disorders |
| Can Canine-Assisted Interventions Affect the Social Behaviours of Children on the Autism Spectrum? A Systematic Review. 2019. Hill, J., et al., Review Journal of Autism and Developmental Disorders. | Systematic Review |
| Complementary and alternative medicine use in adults with autism spectrum disorder in Germany: results from a multi-center survey. 2019. Juliana Höfe, et al., BMC Psychiatry. | Duplication |
| Equine assisted activities and therapies in children with autism spectrum disorder: A systematic review and a meta-analysis. 2019. Trzmiel, T., et al., Complementary Therapies in Medicine. | Duplication |
| Living and Robotic Dogs as Elicitors of Social Communication Behavior and Regulated Emotional Responding in Individuals with Autism and Severe Language Delay: A Preliminary Comparative Study. 2019. Silva, K., et al., Anthrozoos. | Severe Language Delay |
| Effect of Motorized Elephant-Assisted Therapy Program on Balance Control of Children with Autism Spectrum Disorder. 2019. Satiansukpong Nuntanee, Sasat Daranee. Occup Ther Int. | Duplication |
| Animal assisted therapy: Systematic review of literature. 2019. Mandrá, P.P., et al., CODAS. | Systematic review |
| Reliability and Validity Assessment of the Observation of Human-Animal Interaction for Research (OHAIRE) Behavior Coding Tool. 2018. Noémie A Guérin, et al., Front Vet Sci. | Duplication |
| Effectiveness of Animal-Assisted Therapy in the Pediatric Population: Systematic Review and Meta-Analysis of Controlled Studies. 2018. Jesús David Charry-Sánchez, et al., J Dev Behav Pediatr. | Duplication |
| Long-Term Effect of Therapeutic Horseback Riding in Youth With Autism Spectrum Disorder: A Randomized Trial. 2018. Robin L Gabriels, et al., Front Vet Sci. | Duplication |
| Editorial: Children and Companion Animals: Psychosocial, Medical and Neurobiological Implications. 2018. Andrea Beetz, et al., Front Vet Sci. | Duplication |
| Effects of Equine Therapy on Individuals with Autism Spectrum Disorder: A Systematic Review. 2018. Sudha M Srinivasan, et al., Rev J Autism Dev Disord. | Duplication |
| Horseback riding therapy for a deafblind individual enabled by a haptic interface. 2018. Matjaž Ogrinc, e al., Assist Technol. | Duplication |
| 'It just opens up their world': autism, empathy, and the therapeutic effects of equine interactions. 2018. Roslyn Malcolm, et al., Anthropol Med. | Duplication |
| Companion animals and human health: benefits, challenges, and the road ahead for human-animal interaction. E Friedman, C A Krause-Parello. 2018. Rev Sci Tech. | Duplication |
| Can Dogs Assist Children with Severe Autism Spectrum Disorder in Complying with Challenging Demands? An Exploratory Experiment with a Live and a Robotic Dog. 2018. Karine Silva, et al., J Altern Complement Med. DOI: 10.1089/acm.2017.0254. | Duplication |
| Parent Perceptions of Psychosocial Outcomes of Equine-Assisted Interventions for Children with Autism Spectrum Disorder. 2018. Vanessa Xue-Ling Tan, Janette Graetz Simmonds. J Autism Dev Disord. DOI: 10.1007/s10803-017-3399-3. | Duplication |
| Dance and equine-assisted therapy in autism spectrum disorder: Crossover randomized clinical trial. 2018. Souza-Santos, C. Clinical Neuropsychiatry. | Multiple interventions |
| The effect of swimming with dolphins on the selected balance and strength gross motor skills of eight-year-old children with autism spectrum disorder. 2018. Ashtari, M., Sheikh, M. Sport Science. | Not English |
| Evaluation of the effectiveness of canine assisted therapy as a complementary method of rehabilitation in disabled children. 2018. Grabowska, I., Ostrowska, B. Physiotherapy Quarterly. | Perspectives |
| PET-therapy as an innovative intervention tool in the autism spectrum disorder motor deficits. 2018. De Vita, T., Rosa, R., Napolitano, F. Acta Medica Mediterrane. | Perspectives |
| The “Biodanza SRT” proposal in neurodegenerative diseases. 2018. Rosa, R., Acta Medica Mediterranea. | Perspectives |
| Reflections on Recent Research Into Animal-Assisted Interventions in the Military and Beyond. 2017. Christina B Rumayor, Amy M Thrasher. Curr Psychiatry Rep. DOI: 10.1007/s11920-017-0861-z. | Duplication |
| Social rivalry triggers visual attention in children with autism spectrum disorders. 2017. Marine Grandgeorge, et al., Sci Rep. DOI: 10.1038/s41598-017-09745-6. | Duplication |
| Advances in Mental Health Care: Five N = 1 Studies on the Effects of the Robot Seal Paro in Adults With Severe Intellectual Disabilities. 2017. Wagemaker, E., Journal of Mental Health Research in Intellectual Disabilities. | Not for ASD |
| Autism and Equine-Assisted Interventions: A Systematic Mapping Review. 2017. B Caitlin McDaniel Peters, Wendy Wood. J Autism Dev Disord. DOI: 10.1007/s10803-017-3219-9 | Duplication |
| Equine assisted psychotherapy for 3 children with Smith-Magenis syndrome: Effects on their frustration intolerance and other benefits \| Effets de l’équithérapie pour 3 enfants présentant le syndrome Smith-Magenis: la tolérance à la frustration et autres apports. 2017. Le Hénaff, I., Grandgeorge, M. Neuropsychiatrie de l'Enfance et de l'Adolescence. | Case |
| Feline-assisted therapy: Integrating contact with cats into treatment plans. 2017. Tomaszewska, K., Polish Annals of Medicine. | Perspectives |
| The Impact of a Horse Riding Intervention on the Social Functioning of Children with Autism Spectrum Disorder. 2017. Androulla Harris, Joanne M Williams. Int J Environ Res Public Health. DOI: 10.3390/ijerph14070776. | Duplication |
| Research on animal-assisted intervention and autism spectrum disorder, 2012-2015. 2017. Marguerite O'Haire. Appl Dev Sci. DOI: 10.1080/10888691.2016.1243988. | Duplication |
| Introduction to a thematic series on animal assisted interventions in special populations. 2017. McCune, S., Applied Developmental Science. | Review |
| Assessing Preferences for Animals in Children with Autism: A New Use for Video-Based Preference Assessment. 2017. Noémie A Guérin, et al., Front Vet Sci. DOI: 10.3389/fvets.2017.00029. | Duplication |
| Therapeutic Horseback Riding Crossover Effects of Attachment Behaviors with Family Pets in a Sample of Children with Autism Spectrum Disorder. 2017. Jessie D Petty, et al., Int J Environ Res Public Health. DOI: 10.3390/ijerph14030256. | Duplication |
| Attitudes to and beliefs about animal assisted therapy for children with disabilities. 2017. Esther Yap, et al., Complement Ther Clin Pract. DOI: 10.1016/j.ctcp.2016.11.009. | Duplication |
| Animal-Assisted Therapies for Youth with or at risk for Mental Health Problems: A Systematic Review. 2017. Kimberly Eaton Hoagwood, et al., Appl Dev Sci. DOI: 10.1080/10888691.2015.1134267. | Duplication |
| What effect does participating in an assistance dog program have on the quality of life of children with Autism Spectrum Disorders and their caregivers? A systematic review of current literature. 2017. Sprod, E., Norwood, M.F.  Journal of Social Inclusion | Systematic review |
| Complementary and alternative medicine use in children with autistic spectrum disorder in Mauritius. 2017. Mahomed, S., Mahomoodally, F. Journal of Intercultural Ethnopharmacology | Perspectives |
| Equine-Assisted Therapy for Children with Autism Spectrum Disorder: a Comprehensive Literature Review. 2016. Mapes, A.R., Rosén, L.A. Review Journal of Autism and Developmental Disorders | Review |
| Equine-Assisted Intervention in a child diagnosed with autism spectrum disorder: a case report. 2016. Stefania Cerino, et al., Riv Psichiatr. DOI: 10.1708/2596.26730. | Duplication |
| Equine-Assisted Occupational Therapy: Increasing Engagement for Children With Autism Spectrum Disorder. 2016. Cecilia Llambias, et al., Am J Occup Ther. DOI: 10.5014/ajot.2016.020701. | Duplication |
| Brief Report: The Effects of Equine-Assisted Activities on the Social Functioning in Children and Adolescents with Autism Spectrum Disorder. 2016. Sophie Anderson, Kerstin Meints. J Autism Dev Disord. DOI: 10.1007/s10803-016-2869-3. | Duplication |
| Improvement in symptoms of autism spectrum disorder in children with the use of gastrin-releasing peptide: An open trial. 2016. Becker, M.M., Clinical Neuropharmacology. | Not pet intervention |
| Using Animal-assisted Therapy to Enrich Psychotherapy. 2016. Jeanne Louise Amerine, Grace B Hubbard. Adv Mind Body Med. PMID: 27541053. | Duplication |
| The Transformative Power of the Dog: The Growing Use of Canine Assistants in Therapeutic Interventions and School Settings. 2016. Finn-Stevenson, M. Journal of the American Academy of Child and Adolescent Psychiatry, | Perspectives |
| Integrative approaches to caring for children with autism. 2016. Klein, N., Kemper, K.J. Current Problems in Pediatric and Adolescent Health Care | Multiple intervention |
| Hippotherapy acute impact on heart rate variability non-linear dynamics in neurological disorders. 2016. Cabiddu, R., Physiology and Behavior. | Not for ASD |
| The long-term benefits of dog ownership in families with children with autism. 2016. Hall, S.S., et al., Journal of Veterinary Behavior. | Perspectives |
| What Factors Are Associated with Positive Effects of Dog Ownership in Families with Children with Autism Spectrum Disorder? The Development of the Lincoln Autism Pet Dog Impact Scale. 2016. Sophie Susannah Hall, et al., PLoS One. DOI: 10.1371/journal.pone.0149736. | Duplication |
| Equine-assisted therapy as intervention for motor proficiency in children with autism spectrum disorder: Case studies. 2016. De Milander, M., et al., South African Journal for Research in Sport, Physical Education and Recreation | Case |
| Additional Evidence is Needed to Recommend Acquiring a Dog to Families of Children with Autism Spectrum Disorder: A Response to Wright and Colleagues. 2016. Crossman, M.K., Kazdin, A.E. Journal of Autism and Developmental Disorders | Letter |
| Additional Evidence is Needed to Recommend Acquiring a Dog to Families of Children with Autism Spectrum Disorder: A Response to Crossman and Kazdin. 2016. Wright, H.F., Hall, S., Mills, D.S. Journal of Autism and Developmental Disorders | Letter |
| Effectiveness of a Standardized Equine-Assisted Therapy Program for Children with Autism Spectrum Disorder. 2016. Marta Borgi, et al., J Autism Dev Disord. DOI: 10.1007/s10803-015-2530-6. | Duplication |
| Prevalence and correlates of use of complementary and alternative medicine in children with autism spectrum disorder in Europe. 2015. Salomone, E., et al., European Journal of Pediatrics. | Multiple intervention |
| Animal assisted interventions for children with autism spectrum disorder: A systematic review. 2015. Davis, T.N., et al., Education and Training in Autism and Developmental Disabilities. | Systematic review |
| Effects of therapeutic horse riding on gait cycle parameters and some aspects of behavior of children with autism. 2015. H Steiner, Zs Kertesz. Acta Physiol Hung. DOI: 10.1556/036.102.2015.3.10. | Duplication |
| Pet ownership and physical health. 2015. Matchock, R.L. Current Opinion in Psychiatry | Not for ASD |
| Assessing the benefits and risks of owning a pet. 2015. Cherniack, E.P., Cherniack, A.R. CMAJ | Not intervention |
| Equine-Facilitated Psychotherapy With Children and Adolescents: An Update and Literature Review. 2015. Lentini, J.A., Knox, M.S. Journal of Creativity in Mental Health | Review |
| Randomized Controlled Trial of Therapeutic Horseback Riding in Children and Adolescents With Autism Spectrum Disorder. 2015. Robin L Gabriels, Zhaoxing Pan, Briar Dechant, John A Agnew, Natalie Brim, Gary Mesibov. J Am Acad Child Adolesc Psychiatry. DOI: 10.1016/j.jaac.2015.04.007. | Duplication |
| "But-He'll Fall!": Children with Autism, Interspecies Intersubjectivity, and the Problem of 'Being Social'. 2015. Olga Solomon. Cult Med Psychiatry. DOI: 10.1007/s11013-015-9446-7. | Duplication |
| Increasing the Social Communication of a Boy With Autism Using Animal-assisted Play Therapy: A Case Report. 2015. Suk Chun Fung. Adv Mind Body Med. PMID: 26026154. | Duplication |
| Autism spectrum disorder and pet therapy. 2015. Caitlin M Siewertsen, et al., Adv Mind Body Med. PMID: 25831431. | Duplication |
| A systematic review of randomized controlled trials of animal-assisted therapy on psychosocial outcomes. 2015. Maujean, A., Pepping, C.A., Kendall, E. Anthrozoos | Review |
| Farming for health: Contexts and benefits of Pet-therapy. 2015. Rapisarda, P., De Pasquale, C., Greco, C. Quality - Access to Success | Perspectives |
| Complementary and Alternative Therapies for Autism Spectrum Disorder. 2015. Brondino, N., et al., Evidence-based Complementary and Alternative Medicine. | Review |
| Complementary and Alternative Medicine Treatments for Children with Autism Spectrum Disorders. 2015. Levy, S.E., Hyman, S.L. Child and Adolescent Psychiatric Clinics of North America | Review |
| The short-term effects of transcranial direct current stimulation on electroencephalography in children with autism: A randomized crossover controlled trial. 2015. Amatachaya, A., et al., Behavioural Neurology. | Not AAT |
| Effects of equine interaction on EEG asymmetry in children with autism spectrum disorder: A pilot study. 2015. Chen, C.-C., et al. International Journal of Developmental Disabilities. | Not AAT |
| Autism: Cause factors, early diagnosis and therapies. 2014. Bhat, S., et al., Reviews in the Neurosciences. | Not AAT |
| Setting the One Health agenda and the human-companion animal bond. 2014. Gregg K Takashima, Michael J Day. Int J Environ Res Public Health. DOI: 10.3390/ijerph111111110. | Duplication |
| Effects of equine assisted activities on autism spectrum disorder. 2014. Lanning, B.A., et al., Journal of Autism and Developmental Disorders. | Duplication |
| Animal-assisted Activities for Students With Disabilities: Obtaining Stakeholders' Approval and Planning Strategies for Teachers. 2014. Baumgartner, E., Cho, J.-I. Childhood Education | Not AAT |
| The life-changing power of the horse: Equine-assisted activities and therapies in the U.S. 2014. Berg, E.L., Causey, A. Animal Frontiers | Not AAT |
| Effects of classroom animal-assisted activities on social functioning in children with autism spectrum disorder. 2014. Marguerite E O'Haire, et al., J Altern Complement Med. DOI: 10.1089/acm.2013.0165. | Duplication |
| Brief report: The smiles of a child with autism spectrum disorder during an animal-assisted activity may facilitate social positive behaviors - Quantitative analysis with smile-detecting interface. 2014. Funahashi, A., et al., Journal of Autism and Developmental Disorders. | Case |
| Attenuated adenylosuccinate lyase deficiency: A report of one case and a review of the literature. 2014. Jurecka, A., et al., Neuropediatrics. | Review |
| Parents' perspectives on the value of assistance dogs for children with autism spectrum disorder: a cross-sectional study. 2014. Louise Burgoyne, et al., BMJ Open. DOI: 10.1136/bmjopen-2014-004786. | Perspectives |
| Could 'Rx: Pet therapy' come back to bite you? Mossman, D. Current Psychiatry | Perspectives |
| Brief report: The smiles of a child with autism spectrum disorder during an animal-assisted activity may facilitate social positive behaviors - Quantitative analysis with smile-detecting interface. 2014. Funahashi, A., et al., Journal of Autism and Developmental Disorders. | Duplication |
| Interaction with a therapy dog enhances the effects of social story method in autistic children. 2014. Grigore, A.A., Rusu, A.S. Society and Animals | Case |
| The value of (research on) animals in children's lives: 2014. Severson, R.L. Human Development | Perspectives |
| The 'autism in school age: Early diagnosis for treatment \| Autizam u školskom uzrastu: Rano dijagnosticiranje za tretman. 2013. Perrotta, F., Altavilla, G. Sport Science | Perspectives |
| Effect of hippotherapy on motor control, adaptive behaviors, and participation in children with autism spectrum disorder: a pilot study. 2013. Heather F Ajzenman, et al., Am J Occup Ther. DOI: 10.5014/ajot.2013.008383. | Duplication |
| The association between therapeutic horseback riding and the social communication and sensory reactions of children with autism. 2013. Sandra C Ward, et al., J Autism Dev Disord. DOI: 10.1007/s10803-013-1773-3. | Duplication |
| Animal-assisted intervention for autism spectrum disorder: a systematic literature review. 2013. Marguerite E O'Haire. J Autism Dev Disord. DOI: 10.1007/s10803-012-1707-5. | Duplication |
| Design and development of a Virtual Dolphinarium for children with autism. 2013. Yiyu Cai, et al., IEEE Trans Neural Syst Rehabil Eng. DOI: 10.1109/TNSRE.2013.2240700. | Duplication |
| Our canine carers. 2013. Seggie, J. South African Medical Journal | Perspectives |
| Use of assistance and therapy dogs for children with autism spectrum disorders: a critical review of the current evidence. 2013. Alessandra Berry, et al., J Altern Complement Med. DOI: 10.1089/acm.2011.0835. | Duplication |
| An hypothesis about jung's collective unconscious and animal-assisted therapy. 2013. Carminati, G.G., et al., NeuroQuantology | Duplication |
| Does pet arrival trigger prosocial behaviors in individuals with autism? 2012. Marine Grandgeorge, et al., PLoS One. DOI: 10.1371/journal.pone.0041739. | Duplication |
| Care farms as a short-break service for children with Autism Spectrum Disorders. 2012. Ferwerda-Van Zonneveld, et al., NJAS Wageningen Journal of Life Sciences. | Not AAT |
| Animal-assisted interventions as innovative tools for mental health. 2011. Cirulli, F., et al., Annali dell'Istituto Superiore di Sanita. | Perspectives |
| The influence of animals on the development of children. 2011. Nienke Endenburg, Hein A van Lith. Vet J. DOI: 10.1016/j.tvjl.2010.11.020. | Duplication |
| Equine-assisted therapy. 2011. Maclean, B. Journal of Rehabilitation Research and Development | Perspectives |
| Can dogs prime autistic children for therapy? Evidence from a single case study. 2011. Karine Silva, et al., J Altern Complement Med. DOI: 10.1089/acm.2010.0436. | Duplication |
| Genetic evaluation of the pediatric patient with hypotonia: Perspective from a hypotonia specialty clinic and review of the literature. 2011. Lisi, E.C., Cohn, R.D. Developmental Medicine and Child Neurology | Review |
| Animal-assisted interventions in internal and rehabilitation medicine: a review of the recent literature. 2011. S Muñoz Lasa, et al., Panminerva Med. PMID: 21659977. | Duplication |
| Prospective trial of equine-assisted activities in autism spectrum disorder. 2011. Janet K Kern, et al., Altern Ther Health Med. PMID: 22164808. | Duplication |
| Lost in a lost world. 2011. William Benda. ltern Ther Health Med. PMID: 22164807. | Duplication |
| Behavioral intervention for domestic pet mistreatment in a young child with autism. 2011. Bergstrom, R., Tarbox, J., Gutshall, K.A. Research in Autism Spectrum Disorders | Not AAT |
| Kynotherapy as a complement of the rehabilitation process in people with developmental deficiencies \| Kynoterapia jako uzupetnienie procesu rehabilitacji osób z deficytami rozwojowymi. 2010. Broszkiewicz, P. Fizjoterapia. | Review |
| Use of complementary and alternative medicine (CAM) treatments by parents of children with autism spectrum disorders. 2010. Christon, L.M., Mackintosh, V.H., Myers, B.J. Research in Autism Spectrum Disorders | Perspectives |
| What a dog can do: Children with autism and therapy dogs in social interaction. 2010. Keino, H., et al., Journal of Equine Science | Review |
| Autistic rider. 2009. Carl A Hammerschlag. PMID: 19772030 | Duplication |
| Dolphin Assisted Therapy: Can swimming with dolphins be a suitable treatment? 2008. Williamson, C. Developmental Medicine and Child Neurology | Not available |
| Play with online virtual pets as a method to improve mirror neuron and real world functioning in autistic children. 2008. Eric Lewin Altschuler. Med Hypotheses. DOI: 10.1016/j.mehy.2007.07.030. | Duplication |
| Animal-assisted therapy: A meta-analysis. 2007. Nimer, J, Lundahl, B. Anthrozoos | Meta-analysis |
| Alternative therapeutic intervention for individuals with Rett syndrome. 2007. Meir Lotan. ScientificWorldJournal. DOI: 10.1100/tsw.2007.4. | Duplication |
| Rett syndrome. A review with emphasis on clinical characteristics and intervention. 2006. Lotan, M., Ben-Zeev, B. The Scientific World Journal | Review |
| No dogs. Guide dogs by prior permission. 2006. Pease, J., Brown, A. Psychiatric Bulletin | Not AAT |
| The effectiveness of interventions for children with autism. 2005. Howlin, P. Journal of Neural Transmission, Supplement | Review |
| Analysis of child-dog play behavior in child psychiatry. 2005. Prothmann, A, et al., Anthrozoos | Review |
| Pet therapy: Animals in human therapy \| Pet therapy: Gli animali nella terapia umana. 2003. Ballarini, G. Acta Biomedica de l'Ateneo Parmense | Not for ASD |
| Animal-assisted therapy for children with pervasive developmental disorders. 2002. Martin, F., Farnum, J. Western Journal of Nursing Research  The effects of equine-assisted therapy in improving the psychosocial functioning of children with autism. 2010. Memishevikj, H., Hodzhikj, S. Journal of Special Education and Rehabilitation. | Not for ASD  Russian |
|  |  |
| Google scholar |  |
| Efectos de un Programa de Equitación Adaptada y Terapéutica en un Grupo de Niños con Trastornos del Espectro Autista. 2014. Andrés García-Gómez, et al., INVESTIGACIÓN APLICADA, ACADÉMICA Y/O PROFESIONAL. | Spanish |
| Efectos de un Programa de Equitación Adaptada y Terapéutica en un Grupo de Niños con Trastornos del Espectro Autista. 2014. Andrés García-Gómez, et al., | Spanish |
|  |  |
|  |  |

**Appendix 4. List of systematic review or meta-analysis**

| Systematic review and meta-analysis |
| --- |
| Autism and Equine-Assisted Interventions: A Systematic Mapping Review. 2017. B Caitlin McDaniel Peters, Wendy Wood. J Autism Dev Disord. DOI: 10.1007/s10803-017-3219-9  Animal-assisted intervention for autism spectrum disorder: a systematic literature review. 2013. Marguerite E O'Haire. J Autism Dev Disord. DOI: 10.1007/s10803-012-1707-5.  Effects of Equine Therapy on Individuals with Autism Spectrum Disorder: A Systematic Review. 2018. Sudha M Srinivasan, et al., Rev J Autism Dev Disord. DOI: 10.1007/s40489-018-0130-z.  Equine assisted activities and therapies in children with autism spectrum disorder: A systematic review and a meta-analysis. 2019. Tomasz Trzmiel, et al., Complement Ther Med. DOI: 10.1016/j.ctim.2018.11.004.  Effectiveness of Animal-Assisted Therapy in the Pediatric Population: Systematic Review and Meta-Analysis of Controlled Studies. 2018. Jesús David Charry-Sánchez, et al., J Dev Behav Pediatr. DOI: 10.1097/DBP.0000000000000594.  Effects of Therapeutic Horseback-Riding Program on Social and Communication Skills in Children with Autism Spectrum Disorder: A Systematic Review and Meta-Analysis. 2022. Shihui Chen, et al., Int J Environ Res Public Health. DOI: 10.3390/ijerph192114449.  Effects of Equine-Assisted Activities and Therapies for Individuals with Autism Spectrum Disorder: Systematic Review and Meta-Analysis. 2023. Ningkun Xiao, et al., Int J Environ Res Public Health. DOI: 10.3390/ijerph20032630.  Calm with horses? A systematic review of animal-assisted interventions for improving social functioning in children with autism. 2022. Jon H Sissons, et al., Autism. DOI: 10.1177/13623613221085338.  Equine assisted services impact on social skills in autism spectrum disorder: A meta-analysis. 2023. Jérémy Madigand, et al., Prog Neuropsychopharmacol Biol Psychiatry. DOI: 10.1016/j.pnpbp.2023.110765.  Animal-Assisted Interventions for School-Aged Children with Autism Spectrum Disorder: A Meta-Analysis. 2021. Mirena Dimolareva, Thomas J Dunn. J Autism Dev Disord. DOI: 10.1007/s10803-020-04715-w.  The effectiveness of animal-assisted therapy for children and adolescents with autism spectrum disorder: A systematic review. 2023. Amy Kate Rehn, et al., Complement Ther Clin Pract. DOI: 10.1016/j.ctcp.2022.101719. |
| Animal Interaction Affecting Core Deficit Domains Among Children with Autism: A Meta-Analysis. 2021. Michael J Droboniku, Matthew P Mychailyszyn. J Autism Dev Disord. DOI: 10.1007/s10803-021-04891-3. |
| Animal-Assisted Interventions for Autism Spectrum Disorder: A Systematic Review of the Literature from 2016 to 2020. 2023. Leanne O Nieforth, et al., Rev J Autism Dev Disord. DOI: 10.1007/s40489-021-00291-6. |
| The efficacy of executive function interventions in children with autism spectrum disorder: a systematic review and meta-analysis. 2022. Cavalli, G., et al., Expert Review of Neurotherapeutics.  Canine-Assisted Therapy for Children with Autism Spectrum Disorder: a Systematic Review. 2020. Hardy, K.K., Weston, R.N. Review Journal of Autism and Developmental Disorders.  Can Canine-Assisted Interventions Affect the Social Behaviours of Children on the Autism Spectrum? A Systematic Review. 2019. Hill, J., et al., Review Journal of Autism and Developmental Disorders.  Animal assisted interventions for children with autism spectrum disorder: A systematic review. 2015. Davis, T.N., et al., Education and Training in Autism and Developmental Disabilities. |

**Appendix 5. Major modifications for protocol in PROSPERO (CRD42023431425)**

| **Registered** | **Deviation** |
| --- | --- |
| Our protocol : Efficacy of animal-assisted therapy for autism spectrum disorder: an umbrella review | Changed : Efficacy of animal-assisted intervention for autism spectrum disorder: systematic review and meta-analysis |
| Our protocol specified that: *‘In this study, we will analyze the effectiveness of animal-assisted therapy for autism spectrum disorders. We will also compare the effects of common animal-assisted treatments, such as equine-assisted therapy and canine-assisted therapy, on the effectiveness of ASD.’* | Unfortunately; The outcome criteria were very different across the trials. Therefore, we were unable to conduct these analyses. |
| Our protocol specified that: ‘*We will conduct a comprehensive search in three databases: Scopus, MEDLINE, and PubMed, with language restrictions to English and article types of meta-analysis or systematic review.’* | We search in Scopus, Pubmed, google scholar, and screened additional systematic review and meta-analysis. |
| Our protocol specified that: ‘*Animal-assisted therapy: common adjuvant therapy animals include horses, dogs, and dolphins. In addition, we will also include adjuvant therapy performed by rabbits, dolphins, guinea pigs, and American camels.’* | In our study, we not just included anmial-assisted therapy, also included animal-assisted activities. |

**Appendix 6. RoB Quality for RCTs**

| First Author, Publish year | Random sequence generation | Allocation concealment | Blinding of participants and personnel | Blinding of outcome assessment | Incomplete outcome data | Selective reporting | |  |
| --- | --- | --- | --- | --- | --- | --- | --- | --- |
|  |  |  |  |  |  |  |  |  |
|  |  |  |  |  |  |  |  |  |
| B Caitlin Peters, 2022 | Y | (-) | N | Y | N | N |  |  |
| Zhao, M, 2022 | Y | Y | Y | (-) | N | N |  |  |
| Mengxian Zhao, 2021 | (-) | Y | (-) | N | N | N |  |  |
| Carolien Wijker, 2021 | Y | (-) | N | N | N | N |  |  |
| Peters, B.C., 2021 | Y | N | N | N | N | N |  |  |
| Hernández-Espeso, 2021 | Y | Y | N | N | N | N |  |  |
| Jessica Hill, 2020 | Y | Y | N | Y | N | N |  |  |
| Carolien Wijker, 2020 | Y | Y | N | Y | N | N |  |  |
| Ozyurt, Gonca., 2020 | (-) | Y | (-) | Y | N | Y |  |  |
| Satiansukpong Nuntanee, 2019 | N | N | (-) | N | N | N |  |  |
| Kwon, S., 2019 | N | (-) | (-) | N | Y | N |  |  |
| Robin L Gabriels, 2018 | Y | Y | N | N | Y | N |  |  |
| Pan, Z., 2018 | Y | (-) | (-) | Y | N | N |  |  |
| Androulla Harris, 2017 | N | N | N | N | N | N |  |  |
| Marta Borgi, 2016 | Y | Y | N | N | N | Y |  |  |
| Robin L Gabriels， 2015 | Y | Y | Y | N | N | N |  |  |
| H Steiner, 2015 | Y | Y | N | N | Y | N |  |  |
| Beth A Lanning, 2014 | N | N | N | N | (-) | Y |  |  |
| Fung, S.-C., 2014 | Y | N | N | (-) | N | N |  |  |
| Bass, M. M., 2009 | Y | (-) | (-) | (-) | N | N |  |  |

**Appendix 7. 6. ROBINS-I for non-RCTs**

| First author, year | | | | | | | Rezapour-Nasrabad, R.R, 2022 | | | | Abadi, M.R.H., 2022 | | | | Leonardo Zoccante, 2021 | | | | B Caitlin Peters, 2020 | | | | Adriana Ávila-Álvarez, 2020 | | | | Isabel Morales-Moreno, 2020 | | | | Portela-Pino, I.,  2020 | | | | Kalmbach, D.， 2020 | | | | Monique M Germone, 2019 | | | | Ana L L Michelotto， 2019 | | | | Tan, V. X., 2018 | | | | Cecilia Llambias, 2016 | | | | Sophie Anderson, 2016 | | | | Margo B Holm, 2014 | | | | Sandra C Ward, 2013 | | | | Ghorban, Hemati.， 2013 | | | | Jenkins, Sarah R., 2013 | | | | Heather F Ajzenman, 2013 | | | | Emílio Salgueiro, 2012 | | | | Tabares, C., 2012 | | | | Gabriels, Robin L., 2012 | | | | MdYusof,2012 | | | | Janet K Kern, 2011 | | | | Robert Viau, 2010 | | | | Taylor, Renee R., 2009 | | | |  |  |
| --- | --- | --- | --- | --- | --- | --- | --- | --- | --- | --- | --- | --- | --- | --- | --- | --- | --- | --- | --- | --- | --- | --- | --- | --- | --- | --- | --- | --- | --- | --- | --- | --- | --- | --- | --- | --- | --- | --- | --- | --- | --- | --- | --- | --- | --- | --- | --- | --- | --- | --- | --- | --- | --- | --- | --- | --- | --- | --- | --- | --- | --- | --- | --- | --- | --- | --- | --- | --- | --- | --- | --- | --- | --- | --- | --- | --- | --- | --- | --- | --- | --- | --- | --- | --- | --- | --- | --- | --- | --- | --- | --- | --- | --- | --- | --- | --- | --- | --- | --- | --- | --- | --- | --- | --- | --- | --- | --- | --- |
| Signalling questions | | | | | | | Response options | | | | Response options | | | | Response options | | | | Response options | | | | Response options | | | | Response options | | | | Response options | | | | Response options | | | | Response options | | | | Response options | | | | Response options | | | | Response options | | | | Response options | | | | Response options | | | | Response options | | | | Response options | | | | Response options | | | | Response options | | | | Response options | | | | Response options | | | | Response options | | | | Response options | | | | Response options | | | | Response options | | | | Response options | | | |  |  |
| Bias due to confounding | |  | 1.1 Is there potential for confounding of the effect of intervention in this study? | | | | | | PY | | | PY | | | | Y | | | | PY | | | | PY | | | | PY | | | | PY | | | | PY | | | | PY | | | | PY | | | | PY | | | | PY | | | | PY | | | | PY | | | | PY | | | | PY | | | | PY | | | | PY | | | | PY | | | | PN | | | | PY | | | | PY | | | | PY | | | | PN | | | | PY | | | |  |
|  |  |  | | | 1.2. Was the analysis based on splitting participants’ follow up time according to intervention received? | | | N | | | N | | | |  | | | | PN | | | | N | | | | N | | | | PN | | | | PN | | | | PN | | | | PN | | | | PN | | | | NA | | | | N | | | | N | | | | PN | | | | N | | | | N | | | | PN | | | | N | | | |  | | | | PN | | | | PN | | | |  | | | |  | | | | PN | | | |  |  |
|  |  |  | | | 1.3. Were intervention discontinuations or switches likely to be related to factors that are prognostic for the outcome? | | N | | | |  | | | |  | | | | N | | | | PN | | | | PN | | | | PN | | | | NI | | | | PN | | | | PN | | | | N | | | | N | | | | PN | | | | PN | | | |  | | | |  | | | |  | | | | PN | | | |  | | | |  | | | | PN | | | |  | | | |  | | | |  | | | | N | | | |  |  |
|  |  | Questions relating to baseline confounding only | | | 1.4. Did the authors use an appropriate analysis method that controlled for all the important confounding domains? | | PY | | | | PY | | | |  | | | | Y | | | | PY | | | | PY | | | | PY | | | | PY | | | | PY | | | | PY | | | | Y | | | | PN | | | | PY | | | | Y | | | | PY | | | | Y | | | | Y | | | | PY | | | | PY | | | |  | | | | PY | | | | PY | | | | PY | | | |  | | | | PY | | | |  |  |
|  |  |  | | | 1.5. If Y/PY to 1.4: Were confounding domains that were controlled for measured validly and reliably by the variables available in this study? | | | | | |  | | | PY | | | |  | | | | PY | | | | PY | | | | PY | | | | PY | | | | PY | | | | PY | | | | PY | | | | PY | | | |  | | | | PY | | | |  | | | |  | | | |  | | | |  | | | |  | | | |  | | | |  | | | | PY | | | | PY | | | | PY | | | |  | | | | PY | | |
|  |  |  |  |  | 1.6. Did the authors control for any post-intervention variables that could have been affected by the intervention? | | PY | | | | PN | | | |  | | | | PY | | | | PY | | | | PY | | | | PY | | | | PY | | | | PY | | | | PY | | | | PY | | | | PY | | | | PY | | | | PY | | | | PN | | | | PY | | | | Y | | | | PY | | | | PY | | | |  | | | |  | | | | PN | | | |  | | | |  | | | | PN | | | |  |  |
|  |  | Questions relating to baseline and time-varying confounding | | | 1.7. Did the authors use an appropriate analysis method that controlled for all the important confounding domains and for time-varying confounding? | |  | | | |  | | | | PN | | | |  | | | |  | | | |  | | | |  | | | |  | | | |  | | | |  | | | |  | | | |  | | | |  | | | |  | | | |  | | | |  | | | |  | | | |  | | | |  | | | |  | | | |  | | | |  | | | |  | | | |  | | | |  | | | |  |  |
|  |  |  |  |  | 1.8. If Y/PY to 1.7: Were confounding domains that were controlled for measured validly and reliably by the variables available in this study? | |  | | | |  | | | | PN | | | |  | | | |  | | | |  | | | |  | | | |  | | | |  | | | |  | | | |  | | | |  | | | |  | | | |  | | | |  | | | |  | | | |  | | | |  | | | |  | | | |  | | | |  | | | |  | | | |  | | | |  | | | |  | | | |  |  |
|  |  |  | | | Optional: What is the predicted direction of bias due to confounding? | |  | | | |  | | | |  | | | |  | | | |  | | | |  | | | |  | | | |  | | | |  | | | |  | | | |  | | | |  | | | |  | | | |  | | | |  | | | |  | | | |  | | | |  | | | |  | | | |  | | | |  | | | |  | | | |  | | | |  | | | |  | | | |  |  |
|  |  | Risk of bias judgement | | |  | | Serious | | | | Moderate | | | | Critical | | | | Serious | | | | Serious | | | | Critical | | | | Critical | | | | Critical | | | | Critical | | | | Critical | | | | Serious | | | | Serious | | | | Serious | | | | Serious | | | | Serious | | | | Serious | | | | Serious | | | | Critical | | | | Serious | | | | Low | | | | Moderate | | | | Serious | | | | Moderate | | | | Low | | | | Serious | | | |  |  |
| Bias in selection of participants into the study |  | | | 2.1. Was selection of participants into the study (or into the analysis) based on participant characteristics observed after the start of intervention? | | PN | | | | PN | | | | PN | | | | PY | | | | PY | | | | Y | | | | PY | | | | PY | | | | Y | | | | PY | | | | PY | | | | Y | | | | PY | | | | PN | | | | PY | | | | PY | | | | Y | | | | Y | | | | Y | | | | PN | | | | PN | | | | N | | | | N | | | | PN | | | | PY | | | |  |  |  |
|  |  | | | 2.2. If Y/PY to 2.1: Were the post-intervention variables that influenced selection likely to be associated with intervention? | |  | | | |  | | | |  | | | | PN | | | | PN | | | | PN | | | | PN | | | | PN | | | | PN | | | | PN | | | | N | | | | PN | | | | PN | | | | PN | | | | PN | | | | N | | | | N | | | | PN | | | | NI | | | |  | | | |  | | | | PN | | | | NI | | | | PN | | | | PN | | | |  |  |  |
|  |  | | | 2.3 If Y/PY to 2.2: Were the post-intervention variables that influenced selection likely to be influenced by the outcome or a cause of the outcome? | |  | | | |  | | | |  | | | | PN | | | | PN | | | | N | | | | N | | | | PN | | | | N | | | | N | | | | PN | | | | PN | | | | PN | | | | PN | | | | PN | | | | PN | | | | N | | | | PN | | | | PN | | | |  | | | |  | | | | PN | | | | PN | | | | PN | | | | PN | | | |  |  |  |
|  |  | | | 2.4. Do start of follow-up and start of intervention coincide for most participants? | | NI | | | | PY | | | | PY | | | | Y | | | | PY | | | | PY | | | | PY | | | | Y | | | | PY | | | | PY | | | | Y | | | | Y | | | | PY | | | | Y | | | | Y | | | | PN | | | | Y | | | | Y | | | | PN | | | | PY | | | | Y | | | | Y | | | | Y | | | | PY | | | | NI | | | |  |  |  |
|  |  | | | 2.5. If Y/PY to 2.2 and 2.3, or N/PN to 2.4: Were adjustment techniques used that are likely to correct for the presence of selection biases? | | NI | | | | PY | | | | NI | | | | NI | | | | PY | | | | PY | | | | NI | | | | PY | | | | PY | | | | PY | | | | PY | | | |  | | | | NI | | | |  | | | |  | | | |  | | | |  | | | |  | | | |  | | | | NI | | | | NI | | | | PY | | | |  | | | | PY | | | | NI | | | |  |  |  |
|  |  | | | Optional: What is the predicted direction of bias due to selection of participants into the study? | |  | | | |  | | | |  | | | |  | | | |  | | | |  | | | |  | | | |  | | | |  | | | |  | | | |  | | | |  | | | |  | | | |  | | | |  | | | |  | | | |  | | | |  | | | |  | | | |  | | | |  | | | |  | | | |  | | | |  | | | |  | | | |  |  |  |
|  | Risk of bias judgement | | |  | | NI | | | | Low | | | | Low | | | | Moderate | | | | Moderate | | | | Moderate | | | | Moderate | | | | Moderate | | | | Moderate | | | | Serious | | | | Serious | | | | Low | | | | Moderate | | | | Low | | | | Low | | | | Moderate | | | | Moderate | | | | Low | | | | Moderate | | | | Low | | | | Low | | | | Moderate | | | | Low | | | | Low | | | | Moderate | | | |  |  |  |
| Bias in classification of interventions |  | | | 3.1 Were intervention groups clearly defined? | | Y | | | | Y | | | | Y | | | | PY | | | | PY | | | | PY | | | | PY | | | | PY | | | | PY | | | | PY | | | | PY | | | | PY | | | | PY | | | | PY | | | | PY | | | | PY | | | | Y | | | | PY | | | | PY | | | | Y | | | | Y | | | | PY | | | | Y | | | | PY | | | | PY | | | |  |  |  |
|  |  | | | 3.2 Was the information used to define intervention groups recorded at the start of the intervention? | | PY | | | | Y | | | | PY | | | | Y | | | | PN | | | | N | | | | PY | | | | PY | | | | PY | | | | PY | | | | N | | | | PY | | | | N | | | | Y | | | | Y | | | | PY | | | | N | | | | PN | | | | PY | | | | Y | | | | PY | | | | PN | | | | Y | | | | Y | | | | PY | | | |  |  |  |
|  |  | | | 3.3 Could classification of intervention status have been affected by knowledge of the outcome or risk of the outcome? | | PY | | | | N | | | | NI | | | | N | | | | PY | | | | PY | | | | PY | | | | NI | | | | PY | | | | N | | | | N | | | | PN | | | | PY | | | | PY | | | | PY | | | | PY | | | | N | | | | PN | | | | NI | | | | N | | | | N | | | | PN | | | | NI | | | | PN | | | | PY | | | |  |  |  |
|  |  | | | Optional: What is the predicted direction of bias due to classification of interventions? | |  | | | |  | | | |  | | | |  | | | |  | | | |  | | | |  | | | |  | | | |  | | | |  | | | |  | | | |  | | | |  | | | |  | | | |  | | | |  | | | |  | | | |  | | | |  | | | |  | | | |  | | | |  | | | |  | | | |  | | | |  | | | |  |  |  |
|  | Risk of bias judgement | | |  | | Moderate | | | | Low | | | | Low | | | | Low | | | | Moderate | | | | Moderate | | | | Low | | | | Moderate | | | | Low | | | | Low | | | | Low | | | | Low | | | | Moderate | | | | Low | | | | Low | | | | Low | | | | Moderate | | | | Moderate | | | | Moderate | | | | Low | | | | Low | | | | Moderate | | | | Low | | | | Low | | | | Moderate | | | |  |  |  |
| Bias due to deviations from intended interventions | If your aim for this study is to assess the effect of assignment to intervention, answer questions 4.1 and 4.2 | | | 4.1. Were there deviations from the intended intervention beyond what would be expected in usual practice? | |  | | | |  | | | |  | | | |  | | | |  | | | |  | | | |  | | | |  | | | |  | | | |  | | | |  | | | | PN | | | |  | | | |  | | | |  | | | |  | | | |  | | | |  | | | |  | | | |  | | | |  | | | |  | | | |  | | | |  | | | |  | | | |  |  |  |
|  |  |  |  | 4.2. If Y/PY to 4.1: Were these deviations from intended intervention unbalanced between groups *and* likely to have affected the outcome? | |  | | | |  | | | |  | | | |  | | | |  | | | |  | | | |  | | | |  | | | |  | | | |  | | | |  | | | | PN | | | |  | | | |  | | | |  | | | |  | | | |  | | | |  | | | |  | | | |  | | | |  | | | |  | | | |  | | | |  | | | |  | | | |  |  |  |
|  | If your aim for this study is to assess the effect of starting and adhering to intervention, answer questions 4.3 to 4.6 | | | 4.3. Were important co-interventions balanced across intervention groups? | | PY | | | | Y | | | | PY | | | | PY | | | | PY | | | | PY | | | | PY | | | | PY | | | | PY | | | | PY | | | | PY | | | |  | | | | PY | | | | PY | | | | PY | | | | PY | | | | PN | | | | PN | | | | PY | | | | NI | | | | N | | | | PY | | | | N | | | | NI | | | | PY | | | |  |  |  |
|  |  |  |  | 4.4. Was the intervention implemented successfully for most participants? | | PY | | | | Y | | | | PY | | | | NI | | | | PY | | | | PY | | | | Y | | | | PY | | | | PY | | | | NI | | | | NI | | | |  | | | | PY | | | | NI | | | | PY | | | | NI | | | | PY | | | | NI | | | | NI | | | | Y | | | | Y | | | | NI | | | | Y | | | | PY | | | | PN | | | |  |  |  |
|  |  |  |  | 4.5. Did study participants adhere to the assigned intervention regimen? | | PY | | | | Y | | | | NI | | | | Y | | | | PY | | | | Y | | | | PY | | | | NI | | | | PY | | | | PY | | | | NI | | | |  | | | | Y | | | | Y | | | | PY | | | | PY | | | | PY | | | | PY | | | | Y | | | | Y | | | | NI | | | | PY | | | | Y | | | | PY | | | | NI | | | |  |  |  |
|  |  |  |  | 4.6. If N/PN to 4.3, 4.4 or 4.5: Was an appropriate analysis used to estimate the effect of starting and adhering to the intervention? | | PY | | | | PY | | | | PY | | | |  | | | |  | | | |  | | | |  | | | |  | | | |  | | | |  | | | |  | | | | PY | | | |  | | | |  | | | |  | | | |  | | | |  | | | |  | | | |  | | | |  | | | |  | | | |  | | | | PY | | | | PY | | | | PY | | | |  |  |  |
|  |  | | | Optional: What is the predicted direction of bias due to deviations from the intended interventions? | |  | | | |  | | | |  | | | |  | | | |  | | | |  | | | |  | | | |  | | | |  | | | |  | | | |  | | | |  | | | |  | | | |  | | | |  | | | |  | | | |  | | | |  | | | |  | | | |  | | | |  | | | |  | | | |  | | | |  | | | |  | | | |  |  |  |
|  | Risk of bias judgement | | |  | | Low | | | | Low | | | | Moderate | | | | Moderate | | | | Low | | | | Low | | | | Low | | | | Low | | | | Low | | | | Low | | | | NI | | | | Low | | | | Low | | | | Low | | | | Low | | | | Low | | | | Moderate | | | | Moderate | | | | Moderate | | | | Low | | | | Low | | | | Low | | | | Low | | | | Low | | | | Moderate | | | |  |  |  |
| Bias due to missing data |  | | | 5.1 Were outcome data available for all, or nearly all, participants? | | PY | | | | Y | | | | PY | | | | PY | | | | PY | | | | PY | | | | PY | | | | PY | | | | PY | | | | PY | | | | PY | | | | PY | | | | PY | | | | PY | | | | PY | | | | PY | | | | PY | | | | Y | | | | PY | | | | Y | | | | Y | | | | PY | | | | PY | | | | PY | | | | PY | | | |  |  |  |
|  |  | | | 5.2 Were participants excluded due to missing data on intervention status? | | Y | | | | Y | | | | PY | | | | PY | | | | Y | | | | Y | | | | Y | | | | PY | | | | Y | | | | PY | | | | Y | | | | Y | | | | PY | | | | PY | | | | Y | | | | Y | | | | Y | | | | PY | | | | PY | | | | PY | | | | Y | | | | PY | | | | Y | | | | Y | | | | Y | | | |  |  |  |
|  |  | | | 5.3 Were participants excluded due to missing data on other variables needed for the analysis? | | PN | | | | PY | | | | PN | | | | Y | | | | PY | | | | PN | | | | PN | | | | PN | | | | PN | | | | N | | | | PN | | | | N | | | | PN | | | | PN | | | | N | | | | PN | | | | NI | | | | PN | | | | Y | | | | PY | | | | PY | | | | NI | | | | Y | | | | Y | | | | NI | | | |  |  |  |
|  |  | | | 5.4 If PN/N to 5.1, or Y/PY to 5.2 or 5.3: Are the proportion of participants and reasons for missing data similar across interventions? | | PY | | | | PY | | | | PN | | | | PN | | | | PY | | | | PY | | | | PY | | | | PY | | | | PY | | | | PN | | | | PY | | | | PY | | | | PN | | | | PY | | | | PN | | | | PY | | | | PN | | | | PY | | | | NI | | | | NI | | | | PN | | | | PN | | | | Y | | | | PY | | | | PN | | | |  |  |  |
|  |  | | | 5.5 If PN/N to 5.1, or Y/PY to 5.2 or 5.3: Is there evidence that results were robust to the presence of missing data? | | PY | | | | PY | | | | PY | | | | PY | | | | PY | | | | PY | | | | PY | | | | PY | | | | PY | | | | PY | | | | PY | | | | PY | | | | PY | | | | PY | | | | PY | | | | PY | | | | PY | | | | PY | | | | PY | | | | PY | | | | PY | | | | PY | | | | PY | | | | PY | | | | PY | | | |  |  |  |
|  |  | | | Optional: What is the predicted direction of bias due to missing data? | |  | | | |  | | | |  | | | |  | | | |  | | | |  | | | |  | | | |  | | | |  | | | |  | | | |  | | | |  | | | |  | | | |  | | | |  | | | |  | | | |  | | | |  | | | |  | | | |  | | | |  | | | |  | | | |  | | | |  | | | |  | | | |  |  |  |
|  | Risk of bias judgement | | |  | | Serious | | | | Moderate | | | | Serious | | | | Serious | | | | Serious | | | | Serious | | | | Serious | | | | Serious | | | | Serious | | | | Serious | | | | Serious | | | | Serious | | | | Critical | | | | Serious | | | | Moderate | | | | Serious | | | | Serious | | | | Serious | | | | Serious | | | | Moderate | | | | Moderate | | | | Moderate | | | | Moderate | | | | Serious | | | | Serious | | | |  |  |  |
| Bias in measurement of outcomes |  | | | 6.1 Could the outcome measure have been influenced by knowledge of the intervention received? | | PY | | | | PY | | | | PY | | | | PY | | | | PY | | | | PY | | | | PY | | | | PY | | | | PY | | | | PY | | | | PY | | | | PY | | | | PY | | | | PY | | | | PY | | | | PY | | | | PY | | | | PY | | | | PY | | | | PY | | | | PY | | | | PY | | | | PY | | | | PY | | | | PY | | | |  |  |  |
|  |  | | | 6.2 Were outcome assessors aware of the intervention received by study participants? | | PY | | | | N | | | | PN | | | | NI | | | | PY | | | | PN | | | | NI | | | | PN | | | | PN | | | | PN | | | | PN | | | | PY | | | | PN | | | | N | | | | NI | | | | PY | | | | PN | | | | PN | | | | PY | | | | NI | | | | PY | | | | NI | | | | PY | | | | PY | | | | PY | | | |  |  |  |
|  |  | | | 6.3 Were the methods of outcome assessment comparable across intervention groups? | | N | | | | N | | | | NI | | | | NI | | | | PN | | | | PN | | | | NI | | | | PN | | | | N | | | | PN | | | | PN | | | | NI | | | | PN | | | | PN | | | | PN | | | | PN | | | | PN | | | | PN | | | | PN | | | | NI | | | | PN | | | | NI | | | | NI | | | | NI | | | | PN | | | |  |  |  |
|  |  | | | 6.4 Were any systematic errors in measurement of the outcome related to intervention received? | | PN | | | | PN | | | | PN | | | | PN | | | | PN | | | | PN | | | | PN | | | | PN | | | | PN | | | | N | | | | PN | | | | PN | | | | PN | | | | PN | | | | PN | | | | PN | | | | N | | | | PN | | | | N | | | | PN | | | | PN | | | | PN | | | | PN | | | | PN | | | | N | | | |  |  |  |
|  |  | | | Optional: What is the predicted direction of bias due to measurement of outcomes? | |  | | | |  | | | |  | | | |  | | | |  | | | |  | | | |  | | | |  | | | |  | | | |  | | | |  | | | |  | | | |  | | | |  | | | |  | | | |  | | | |  | | | |  | | | |  | | | |  | | | |  | | | |  | | | |  | | | |  | | | |  | | | |  |  |  |
|  | Risk of bias judgement | | |  | | Moderate | | | | Moderate | | | | Moderate | | | | Serious | | | | Moderate | | | | Moderate | | | | NI | | | | Moderate | | | | Moderate | | | | Moderate | | | | Moderate | | | | Moderate | | | | Moderate | | | | Moderate | | | | Serious | | | | Moderate | | | | Serious | | | | Serious | | | | Moderate | | | | Moderate | | | | Moderate | | | | Serious | | | | Moderate | | | | Serious | | | | Serious | | | |  |  |  |
| Bias in selection of the reported result |  | | | Is the reported effect estimate likely to be selected, on the basis of the results, from... | |  | | | |  | | | |  | | | |  | | | |  | | | |  | | | |  | | | |  | | | |  | | | |  | | | |  | | | |  | | | |  | | | |  | | | |  | | | |  | | | |  | | | |  | | | |  | | | |  | | | |  | | | |  | | | |  | | | |  | | | |  | | | |  |  |  |
|  |  | | | 7.1. ... multiple outcome *measurements* within the outcome domain? | | NI | | | | N | | | | N | | | | N | | | | PN | | | | NI | | | | PN | | | | PN | | | | N | | | | NI | | | | PN | | | | NI | | | | NI | | | | N | | | | N | | | | N | | | | N | | | | NI | | | | N | | | | NI | | | | N | | | | N | | | | PN | | | | NI | | | | PN | | | |  |  |  |
|  |  | | | 7.2 ... multiple *analyses* of the intervention-outcome relationship? | | NI | | | | N | | | | NI | | | | NI | | | | PN | | | | PN | | | | PN | | | | NI | | | | PN | | | | NI | | | | NI | | | | NI | | | | PN | | | | NI | | | | NI | | | | N | | | | N | | | | N | | | | PN | | | | N | | | | N | | | | NI | | | | PN | | | | PN | | | | NI | | | |  |  |  |
|  |  | | | 7.3 ... different *subgroups*? | | N | | | | N | | | | PN | | | | PN | | | | NI | | | | NI | | | | PN | | | | PN | | | | PN | | | | PN | | | | PN | | | | NI | | | | PN | | | | PN | | | | PN | | | | PN | | | | NI | | | | NI | | | | NI | | | | PN | | | | PN | | | | PN | | | | NI | | | | PN | | | | NI | | | |  |  |  |
|  |  | | | Optional: What is the predicted direction of bias due to selection of the reported result? | |  | | | |  | | | |  | | | |  | | | |  | | | |  | | | |  | | | |  | | | |  | | | |  | | | |  | | | |  | | | |  | | | |  | | | |  | | | |  | | | |  | | | |  | | | |  | | | |  | | | |  | | | |  | | | |  | | | |  | | | |  | | | |  |  |  |
|  | Risk of bias judgement | | |  | | NI | | | | Low | | | | Moderate | | | | Low | | | | Low | | | | NI | | | | Low | | | | Low | | | | Low | | | | NI | | | | Low | | | | NI | | | | Low | | | | Low | | | | Low | | | | Low | | | | Low | | | | NI | | | | Low | | | | Low | | | | Low | | | | Low | | | | Low | | | | Low | | | | NI | | | |  |  |  |
| Overall bias | Risk of bias judgement | | |  | | Serious | | | | Moderate | | | | Critical | | | | Serious | | | | Serious | | | | Critical | | | | Critical | | | | Serious | | | | Critical | | | | Critical | | | | Serious | | | | Serious | | | | Critical | | | | Serious | | | | Serious | | | | Serious | | | | Serious | | | | Critical | | | | Serious | | | | Moderate | | | | Moderate | | | | Serious | | | | Moderate | | | | Serious | | | | Serious | | | |  |  |  |

**Appendix 8. Forest plot of social function**

**
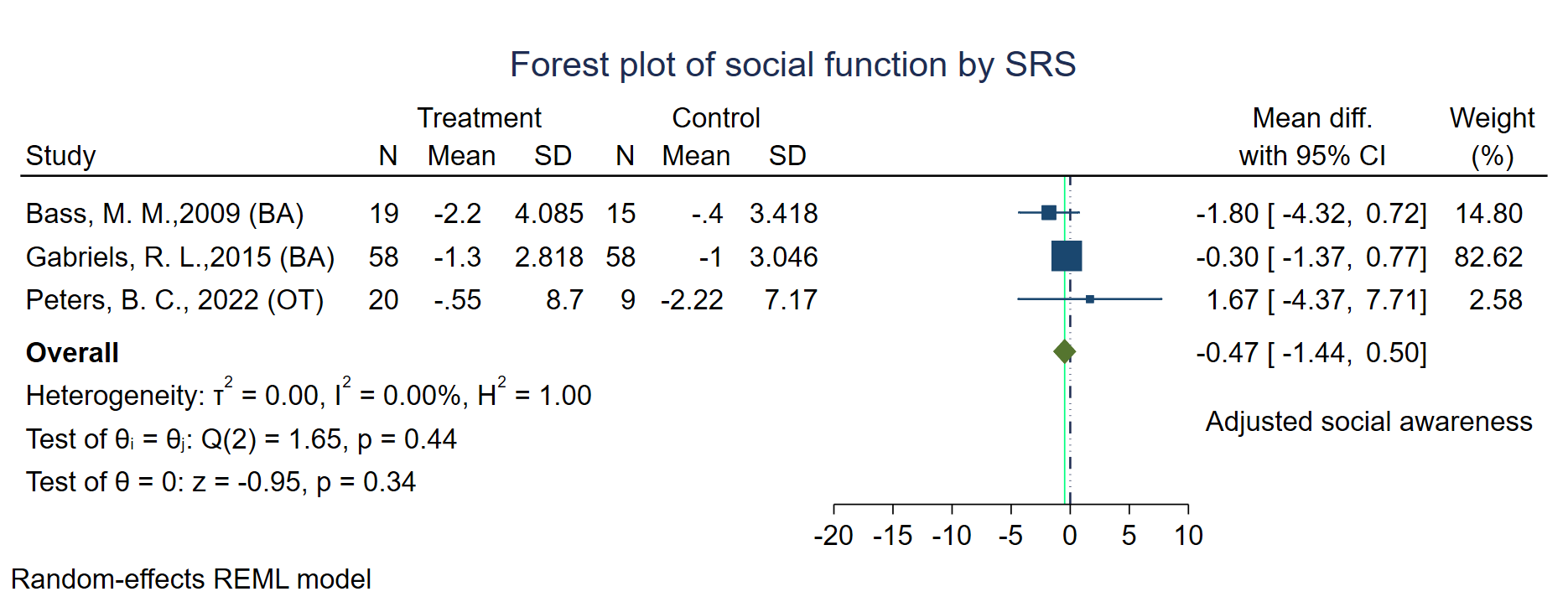
**

**
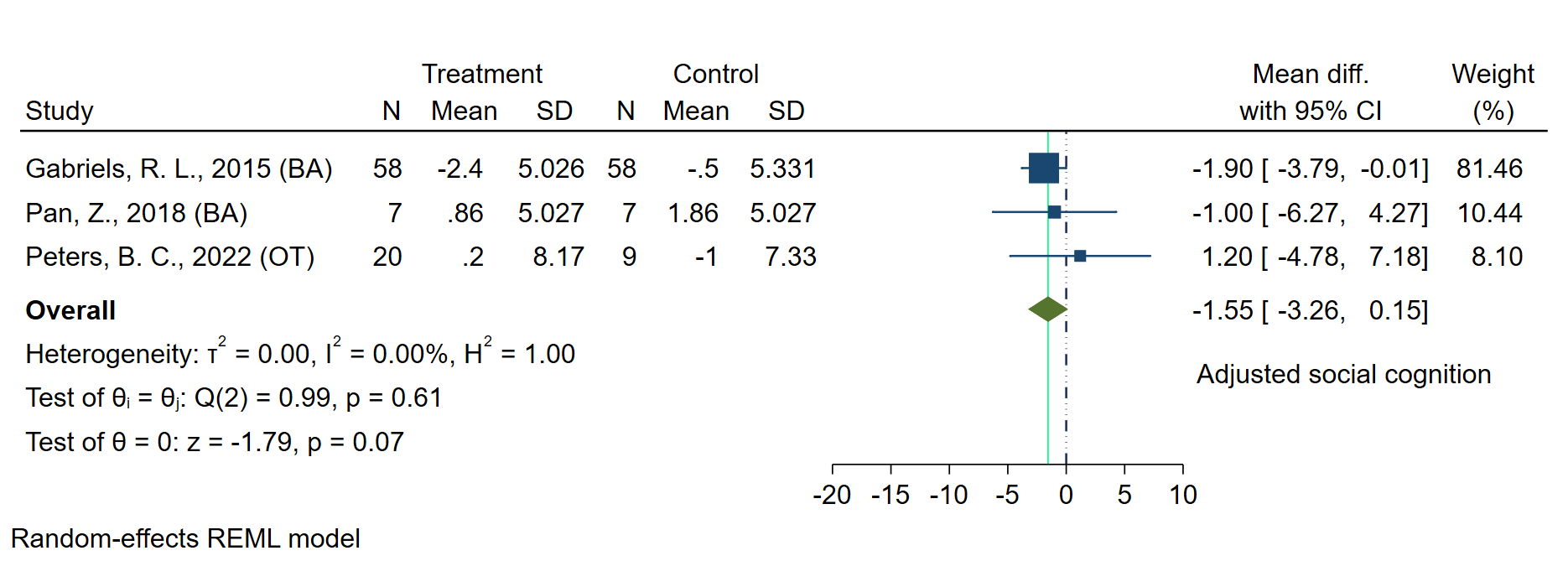
**

**Appendix 9. PRISMA checklist**

| **Section and Topic** | **Item #** | **Checklist item** | **Location where item is reported** |
| --- | --- | --- | --- |
| **TITLE** | | |  |
| Title | 1 | Identify the report as a systematic review. |  |
| **ABSTRACT** | | |  |
| Abstract | 2 | See the PRISMA 2020 for Abstracts checklist. |  |
| **INTRODUCTION** | | |  |
| Rationale | 3 | Describe the rationale for the review in the context of existing knowledge. |  |
| Objectives | 4 | Provide an explicit statement of the objective(s) or question(s) the review addresses. |  |
| **METHODS** | | |  |
| Eligibility criteria | 5 | Specify the inclusion and exclusion criteria for the review and how studies were grouped for the syntheses. |  |
| Information sources | 6 | Specify all databases, registers, websites, organisations, reference lists and other sources searched or consulted to identify studies. Specify the date when each source was last searched or consulted. |  |
| Search strategy | 7 | Present the full search strategies for all databases, registers and websites, including any filters and limits used. |  |
| Selection process | 8 | Specify the methods used to decide whether a study met the inclusion criteria of the review, including how many reviewers screened each record and each report retrieved, whether they worked independently, and if applicable, details of automation tools used in the process. |  |
| Data collection process | 9 | Specify the methods used to collect data from reports, including how many reviewers collected data from each report, whether they worked independently, any processes for obtaining or confirming data from study investigators, and if applicable, details of automation tools used in the process. |  |
| Data items | 10a | List and define all outcomes for which data were sought. Specify whether all results that were compatible with each outcome domain in each study were sought (e.g. for all measures, time points, analyses), and if not, the methods used to decide which results to collect. |  |
|  | 10b | List and define all other variables for which data were sought (e.g. participant and intervention characteristics, funding sources). Describe any assumptions made about any missing or unclear information. |  |
| Study risk of bias assessment | 11 | Specify the methods used to assess risk of bias in the included studies, including details of the tool(s) used, how many reviewers assessed each study and whether they worked independently, and if applicable, details of automation tools used in the process. |  |
| Effect measures | 12 | Specify for each outcome the effect measure(s) (e.g. risk ratio, mean difference) used in the synthesis or presentation of results. |  |
| Synthesis methods | 13a | Describe the processes used to decide which studies were eligible for each synthesis (e.g. tabulating the study intervention characteristics and comparing against the planned groups for each synthesis (item #5)). |  |
|  | 13b | Describe any methods required to prepare the data for presentation or synthesis, such as handling of missing summary statistics, or data conversions. |  |
|  | 13c | Describe any methods used to tabulate or visually display results of individual studies and syntheses. |  |
|  | 13d | Describe any methods used to synthesize results and provide a rationale for the choice(s). If meta-analysis was performed, describe the model(s), method(s) to identify the presence and extent of statistical heterogeneity, and software package(s) used. |  |
|  | 13e | Describe any methods used to explore possible causes of heterogeneity among study results (e.g. subgroup analysis, meta-regression). |  |
|  | 13f | Describe any sensitivity analyses conducted to assess robustness of the synthesized results. |  |
| Reporting bias assessment | 14 | Describe any methods used to assess risk of bias due to missing results in a synthesis (arising from reporting biases). |  |
| Certainty assessment | 15 | Describe any methods used to assess certainty (or confidence) in the body of evidence for an outcome. |  |
| **RESULTS** | | |  |
| Study selection | 16a | Describe the results of the search and selection process, from the number of records identified in the search to the number of studies included in the review, ideally using a flow diagram. |  |
|  | 16b | Cite studies that might appear to meet the inclusion criteria, but which were excluded, and explain why they were excluded. |  |
| Study characteristics | 17 | Cite each included study and present its characteristics. |  |
| Risk of bias in studies | 18 | Present assessments of risk of bias for each included study. |  |
| Results of individual studies | 19 | For all outcomes, present, for each study: (a) summary statistics for each group (where appropriate) and (b) an effect estimate and its precision (e.g. confidence/credible interval), ideally using structured tables or plots. |  |
| Results of syntheses | 20a | For each synthesis, briefly summarise the characteristics and risk of bias among contributing studies. |  |
|  | 20b | Present results of all statistical syntheses conducted. If meta-analysis was done, present for each the summary estimate and its precision (e.g. confidence/credible interval) and measures of statistical heterogeneity. If comparing groups, describe the direction of the effect. |  |
|  | 20c | Present results of all investigations of possible causes of heterogeneity among study results. |  |
|  | 20d | Present results of all sensitivity analyses conducted to assess the robustness of the synthesized results. |  |
| Reporting biases | 21 | Present assessments of risk of bias due to missing results (arising from reporting biases) for each synthesis assessed. |  |
| Certainty of evidence | 22 | Present assessments of certainty (or confidence) in the body of evidence for each outcome assessed. |  |
| **DISCUSSION** | | |  |
| Discussion | 23a | Provide a general interpretation of the results in the context of other evidence. |  |
|  | 23b | Discuss any limitations of the evidence included in the review. |  |
|  | 23c | Discuss any limitations of the review processes used. |  |
|  | 23d | Discuss implications of the results for practice, policy, and future research. |  |
| **OTHER INFORMATION** | | |  |
| Registration and protocol | 24a | Provide registration information for the review, including register name and registration number, or state that the review was not registered. |  |
|  | 24b | Indicate where the review protocol can be accessed, or state that a protocol was not prepared. |  |
|  | 24c | Describe and explain any amendments to information provided at registration or in the protocol. |  |
| Support | 25 | Describe sources of financial or non-financial support for the review, and the role of the funders or sponsors in the review. |  |
| Competing interests | 26 | Declare any competing interests of review authors. |  |
| Availability of data, code and other materials | 27 | Report which of the following are publicly available and where they can be found: template data collection forms; data extracted from included studies; data used for all analyses; analytic code; any other materials used in the review. |  |
